# The Computational Study of the Mechanism of the Acid-Promoted Pyranoside-*into*-Furanoside Rearrangement

**DOI:** 10.3390/molecules31183145

**Published:** 2026-09-08

**Authors:** Alexey G. Gerbst, Dmitry A. Argunov, Vadim B. Krylov, Nikolay E. Nifantiev

**Affiliations:** Laboratory of Glycoconjugate Chemistry, N.D. Zelinsky Institute of Organic Chemistry, Russian Academy of Sciences, Leninsky Prospect 47, 119991 Moscow, Russiav_krylov@ioc.ac.ru (V.B.K.)

**Keywords:** pyranoside-*into*-furanoside rearrangement, benzoylated galactosides, mechanism, DFT, DLPNO-CCSD(T)

## Abstract

The pyranoside-*into*-furanoside (PIF) rearrangement is an uncommon but important process in carbohydrate chemistry. The quantum chemical investigation of the driving force of the recently discovered TfOH-catalyzed ring contraction revealed that it stemmed from the π–π interactions of the phenyl rings in benzoyl-protecting groups. In this study, we focused on the kinetic aspects, which included preliminary 2-O-benzoyl group rotation followed by the protonation of the endo-cyclic O5 atom. Using a combination of DFT and DLPNO-CCSDT methods, we found that in some cases, DFT may not produce adequate energies at the rate-limiting stage of the pyranoside ring opening, presumably due to inadequate modeling of Van der Waals interactions. Predicted rate constants for the PIF rearrangement of the β-O-methyl and β-S-ethyl galactosides were in agreement with NMR kinetic experiments, as the latter reacts significantly slower. The estimated constant for the β-O-phenyl galactoside supports its inability to undergo ring contraction and suggests temperatures of over 400 K for such transformation. The proposed mechanism was additionally confirmed by substituting the triflic acid with the much weaker trifluoroacetic one, which led to a drastic decrease of the reaction rate both in computations and in the experiment.

## 1. Introduction

**Figure 1:**
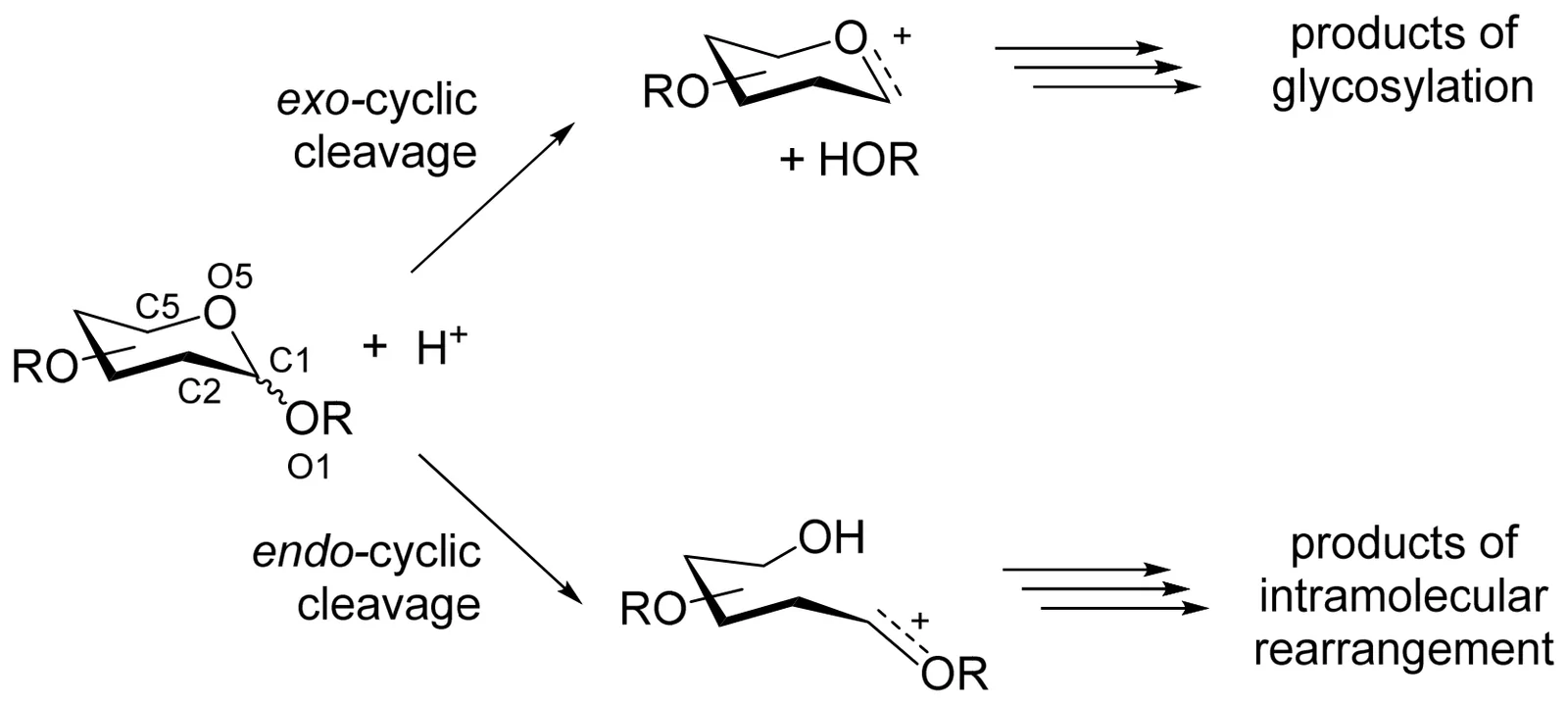
Two possible pathways of a reaction at the anomeric center: attack on O1 leading to glycosylation products, and attack on O5 probably leading to the ring opening.

Carbohydrates play an important role in many biochemical processes [1,2,3]. Hence, the increased interest in understanding their synthesis and transformations [4,5,6,7]. Their molecules represent quite complex subjects due to the combination of the protective groups that may influence reactivity in synthetic intermediates and the presence of the acetal fragment commonly called the anomeric center [8,9,10]. Particularly, the latter is responsible for the most practical reactions in carbohydrate chemistry, such as glycosylations [11,12,13]. As can be seen, these transformations involve the *exo*-cyclic oxygen atom, O1 (Figure 1). These types of reactions have been widely studied both experimentally and theoretically [14,15,16,17]. Theoretical approaches included the DFT study of the oxacarbenium ion formation [18,19] and an investigation of the electronic density distribution in glycosides employing a combination of DFT and NBO methods [20]. Additionally, the QM-cluster approach [21] and combined QM-MM methods [22,23], along with the pure DFT [24], can be applied to study the glycoside hydrolysis.

Thus, it is seen that the reactions whose mechanism primarily includes an attack at the O1 *exo*-cyclic atom have been widely studied [11,12,13]. However, examples of reactions resulting from an attack on the *endo*-cyclic oxygen atom, O5, are also known [25,26,27,28]. Several years ago, a new type of carbohydrate reactivity was discovered by our group: a pyranoside ring contraction to the furanoside form upon the total sulfation of unprotected β-galactosides [29]. It was utilized as a key step for the preparation of blocks applied in the assembling of complex oligosaccharides related to antigenic cell-wall polysaccharides of pathogenic fungi and bacteria, including *Aspergillus fumigatus* [30], *Klebsiella pneumonia* [31], and *Enterococcus faecalis* [32], as well as a fucoidan of brown seaweed *Chordaria flagelliformis* [33]. It was also shown that PIF can be applied for the rearrangement of certain galactopyranoside units within a preassembled oligosaccharide chain [34].

**Figure 2:**
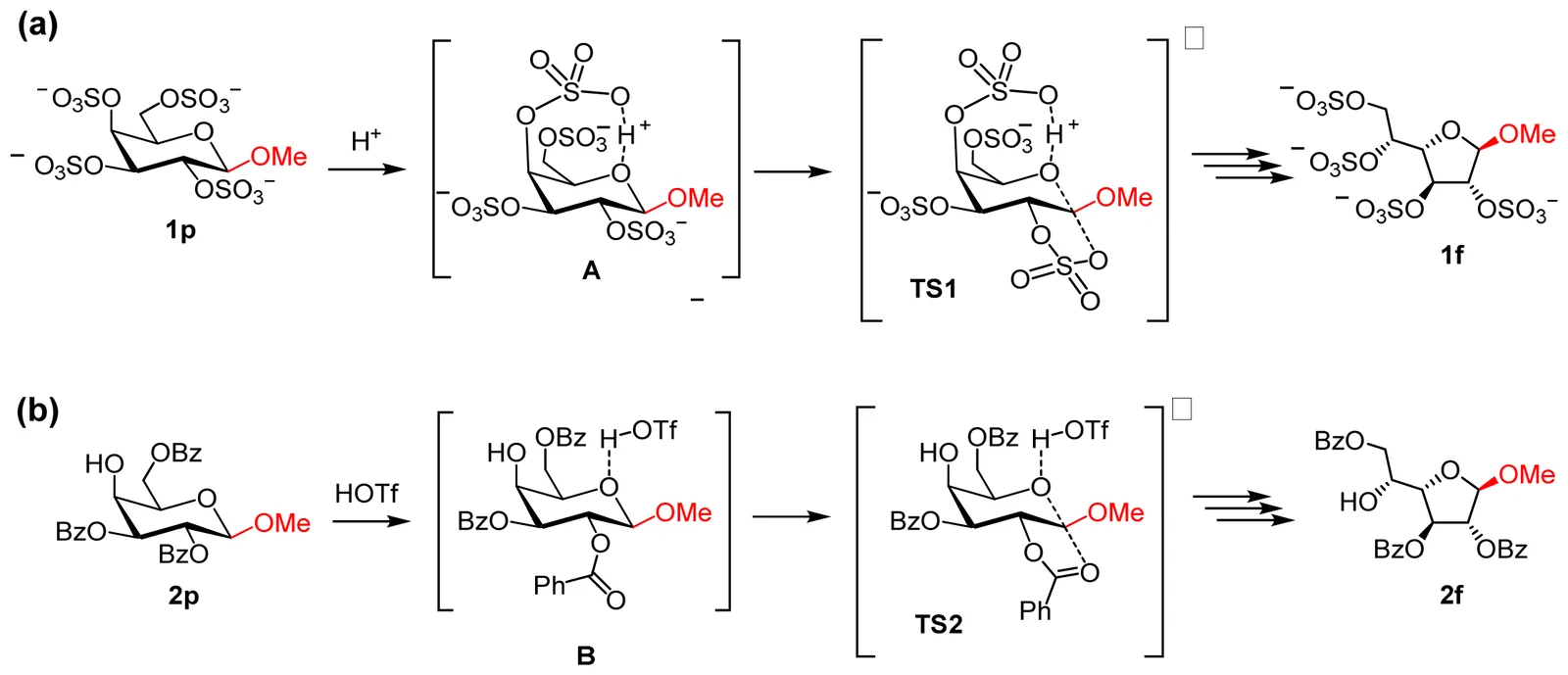
The plausible mechanism of the PIF rearrangement (**a**) under the exhaustive sulfation and (**b**) the acid catalysis.

**Figure 3:**
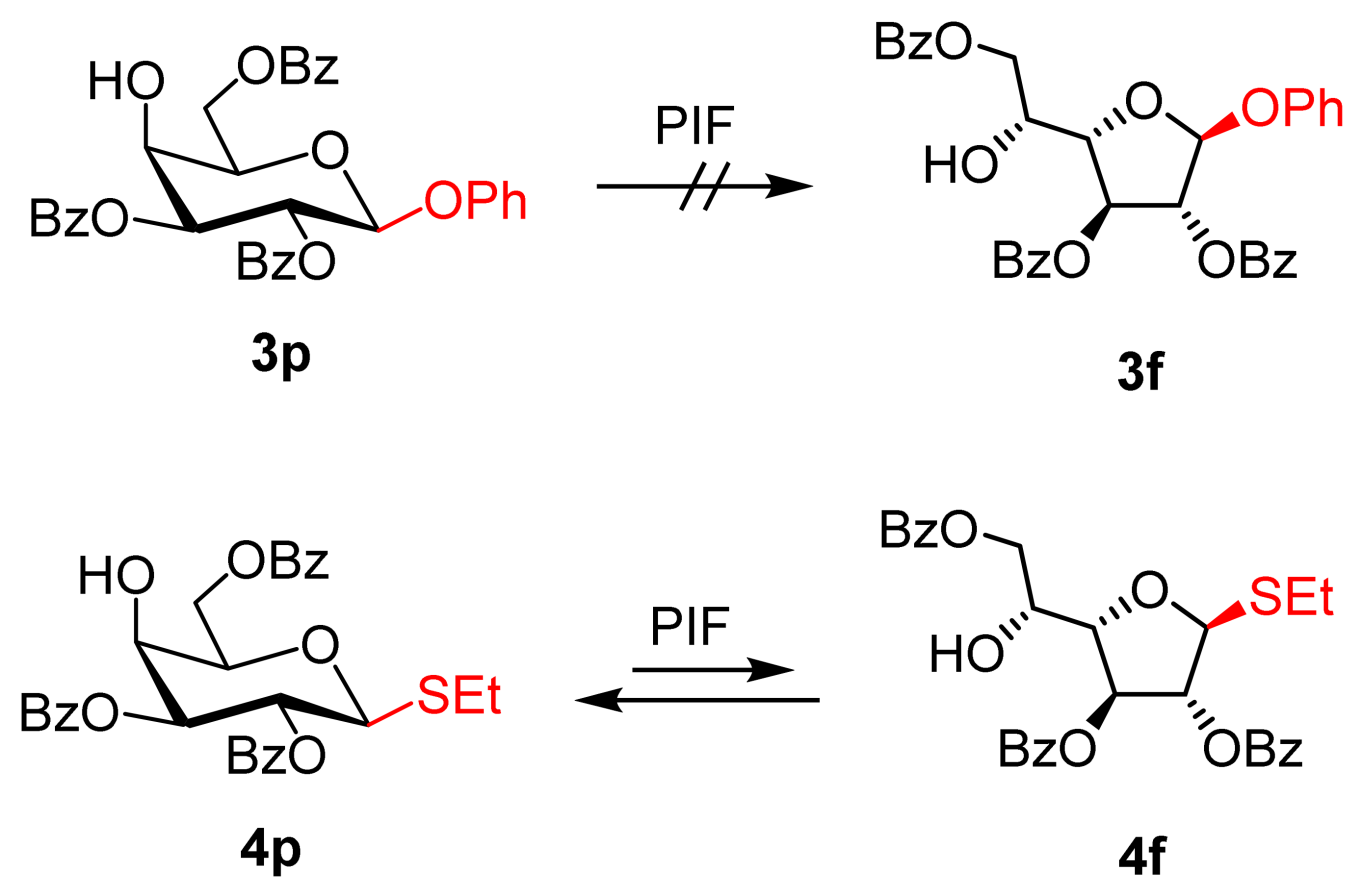
The structures of the galactosides considered in this work. PIF: TfOH (cat.), CH_2_Cl_2_, r.t.

It seemed obvious to suggest a mechanism for this reaction [35] based on the involvement of the O5 oxygen atom, which led to the C1-O5 bond cleavage, permitting subsequent ring contraction [36] (Figure 2a). Furthermore, the possibility of this pyranoside-*into*-furanoside (PIF) transformation under the action of catalytic amounts of triflic acid was revealed [31], and its possible driving force was studied [37] (Figure 2b). The study included saccharides **2p**–**4p** (Figure 3). It was demonstrated that they all should undergo the PIF rearrangement [37]. However, β-O-phenyl galactopyranoside **3p** did not react at all, while the transforfdomation of **2p** proceeded smoothly. Hence, it was decided to examine this situation in the mechanistic aspect using a combination of DFT and DLPNO-CCSD(T) approaches, and the results are reported below. For the DFT calculations, B3LYP functional with D3 correction was employed (see **Materials and Methods**) since it was previously found to demonstrate most reliable results for the conformational analysis of oxacarbenium ions [38].

## 2. Results and Discussion

In the present work, three β-galactosides—**2p**, **3p**, and **4p**—were the subject of the investigation (Figure 2 and Figure 3). The former two saccharides were discussed previously, while thiogalactoside **4p** was added to the study as an example of slower rearrangement and predomination of pyranoside form in equilibrium.

In the course of the previous studies of PIF rearrangement promoted by exhaustive sulfation, we suggested a mechanism illustrated in Figure 2a. It included the protonation of endocyclic oxygen atom O5, which can be regarded as stabilized by the axially oriented sulfate at O4. After that, a C1-O5 bond cleavage occurred, assisted by the negatively charged sulfate group at O2. This is obviously followed by the ring opening and closure to form a furanoside isomer.

In the present case, it seemed straightforward to suppose the analogous way of the PIF transformation: First, protonation of O5 by the proton donated from the triflic acid, then bond breaking and ring opening/closure (Figure 2b) through the assistance by carbonyl oxygen at O2.

**Figure 4:**
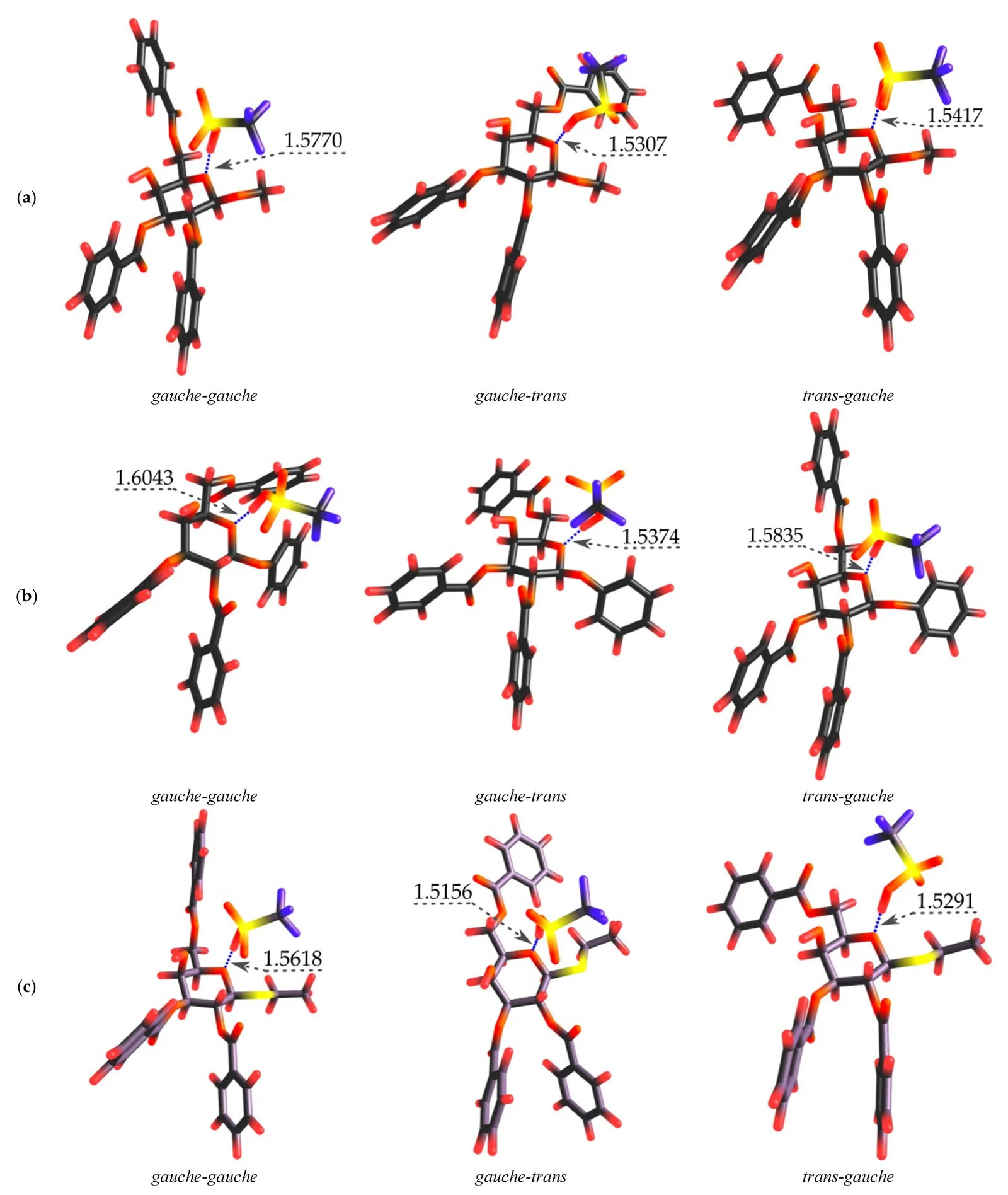
Starting conformations of the TfOH-galactoside complexes: (**a**) for structure **2p**; (**b**) for structure **3p**; (**c**) for structure **4p**. Numbers above arrows are O5···H^+^ distances given in Å.

At the beginning of our study, β-O-methyl galactoside **2p** was investigated. The geometry optimization of its complex with a molecule of TfOH yielded its three starting conformations, differed by the ω (O5-C5-C6-O6) torsion (Figure 4a). Notably, complete dissociation of the triflic acid was not observed in any of these structures. The distance between O5 and the proton of the triflic acid moiety varied in the range ca. 1.53–1.60 Å.

**Figure 5:**
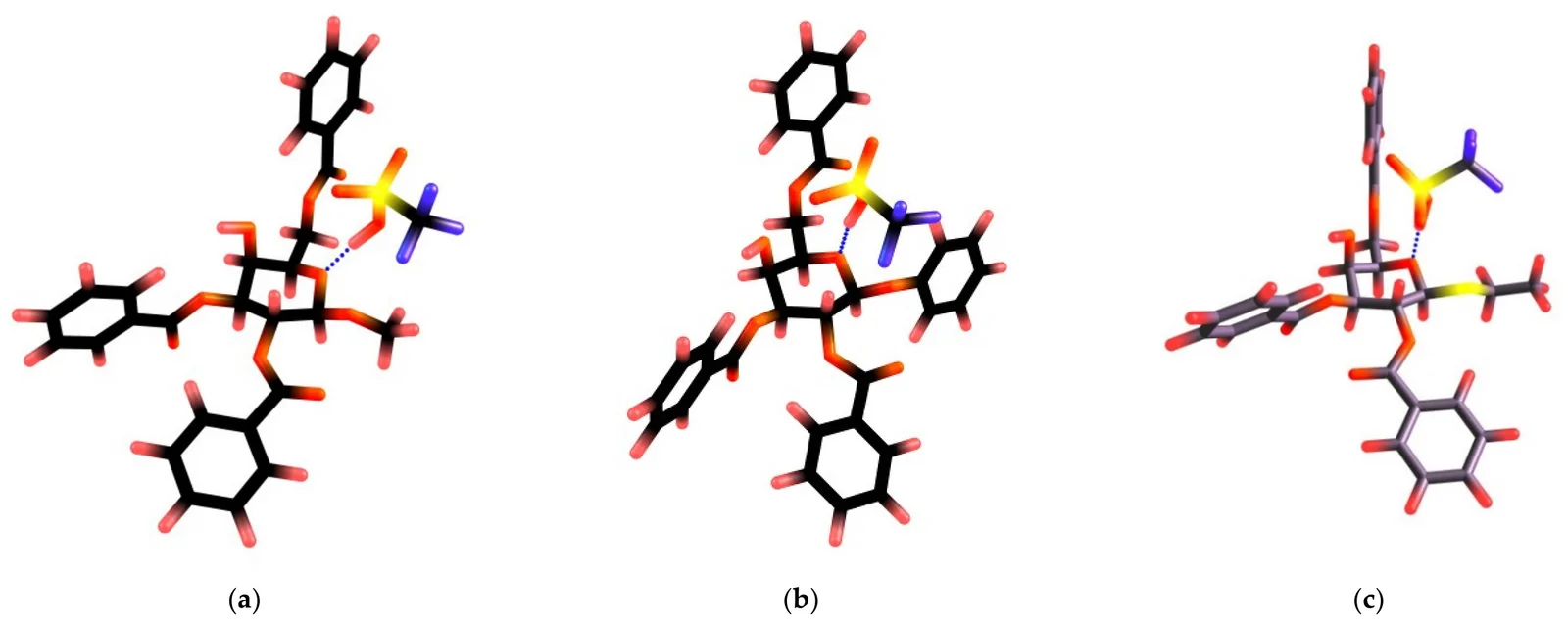
Transition states of the O2 benzoyl group rotation for *gg* conformers of **2p** (**a**), **3p** (**b**), and **4p** (**c**). Note the counter direction of the benzoyl rotation for **4p**.

It should also be mentioned that a benzoate group at O2 in carbohydrates is commonly assumed to be oriented as in the obtained structures. That is, the carbonyl oxygen atom in this substituent is in the position that does not allow it to participate in C1-O1 bond cleavage by the stabilization of the partial positive charge appearing on the C1 atom during this process. Thus, the next stage of the work was to determine the barrier of the O2 benzoate group rotation in order to make its carbonyl fragment closer to the anomeric center (C1). This was accomplished, first, by performing a relaxed potential energy surface (PES) scan of the H2-C2-O2-C_carbonyl_ torsional angle from its optimized position towards −120°. The maximum of the energy curve was found to be about −80°, and this was used as a starting point for the saddle point search. Finally, when such a conformation was obtained and only one imaginary vibrational mode was found for it, indeed corresponding to the rotation of the benzoate group, Gibbs free energy of activation was computed relative to the starting conformation (Table 1). For the *gg* conformer of structure **1**, it was found to be 8.0 kcal/mole; the graphical representation is given in Figure 5a. The last conformation obtained during the relaxed PES scan was subjected to geometry optimization to yield the conformation in which the stabilizing interaction between the carbonyl oxygen atom and the anomeric center was possible. This conformation was in turn used as a starting point for the search of the transition state (TS) of the C1-O5 bond cleavage.

**Figure 6:**
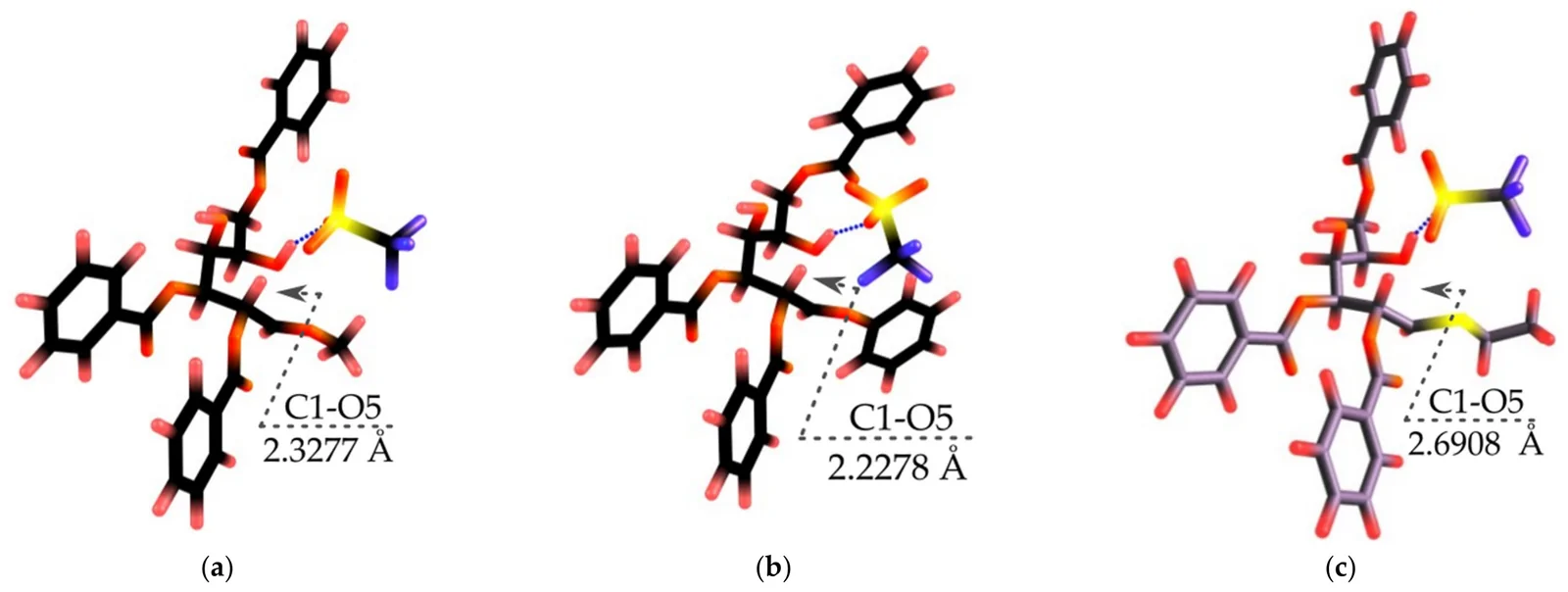
Transition states of the C1-O5 bond cleavage for *gg* conformers of **2p** (**a**), **3p** (**b**), and **4p** (**c**).

This time, the PES scan was performed for this bond starting from its optimized length (ca. 1.45 Å) to the value of 3 Å with a step value of approximately 0.1 Å. The maximum on the energy curve was found to be 2.23 Å. Successful optimization to the saddle point with a single imaginary vibrational mode corresponding to the C1-O5 bond breaking produced the desired TS (Figure 6a). The value of Gibbs free energy of the barrier was computed relative to the first conformation in this scan (Table 1). The procedure described above was repeated for each of the starting conformations of all three galactosides. The results are summarized in Figure 4, Figure 5 and Figure 6 and Table 1. Interestingly, we failed to locate the TS of the benzoyl rotation in the case of molecule **4p** in the same direction as **2p** and **3p**. This fact is most likely due to the spatial hindrances caused by the sulfur atom and is depicted in Figure 5c. Furthermore, data for the *gt* conformer of structure **4p** are missing because in that case, the optimization of the TS for the C1-O5 bond breaking failed to converge to a correct saddle point.

The first thing that attracts attention upon the examination of the data in Table 1 is that the values of the barriers for the benzoate rotation and the bond breaking are very close to each other in every case. Moreover, the calculated barriers correspond to reactions proceeding quickly at room temperature, which is natural for a side group rotation, but can hardly be expected for a bond cleavage. Additionally, the previously established reaction rate constant for the PIF transformation of galactoside **2p** was 0.40 h^−1^: the reaction actually proceeds in dozens of minutes [31].

Hence, it was decided to refine the obtained results by performing single point DLPNO-CCDS(T) calculations on all the found conformations. For the computation of the Gibbs free energies of the barriers, the corresponding thermodynamic corrections to the electronic energy from the DFT runs were applied. The results are shown in Table 2.

It can be seen that the values of the activation free energies for the benzoate rotation remain generally comparable to those calculated at the DFT level. Meanwhile, the barrier values for the bond-breaking process significantly increase in the DLPNO-CCSD(T) approximation. The difference between the barriers of rotation and the bond breaking definitely demonstrate the rate-limiting nature of the latter step. We suppose that such an effect might be related to a rather long (more than 2.2 Å) distance between C1 and O5 atoms in the TS. This can result in the appearance of some Van der Waals interactions, which are poorly described at the DFT level, while the DLPNO-CCSD(T) fixes the problem. Moreover, this finding is consistent with previous results [39,40] that DFT functionals, and particularly B3LYP-D3, may underestimate reaction barriers. It should also be noted that examination of the largest amplitudes in the coupled cluster calculations revealed that just one of them had a value of 0.06–0.07, while all the rest were below 0.05. This signifies that our structures could be described by the single-reference approach, and no additional calculations were needed.

For β-O-methyl galactoside **2p**, the lowest barrier is found for the *gg* conformer and equals 14.3 kcal/mole. From the above-mentioned experimental rate constant of 0.40 h^−1^, the barrier of 13.0 kcal/mole is calculated according to the reversed form of Eyring Equation (1), where K is the rate constant, T—absolute temperature, R—gas constant, and k and h—Boltzmann and Planck constants, respectively:


(1) $${K=e}^{-\frac{∆G^{\neq }}{RT}}\cdot \frac{kT}{h},$$

This can be regarded as a very good coincidence. Boltzmann distribution including all other conformers was not performed because the previous study demonstrated that the *gauche-gauche* was the highest energy conformer [23]. Thus, consideration of other conformers would lead to even higher computed ΔG^≠^ values.

For β-O-phenyl galactoside **3p**, with its lowest barrier for the *gt* conformer being 18.1 kcal/mole according to the same equation, temperatures of more than 400 K are required for the PIF transformation to reach the comparable reaction rate. However, acid-induced side reactions are expected to predominate over the ring contraction at this temperature. Indirect evidence for the correctness of the described ΔG^≠^ calculations for the phenyl galactoside could be obtained by performing its reverse transformation, since it was expected to proceed even slower.

**Figure 7:**
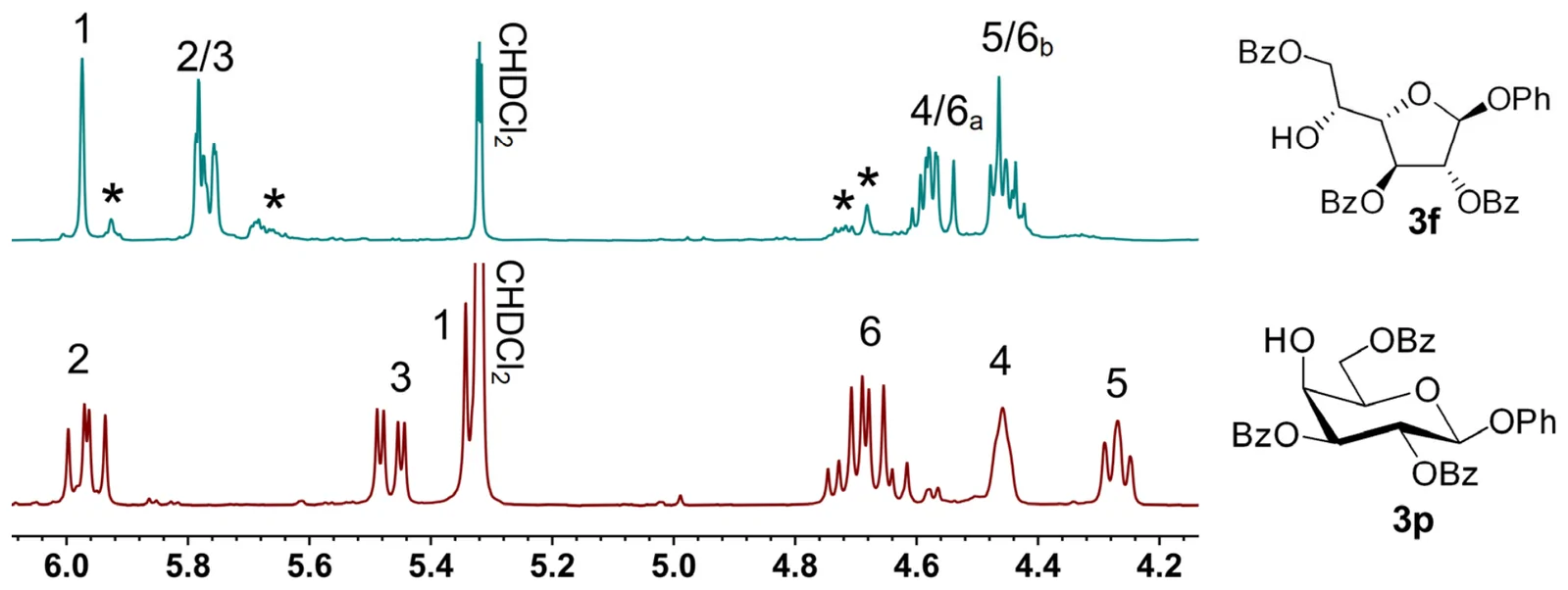
^1^H NMR spectrum of galactofuranoside **3f** treated with TfOH (**upper**) in comparison with the spectrum of the pure galactopyranoside **3p** (**lower**). Asterisks denote the signals belonging to products of the furanoside destruction.

To test the feasibility of reverse furanoside to pyranoside conversion, the solution of phenyl galactofuranoside **3f** in CD_2_Cl_2_ (600 μL) was treated with TfOH (0.1 μL). NMR monitoring of this reaction mixture over 16 h showed only products of acid-induced side reactions like phenyl cleavage, benzoates migration, and other related destructive transformations. No formation of the parent pyranoside was observed (Figure 7). Similar results with much faster acid-promoted destruction were observed when an increased amount of acid (0.5 μL) was used.

**Figure 8:**
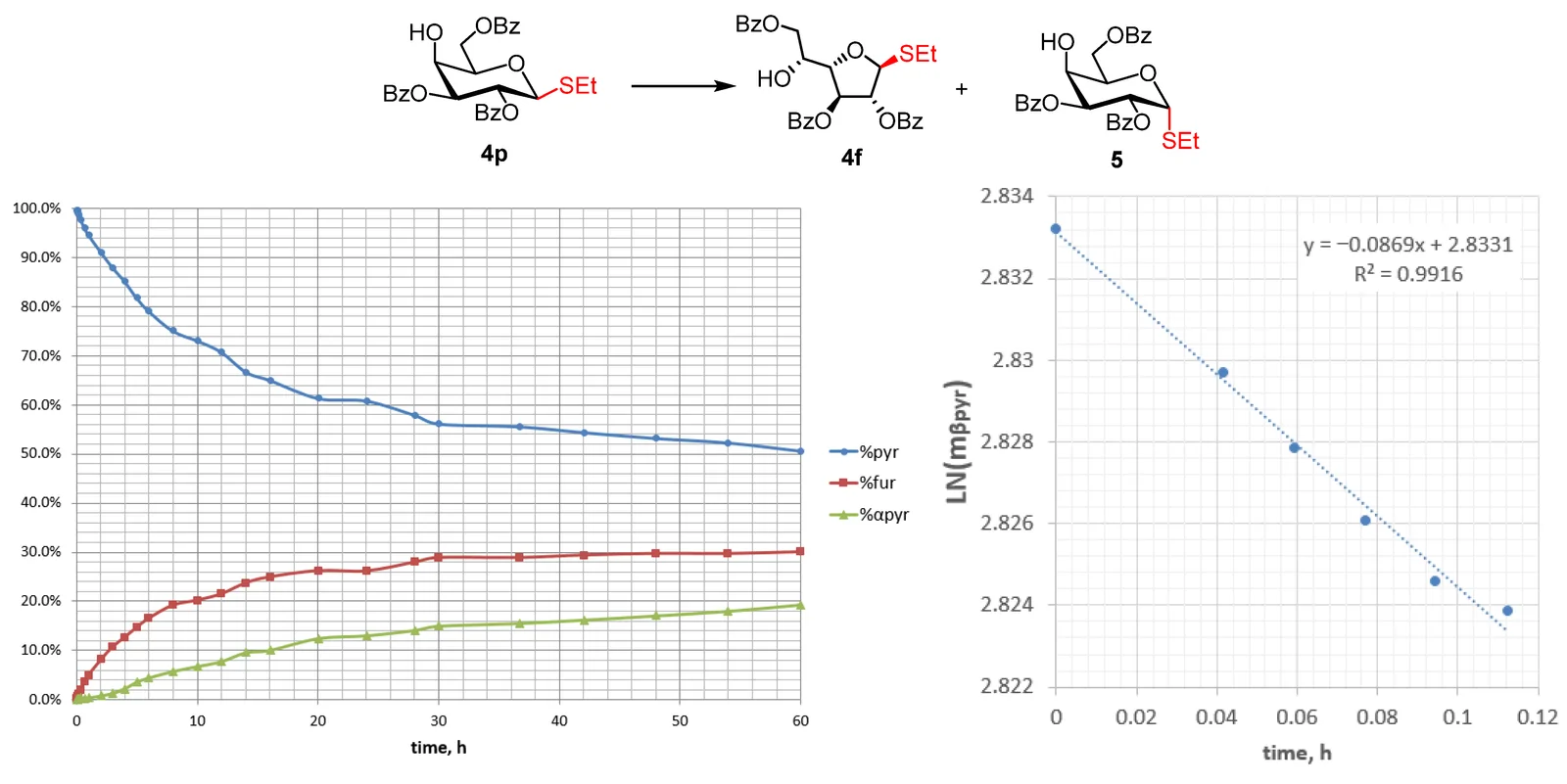
Kinetic curves (**left**) and estimation of the reaction rate constant (**right**) for the PIF rearrangement of structure **4p**. Conditions: 600 µL CD_2_Cl_2_, 0.1 µL TfOH, 298 K.

For β-S-ethyl galactopyranoside **4p**, the PIF transformation proceeded considerably slower than in the case of pyranoside **2p**, in accordance with the calculation results. Additionally, careful examination of reaction mixtures (24 h and longer) revealed the formation of a significant amount of α-pyranoside isomer **5**. Notably, the same side reaction was observed when furanoside **4f** was subjected to same conditions. Moreover, due to even lower reverse transformation speed, the furanoside form still predominated even after two days. Only increased TfOH concentration (0.25 µL) brought back pyranoside domination, which was observed in direct process. The kinetics of the isomerization of **5p** are presented in Figure 8 (for the reverse furanoside transformation, see Supporting Information). The ΔG^≠^ calculated from the experimentally obtained rate constant (0.09 h^−1^) equaled 14.0 kcal/mole, which, although it does not coincide quantitatively with the lowest barrier for this structure from Table 2 (16.9 kcal/mole), still reproduced the tendency predicted from the calculations at the DLPNO-CCSD(T) level. The source of the observed discrepancy is most likely in the effects related to the solvent consideration. Since the continuum model was employed in this study, it may fail in the description of some solvent-specific effects, particularly occurring during the initial complex formation between the substrate and the catalyst. As mentioned previously, consideration of other conformers in the equilibrium would have only increased the computed ΔG^≠^ value.

To further elucidate the role of the O2 benzoate substituent in the C1-O5 breaking process, the natural atomic charges were calculated both for starting conformations of structures **2p**–**4p** (after the rotation of the benzoate group) and in the transition state. The charges on the carbonyl oxygen, anomeric C1, and *endo*-cyclic O5 oxygene atoms are, of course, of the greatest interest. They are summarized in Table 3. It can be seen that, indeed, the charge on the carbonyl oxygen significantly decreases in TS, while the charges on C1 and O5 evidence the bond polarization. TSs corresponding to structures **2p** and **3p** lead to the development of an additional positive charge on C1. In structure **4p**, the charge on C1 is always slightly negative, possibly due to the electron-donating properties of the sulfur atom in the aglycon. These data confirm the hypothesis that the carbonyl group of the benzoate substituent at O2 facilitates the bond cleavage between C1 and O5.

After that, we decided to revisit the PIF rearrangement occurring at the exhaustive sulfation conditions (structure **1p** in Figure 2a) and performed the calculations of its TS using the same approach as above, except that DMF was considered as a solvent instead of CH_2_Cl_2_. The *gauche-gauche* conformer was excluded from consideration, since the presence of the bulky charged sulfate group at O4 makes it obviously unlikely to appear in large amounts in the conformational equilibrium. The results are given in Table 4. Again, it is seen that the DFT approach provides incredibly low absolute values for ΔG^≠^ of the bond cleavage process, while using the DLPNO-CCSD(T) method drastically changes the situation.

The role of the acidic catalyst was additionally evaluated by changing the triflic acid to trifluoroacetic acid (TFA) in case of compound **2p**. The computed energy of the C1-O5 bond cleavage in this case was around 30 kcal/mole, indicating that no reaction should occur in this case. Accordingly, when a solution of methyl 2,3,6-tri-O-benzoyl-β-D-galactopyranoside **2p** (31 mg, 0.060 mmol) in CD_2_Cl_2_ (600 μL) 10 μL of TFA was added, ^1^H NMR spectrum showed no visible changes after 1 h, and around 5% conversion after 24 h. It should be noted that the mentioned amount of TFA is about 100 times greater than that of TfOH used in the previous experiments. Only using 140 μL of TFA in 460 µL of CD_2_Cl_2_, we were able to reach the rate of TfOH-catalyzed transformation; however, spectra quality significantly degraded, and the yields of isolated furanoside product were generally lower than described earlier for the TfOH-catalyzed procedure.

## 3. Materials and Methods

All the calculations were performed using the ORCA 5.0.4 software package [41]. DFT B3LYP functional with the third order Grimme’s correction (D3) [42] and def2-TZVP [43] basis set were employed. CPCM model [44] with the default parameters for methylene chloride was used to account for bulk solvent effects. The defgrid3 option was used throughout the calculations. The optimized structures were subjected to hessian calculations, and the resulting Gibbs free energies and their corrections to the electronic energies were used for the analysis in this work. For DLPNO-CCSD(T) calculations, the CC-pVTZ basis set was used. Natural atomic charges were computed using NBO 7 software [45] with B3LYP-D3/def2-TZVP wave functions.

## 4. Conclusions

The PIF transformation of three galactosides has been studied by computational methods in this work. The plausibility of the mechanism suggested for it involving the participation of the *endo*-cyclic oxygen atom was confirmed by the calculations. The obtained results provide explanation for the fact that β-phenyl galactoside **3p** does not rearrange at room temperature, contrary to β-methyl derivative **2p**, while the previous thermodynamic studies predicted its transformation. For structure **3p**, the calculations predicted the possibility of its transformation at temperatures exceeding 400 K, at which point decomposition products are expected to dominate.

It is shown that the DFT/B3LYP-D3 approach predicts incorrect and even unrealistic barriers for the C1-O5 bond-breaking process, while the application of the high-level DLPNO-CCSD(T) approximation fixes the situation. The difference between the barriers of rotation and the C1-O5 bond breaking clearly demonstrates that the latter stage is rate limiting. For β-methyl galactoside **2p** and β-methyl 1-thiogalactoside **4p**, the lowest predicted barriers coincide well with that that can be calculated from the experimentally determined reaction rate constants.

The used approach was also applied to the totally sulfated galactoside **1p**, previously studied with rough Hartree–Fock methods, with which the obtained results were in agreement.

The results reported herein also definitely demonstrate that the reaction rate critically depends on the strength of the acid involved, which provides additional evidence for the correctness of the proposed PIF mechanism. Furthermore, we can probably predict feasibility of new substrate ring contractions prior to actual chemical synthesis and test reactions.

## Figures and Tables

**Table 1:** The values of barriers of the benzoyl rotation at O2 and C1-O5 bond breaking calculated at DFT/B3LYP-D3 level of theory.

Structure	Conformer	ΔG^≠^ of Benzoate Rotation, kcal/mole	ΔG^≠^ of C1-O5 Bond Breaking, kcal/mole
**2p**	*gg*	8.0	7.3
	*gt*	9.5	9.3
	*tg*	7.7	8.0
**3p**	*gg*	9.6	10.3
	*gt*	8.1	11.3
	*tg*	9.4	12.3
**4p**	*gg*	8.2	11.3
	*gt*	- *	- **
	*tg*	10.7	11.8

* Calculations were not carried out; ** Calculations failed to converge to a correct transition state.

**Table 2:** The values of barriers of the benzoyl rotation at O2 and C1-O5 bond breaking calculated at DLPNO-CCSD(T) level of theory.

Structure	Conformer	ΔG^≠^ of Benzoate Rotation, kcal/mole	ΔG^≠^ of C1-O5 Bond Breaking, kcal/mole
**2p**	*gg*	8.3	14.3
	*gt*	10.2	16.6
	*tg*	9.7	15.8
**3p**	*gg*	10.4	19.1
	*gt*	7.3	18.1
	*tg*	9.4	19.6
**4p**	*gg*	8.0	16.9
	*gt*	- *	- **
	*tg*	12.1	18.8

* Calculations were not carried out; ** Calculations failed to converge to a correct transition state.

**Table 3:** The values of natural atomic charges on carbonyl oxygens at O2, C1, and O5 atoms as calculated with NBO 7.0 program in the starting and transition states for structures **2p**–**4p**.

Structure	Conformer	Natural Atomic Charge (e) on Atoms:		
		O(carbonyl)	C1	O5
**2p/2p (bond breaking TS)**	*gg*	−0.620/−0.570	0.347/0.501	−0.554/−0.720
	*gt*	−0.621/−0571	0.347/0.503	−0.544/−0.718
	*tg*	−0.621/−0.577	0.348/0.504	−0.543/−0.711
**3p/3p (bond breaking TS)**	*gg*	−0.623/−0.572	0.347/0.502	−0.546/−0.702
	*gt*	−0.621/−0.539	0.347/0.484	−0.546/−0.731
	*tg*	−0.623/−0.572	0.349/0.498	−0.545/−0.701
**4p/4p (bond breaking TS)**	*gg*	−0.621/−0.491	−0.077/−0.110	−0.543/−0.743
	*gt*	- *	- *	- *
	*tg*	−0.618/−0.487	−0.077/−0.116	−0.539/−0.741

* Calculations were not carried out.

**Table 4:** The values of barriers of C1-O5 bond breaking calculated at the DFT/B3LYP-D3 and DLPNO-CCSD(T) levels of theory for structure **1p**.

Structure	Conformer	ΔG^≠^ of C1-O5 Bond Breaking, kcal/mole, at DFT/B3LYP-D3 Level	ΔG^≠^ of C1-O5 Bond Breaking, kcal/mole, at DLPNO-CCSD(T) Level
**1p**	*gt*	9.8	15.2
	*tg*	9.1	14.7

## Data Availability

The original contributions presented in this study are included in the article/Supplementary Materials. Further inquiries can be directed to the corresponding authors.

## Supplementary Materials

The following supporting information can be downloaded at https://www.mdpi.com/article/10.3390/molecules31183145/s1, General methods; NMR data; Details of kinetic experiments; Cartesian coordinates end absolute DLPNO-CCSD(T) energies.

## Abbreviations

The following abbreviations are used in this manuscript:

TFA	Trifluoroacetic acid
TfOH	Trifluoromethanesulfonic acid
PIF	Pyranoside-*into*-furanoside
DMF	Dimethyl formamide
TS	Transition state
PES	Potential energy surface
QM	Quantum mechanics
MM	Molecular mechanics
NBO	Natural bond orbitals
