# Supplementary material for: The Computational Study of the Mechanism of the Acid-Promoted Pyranoside-into-Furanoside Rearrangement
Source: Molecules. 2026 Sep 8;31(18):3145. doi: 10.3390/molecules31183145 (PMC13609050; doi:10.3390/molecules31183145)
Supplement: Supplementary file 1 [file molecules-31-03145-s001.zip › molecules-4527422-supplementary.pdf]

The Computational Study of the Mechanism of the Acid Promoted Pyranoside-*into*-Furanoside Rearrangement

Alexey G. Gerbst, Dmitry A. Argunov, Vadim B. Krylov and Nikolay E. Nifantiev

Contents

General methods and information 2

NMR assignments for studied compounds 2

Ethyl 2,3,6-tri-*O*-benzoyl-1-thio-β-D-galactopyranoside 4p 2

Ethyl 2,3,6-tri-*O*-benzoyl-1-thio-β-D-galactofuranoside 4f 2

Ethyl 2,3,6-tri-*O*-benzoyl-1-thio-α-D-galactopyranoside 5 2

Phenyl 2,3,6-tri-*O*-benzoyl-β-D-galactopyranoside 3p 3

Phenyl 2,3,6-tri-*O*-benzoyl-β-D-galactofuranoside 3f 3

Kinetic experiments 4

Ethyl 2,3,6-tri-*O*-benzoyl-1-thio-β-D-galactopyranoside 4p kinetics (0.1 μL TfOH) 4

Ethyl 2,3,6-tri-*O*-benzoyl-1-thio-β-D-galactofuranoside 4f kinetics (0.1 μL TfOH) 5

Ethyl 2,3,6-tri-*O*-benzoyl-1-thio-β-D-galactofuranoside 4f kinetics (0.25 μL TfOH) 6

Methyl 2,3,6-tri-*O*-benzoyl-β-D-galactopyranoside 2p (10 μL TFA + 590 μL CD_2Cl_2) 7

Methyl 2,3,6-tri-*O*-benzoyl-β-D-galactopyranoside 2p (140 μL TFA + 460 μL CD_2Cl_2) 8

Copies of NMR spectra 9

Phenyl 2,3,6-tri-*O*-benzoyl-β-D-galactofuranoside 9

Phenyl 2,3,6-tri-*O*-benzoyl-β-D-galactofuranoside 10

Ethyl 2,3,6-tri-*O*-benzoyl-1-thio-β-D-galactofuranoside 11

Ethyl 2,3,6-tri-*O*-benzoyl-1-thio-β-D-galactofuranoside (0.25 μL TfOH 24 h) 12

Cartesian coordinates (Å) and CCSDT energies (Eh) of the studied structures. 15

General methods and information

NMR spectra were recorded on Bruker Fourier 300HD (300 MHz) or Bruker Avance Neo (300 MHz) at 298-300 K. The resonance assignment in ^{1}H and ^{13}C NMR spectra was performed using 2D-experiments (COSY, HSQC for most compounds, HSQC-TOCSY for complex mixtures). Chemical shifts are reported in ppm referenced to residual signal of $CHDCl_2$ (δ = 5.32 ppm for ^{1}H) and solvent signal (δ = 53.84 ppm for ^{13}C). For NMR-monitoring 20- 30 mg of substrate was dissolved in CD_2Cl_2, the solution transferred to the NMR-tube and then 10 μL of the freshly prepared TfOH solution in MeCN was added (concentration depends on desired amount of acid in particular experiment). The samples were stored at 25 °C during the reaction process.

NMR assignments for studied compounds

Ethyl 2,3,6-tri-*O*-benzoyl-1-thio-β-D-galactopyranoside 4p

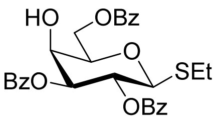

^{1}H NMR (300 MHz, CD_2Cl_2) δ 8.08 – 7.92 (m, 6H, Ph), 7.64 – 7.35 (m, 9H, Ph), 5.75 (t, *J* = 9.9 Hz, 1H, H-2), 5.42 (dd, *J* = 9.9, 3.1 Hz, 1H, H-3), 4.81 (d, *J* = 10.0 Hz, 1H, H-1), 4.65 (dd, *J* = 11.5, 6.0 Hz, 1H, H-6_a), 4.57 (dd, *J* = 11.5, 6.7 Hz, 1H, H-6_b), 4.44 – 4.38 (m, 1H, H-4), 4.13 (ddd, *J* = 6.8, 6.1, 1.0 Hz, 1H, H-5), 2.86 – 2.63 (m, 3H, SC*H*$_2$, OH), 1.25 (t, *J* = 7.4 Hz, 3H, SCH_2CH_3). ^{13}C NMR (75 MHz, CD_2Cl_2) δ 84.32 (C-1), 76.51 (C-5), 75.43 (C-3), 68.49 (C-2), 67.94 (C-4), 63.58 (C-6).

Ethyl 2,3,6-tri-*O*-benzoyl-1-thio-β-D-galactofuranoside 4f

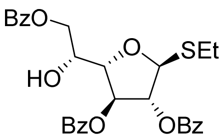

^{1}H NMR (300 MHz, CD_2Cl_2) δ 8.11 – 8.01 (m, 6H, Ph), 7.66 – 7.40 (m, 9H, Ph), 5.68 (ddd, *J* = 5.2, 1.7 Hz, 1H, H-3), 5.63 (s, 1H, H-1), 5.53 (t, *J* = 1.5 Hz, 1H, H-2), 4.60 – 4.52 (m, 2H, H-6_a, H-4), 4.49 – 4.41 (m, 2H, H-6_b, H-5), 2.83 – 2.60 (m, 3H, SC*H*$_2$, OH), 1.30 (t, *J* = 7.4 Hz, 3H, SCH_2CH_3). ^{13}C NMR (75 MHz, CD_2Cl_2) δ 134.03, 133.99, 133.51, 130.28, 130.18, 129.98, 128.98, 128.82, 88.69 (C-1), 82.99 (C-4), 82.84 (C-2), 78.73 (C-3), 69.33 (C-5), 66.51 (C-6), 25.64 (SCH_2), 15.11 (SCH_2CH_3).

Ethyl 2,3,6-tri-*O*-benzoyl-1-thio-α-D-galactopyranoside 5

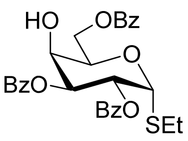

Selected signals from reaction mixture: ^{1}H NMR (300 MHz, CD_2Cl_2) δ 5.93 (d, *J* = 5.7 Hz, 1H, H-1), 5.84 (dd, *J* = 10.5, 5.7 Hz, 2H, H-2), 5.62 (dd, *J* = 10.5, 3.2 Hz, 1H, H-3), 4.82 – 4.76 (m, 1H, H-5), 4.43 (dd, *J* = 3.6, 1.1 Hz, 1H, H-4). ^{13}C NMR (75 MHz, CD_2Cl_2) δ 82.59 (C-1), 71.72 (C-3), 68.97 (C-2), 68.69 (C-5), 68.49 (C-4), 63.88 (C-6).

Phenyl 2,3,6-tri-*O*-benzoyl-β-D-galactopyranoside 3p

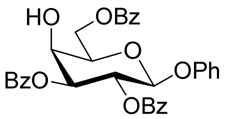

^{1}H NMR (300 MHz, CD_2Cl_2) δ 8.10 – 7.95 (m, 6H), 7.67 – 7.34 (m, 9H), 7.22 – 7.14 (m, 2H), 7.05 – 6.98 (m, 3H), 5.97 (dd, *J* = 10.3, 8.0 Hz, 1H, H-2), 5.47 (dd, *J* = 10.3, 3.2 Hz, 1H, H-3), 5.33 (d, *J* = 8.0 Hz, 1H, H-1), 4.72 (dd, *J* = 11.6, 5.4 Hz, 1H, H-6_a), 4.65 (dd, *J* = 11.6, 7.3 Hz, 1H, H-6_b), 4.48 – 4.44 (m, 1H, H-4), 4.27 (ddd, *J* = 7.4, 5.6, 1.0 Hz, 1H, H-5), 2.68 (d, *J* = 5.1 Hz, 1H, OH). ^{13}C NMR (75 MHz, CD_2Cl_2) δ 133.96, 133.73, 133.68, 130.14, 130.04, 129.87, 128.94, 128.92, 128.80, 123.55, 117.50, 100.25, 74.46, 73.21, 69.80, 67.75, 63.40.

Phenyl 2,3,6-tri-*O*-benzoyl-β-D-galactofuranoside 3f

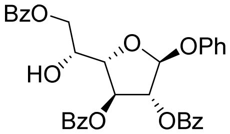

^{1}H NMR (300 MHz, CD_2Cl_2) δ 8.15 – 7.97 (m, 6H), 7.69 – 7.01 (m, 14H), 5.98 (s, 1H, H-1), 5.79 (d, *J* = 1.6 Hz, 1H, H-2), 5.77 (dd, *J* = 4.9, 1.6 Hz, 1H, H-3), 4.62 – 4.53 (m, 2H, H-4, H-6_a), 4.50 – 4.41 (m, 2H, H-5, H-6_b), 2.75 (d, *J* = 7.6 Hz, 1H, OH). ^{13}C NMR (75 MHz, CD_2Cl_2) δ 134.09, 134.06, 133.49, 130.31, 130.27, 130.24, 129.98, 129.03, 128.99, 128.81, 123.07, 117.30, 104.77 (C-1), 84.58 (C-4), 82.12 (C-2), 78.29 (C-3), 69.45 (C-5), 66.47 (C-6).

Kinetic experiments

Ethyl 2,3,6-tri-*O*-benzoyl-1-thio-β-D-galactopyranoside 4p kinetics (0.1 µL TfOH)

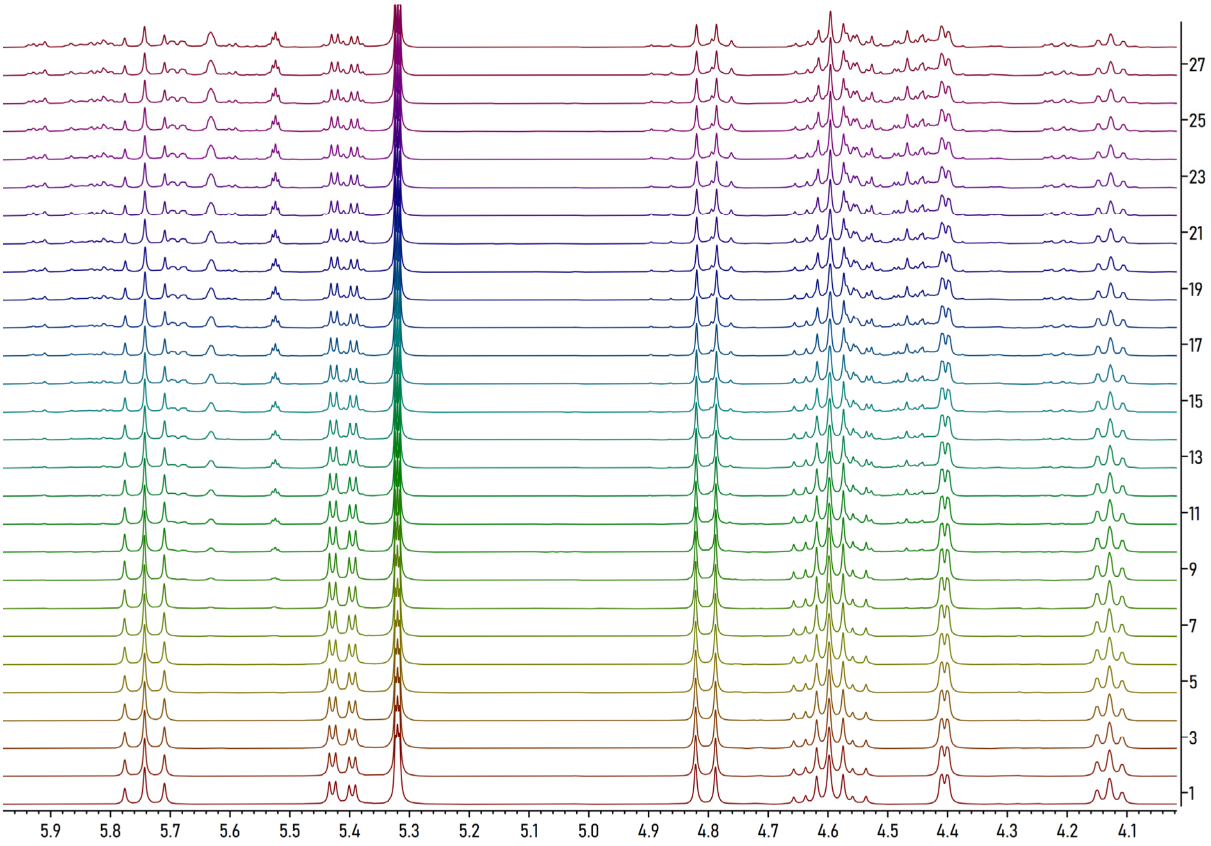

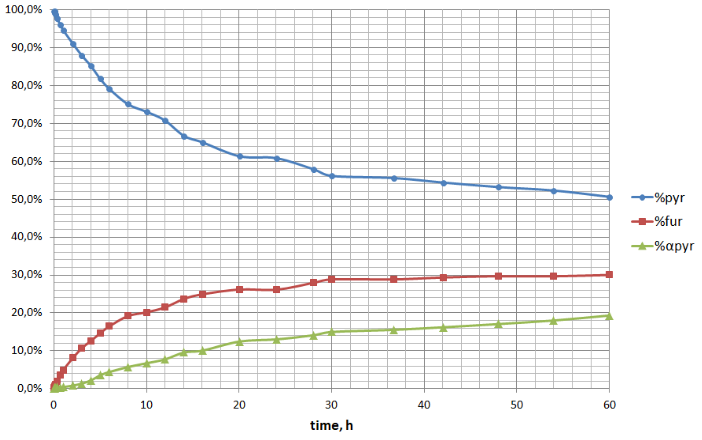

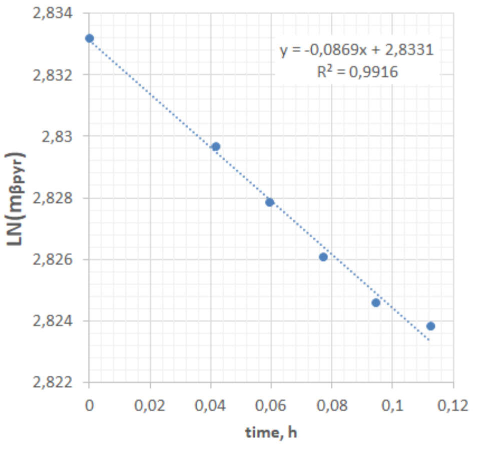

Integrated signals: H-5 (βpyr), H-2 (fur), H-1 (αpyr).

Ethyl 2,3,6-tri-*O*-benzoyl-1-thio-β-D-galactofuranoside 4f kinetics (0.1 μL TfOH)

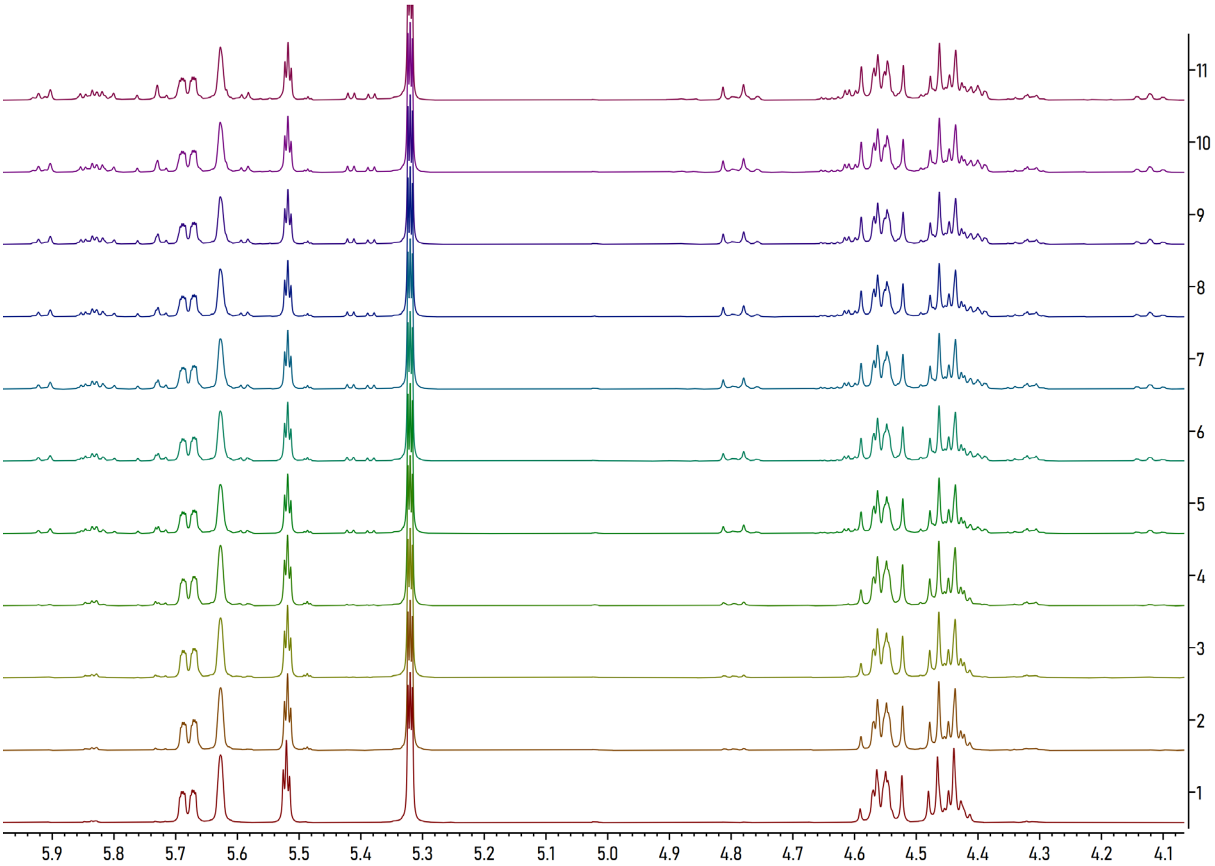

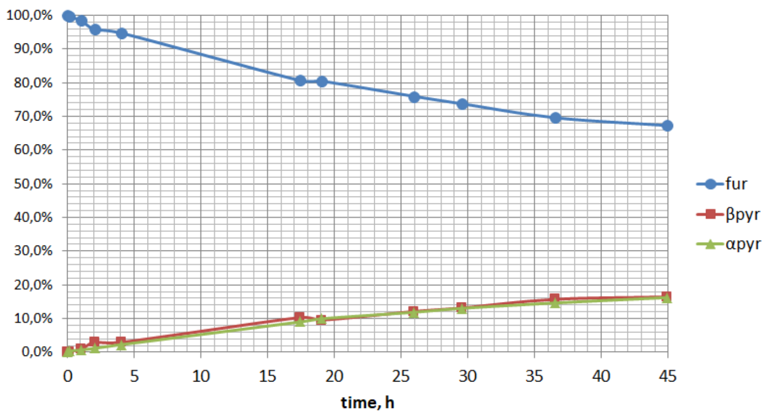

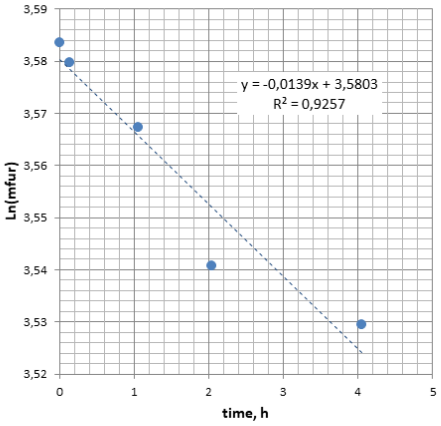

Integrated signals: H-3 (βpyr), H-2 (fur), H-1 (αpyr).

Ethyl 2,3,6-tri-*O*-benzoyl-1-thio-β-D-galactofuranoside 4f kinetics (0.25 μL TfOH)

α-pyranoside form integral also includes subsequently formed product of benzoate migration from O-3 to O-4.

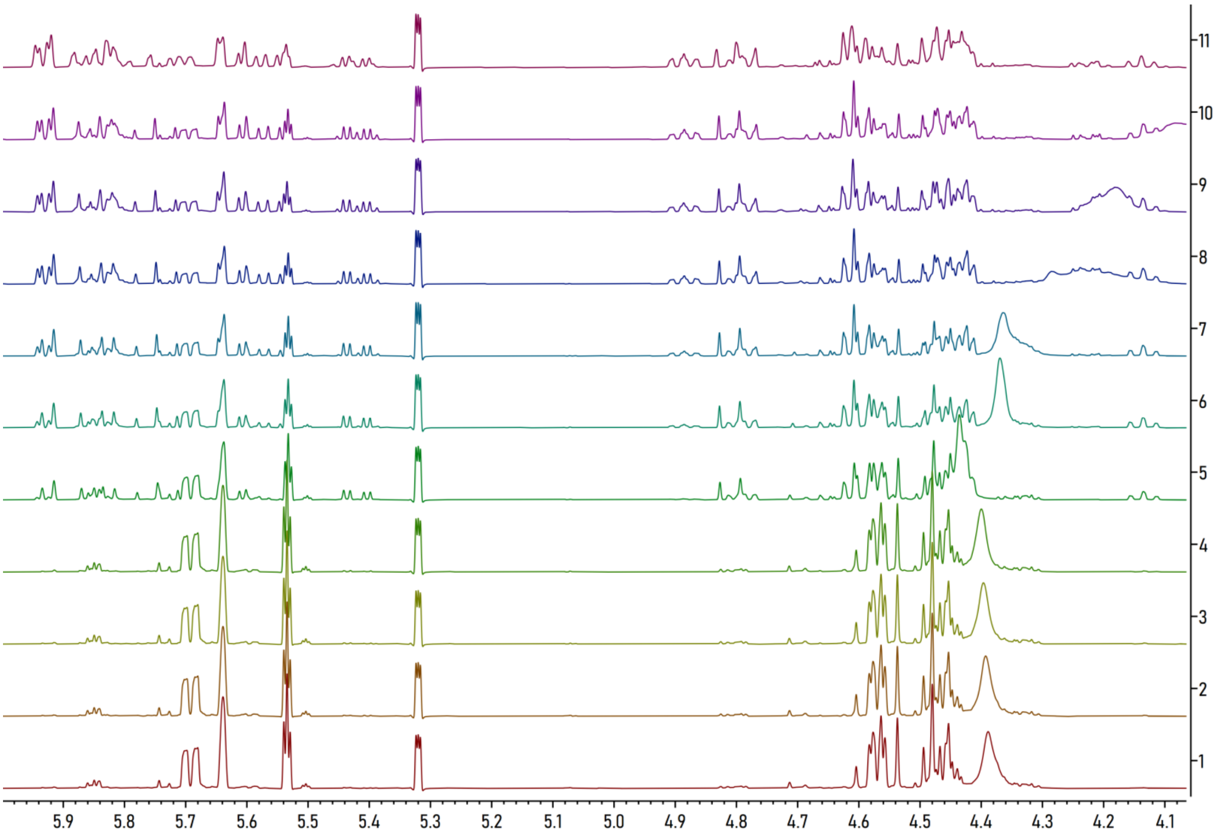

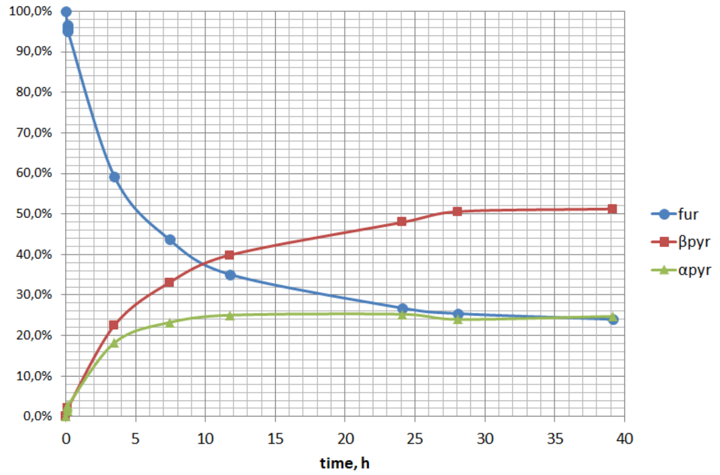

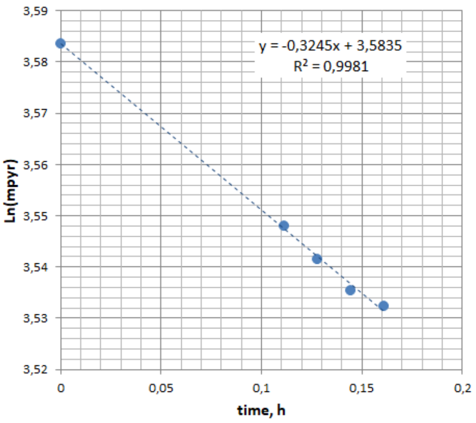

Integrated signals: H-3 (βpyr), H-2 (fur), H-1 (αpyr).

Methyl 2,3,6-tri-*O*-benzoyl-β-D-galactopyranoside 2p (10 μL TFA + 590 μL CD_2Cl_2)

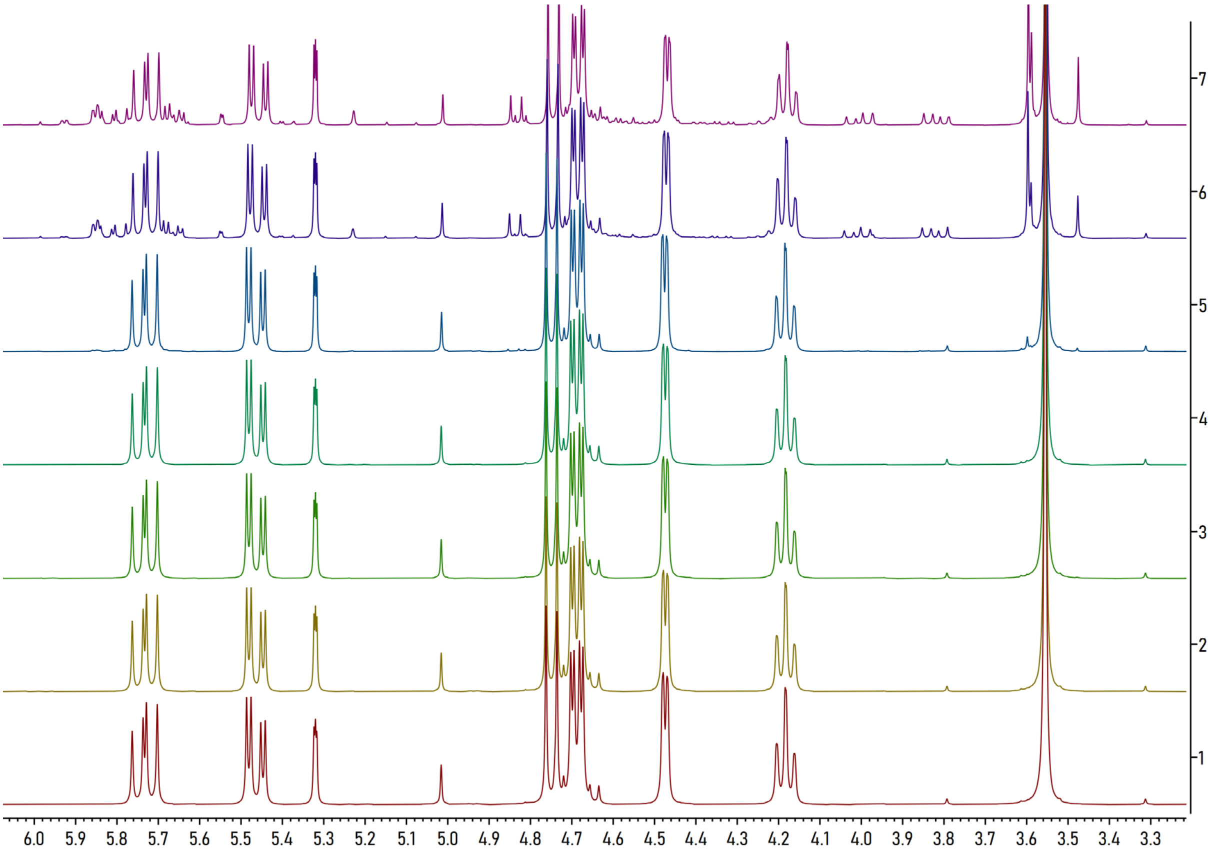

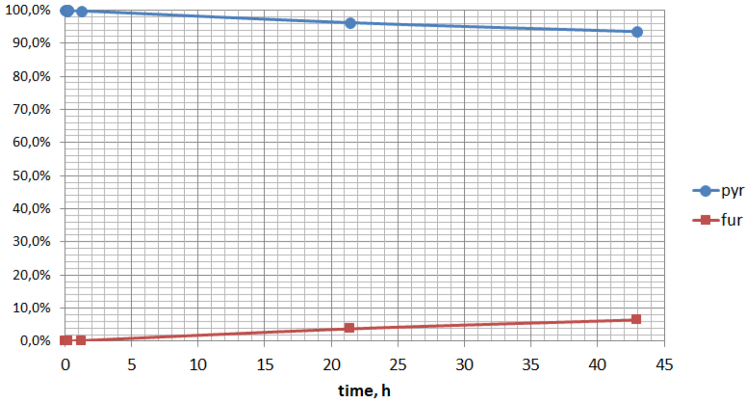

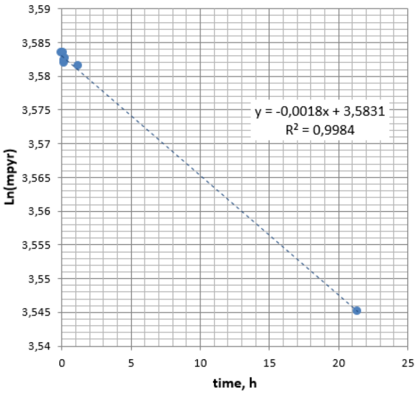

Integrated signals: H-3 (βpyr), H-1 (fur).

Methyl 2,3,6-tri-*O*-benzoyl-β-D-galactopyranoside 2p (140 µL TFA + 460 µL CD_2Cl_2)

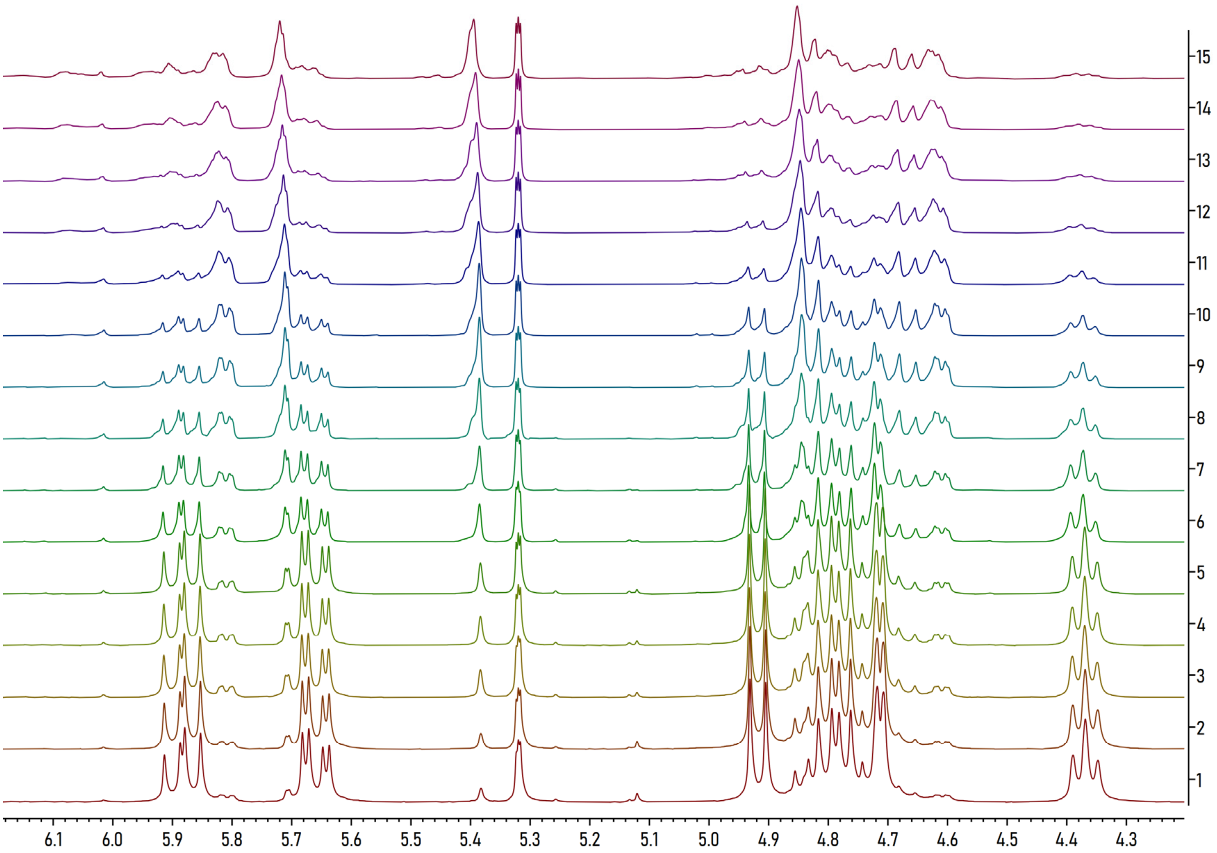

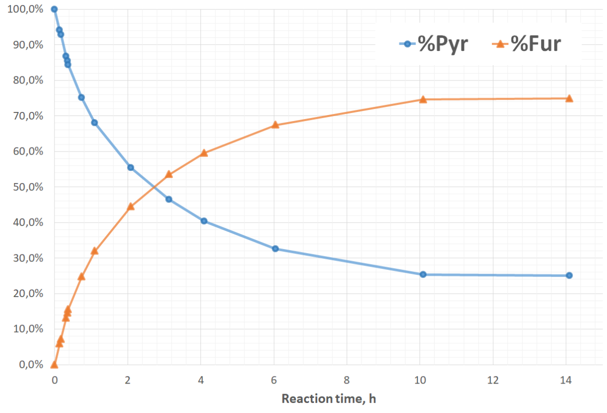

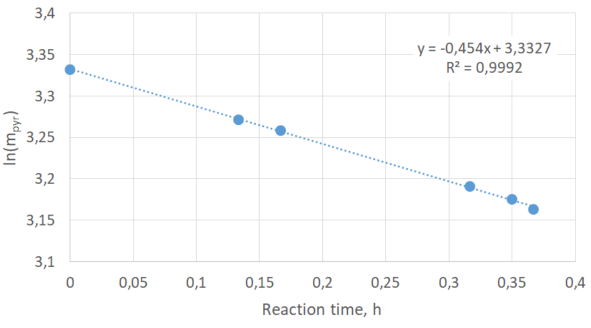

Integrated signals: H-3 (βpyr), H-1 (fur).

Copies of NMR spectra

Phenyl 2,3,6-tri-*O*-benzoyl-β-D-galactofuranoside

1H NMR (300.23 MHz), CD2Cl2, 298.0 K

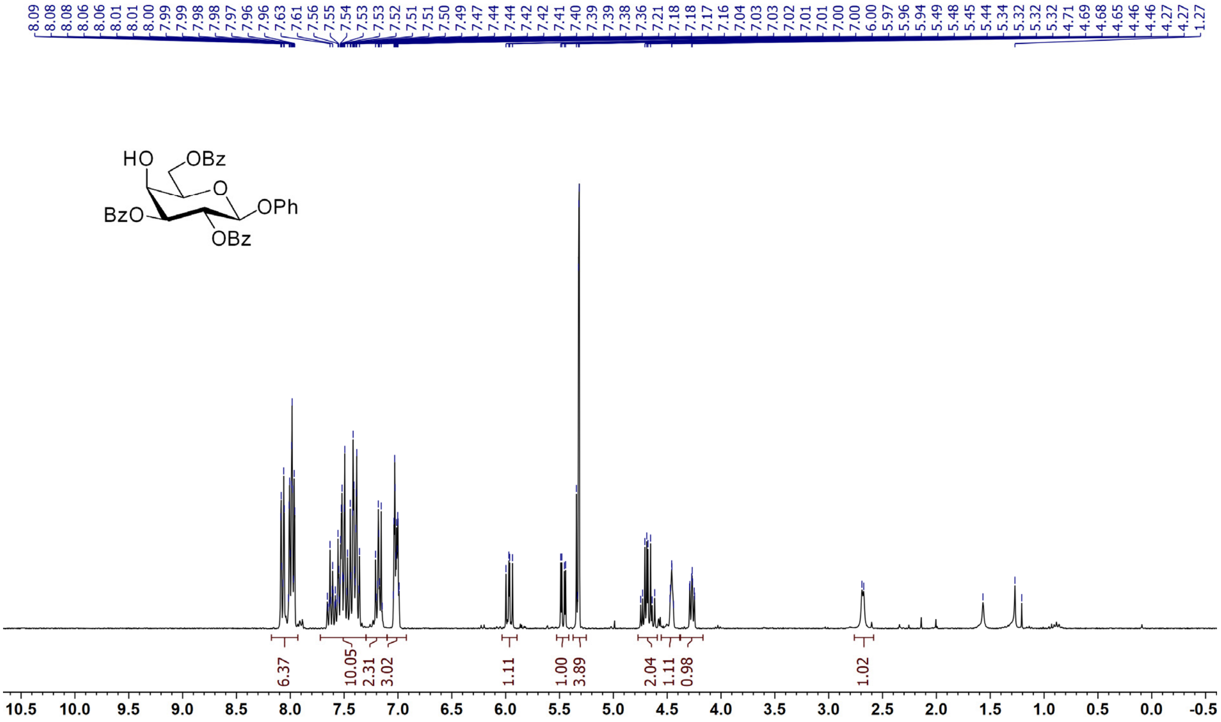

13C NMR (75.50 MHz), CD2Cl2, 298.1 K

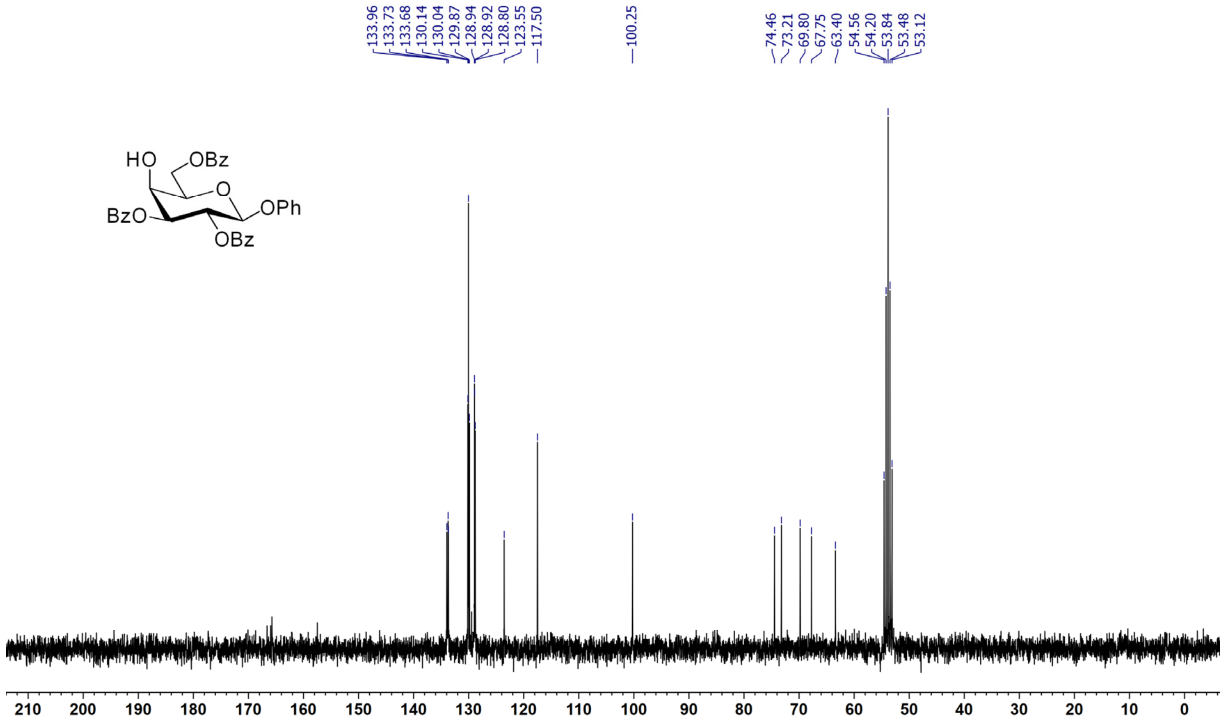

Phenyl 2,3,6-tri-*O*-benzoyl-β-D-galactofuranoside

1H NMR (300.13 MHz), CD2Cl2, 300.0 K

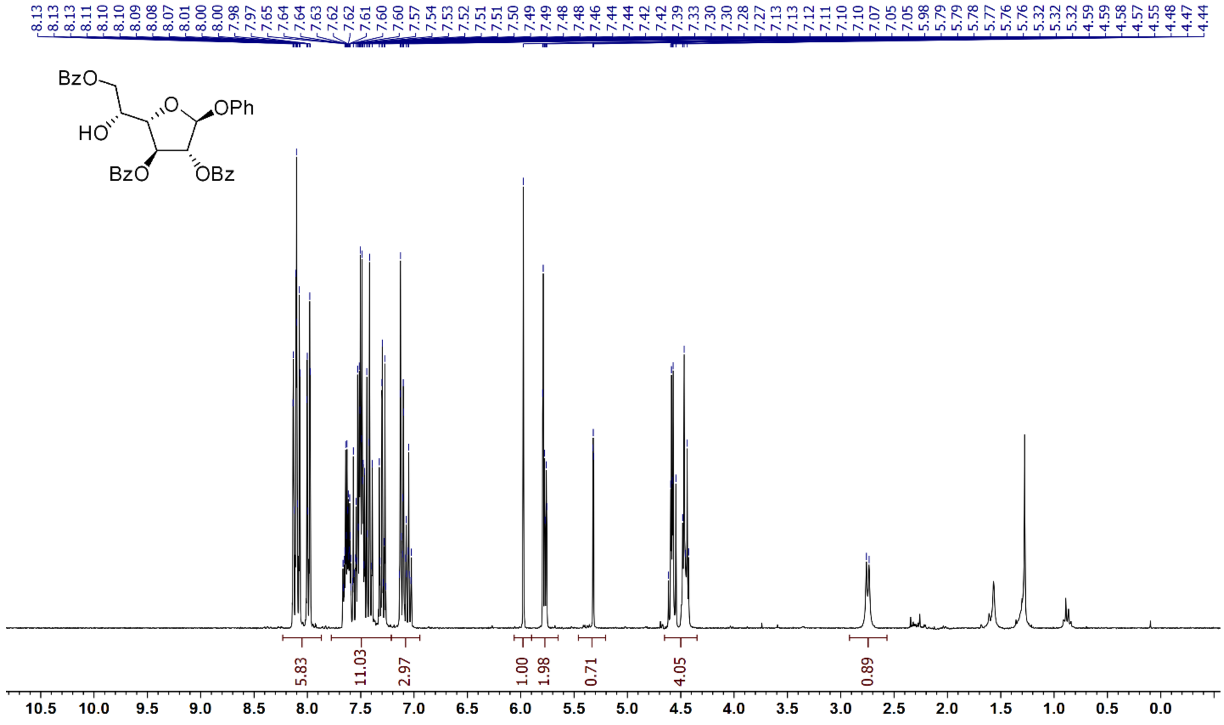

13C NMR (75.48 MHz), CD2Cl2, 300.1 K

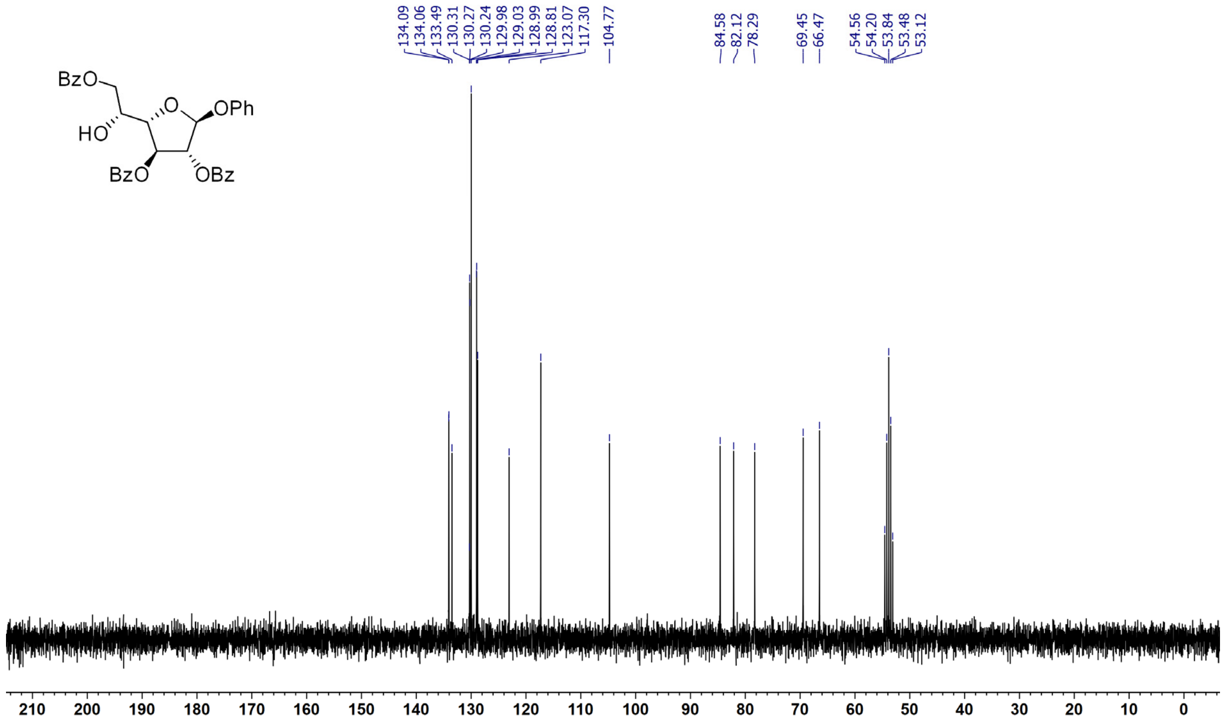

Ethyl 2,3,6-tri-*O*-benzoyl-1-thio-β-D-galactofuranoside

1H NMR (300.23 MHz), CD2Cl2, 298.0 K

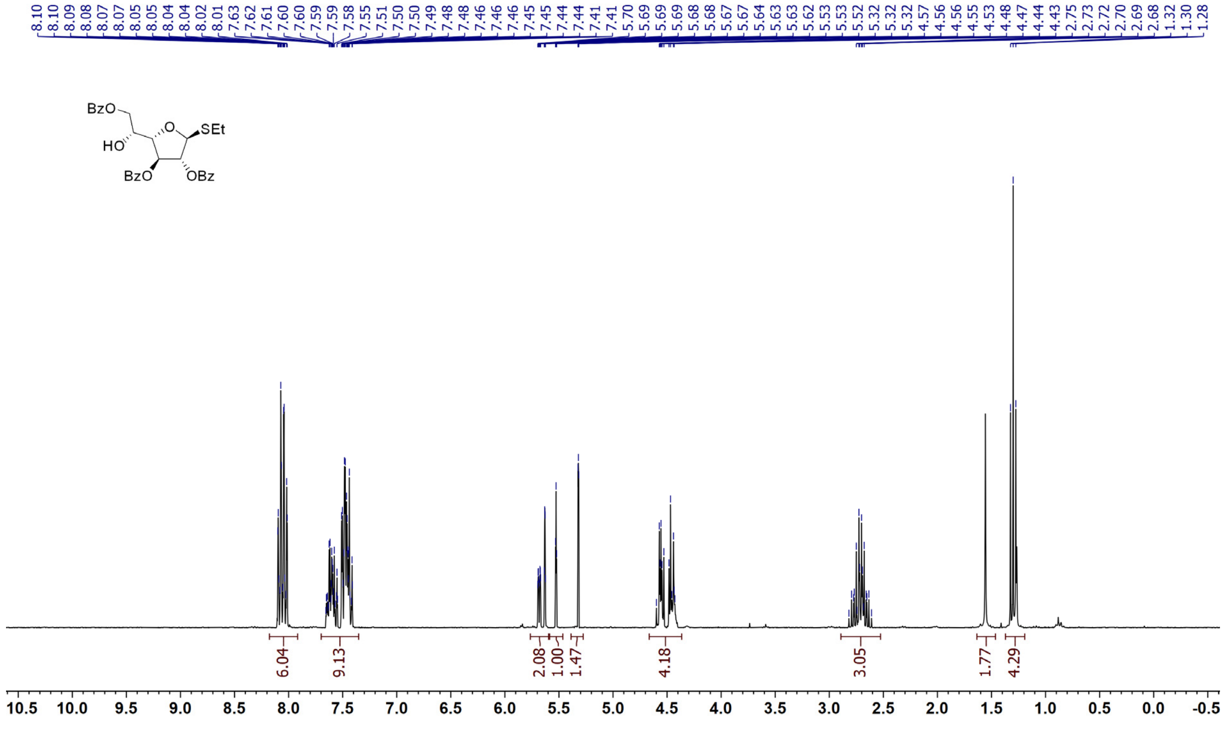

13C NMR (75.48 MHz), CD2Cl2, 300.1 K

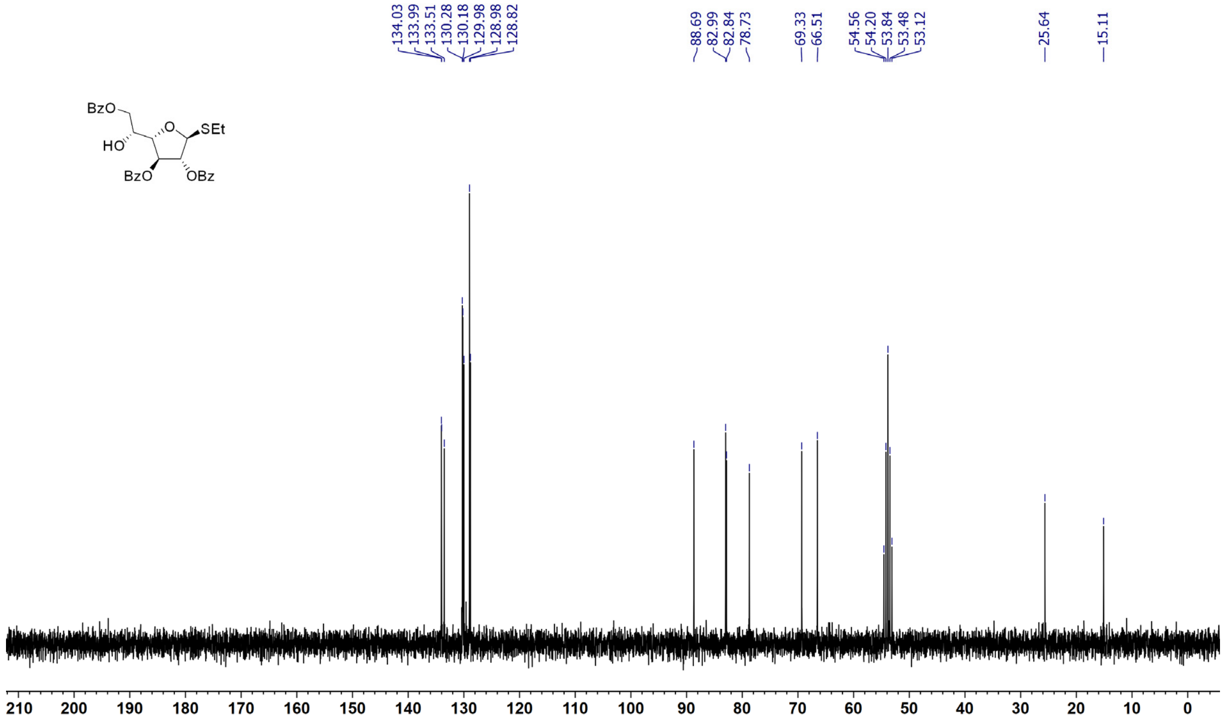

Ethyl 2,3,6-tri-*O*-benzoyl-1-thio-β-D-galactofuranoside (0.25 µL TfOH 24 h)

1H NMR (300.23 MHz), CD2Cl2, 298.0 K

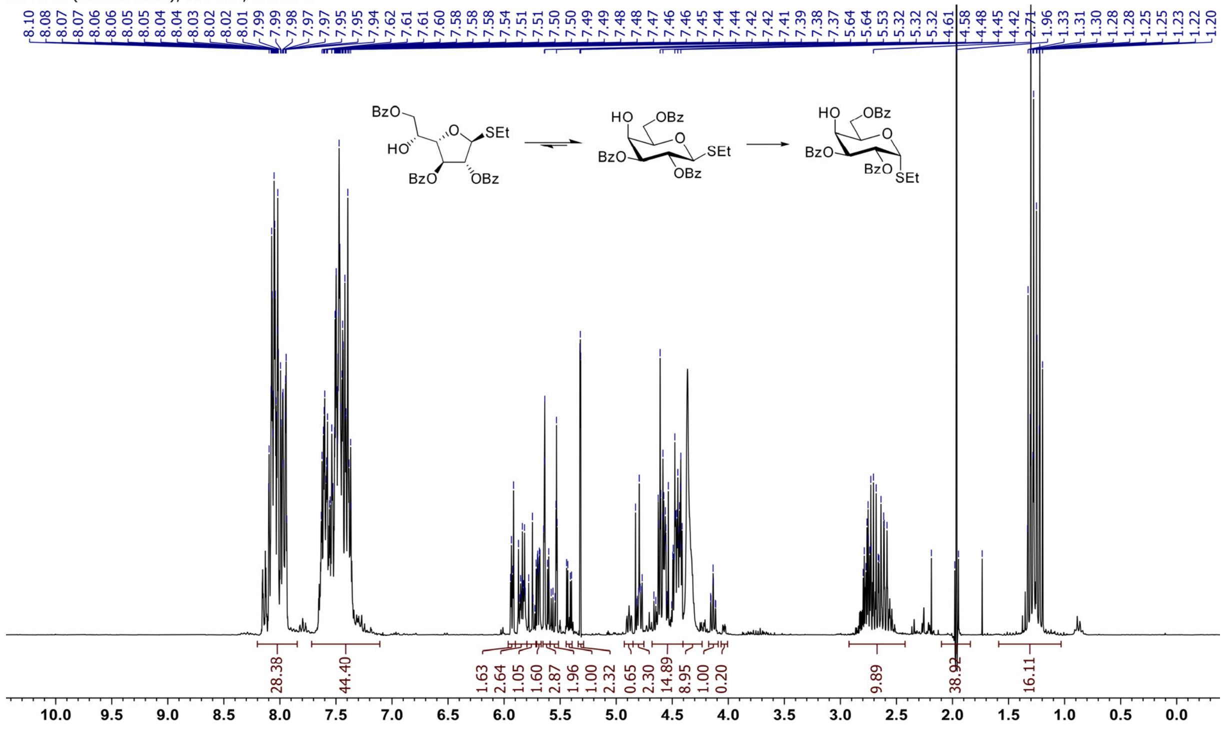

13C NMR (75.50 MHz), CD2Cl2, 298.0 K

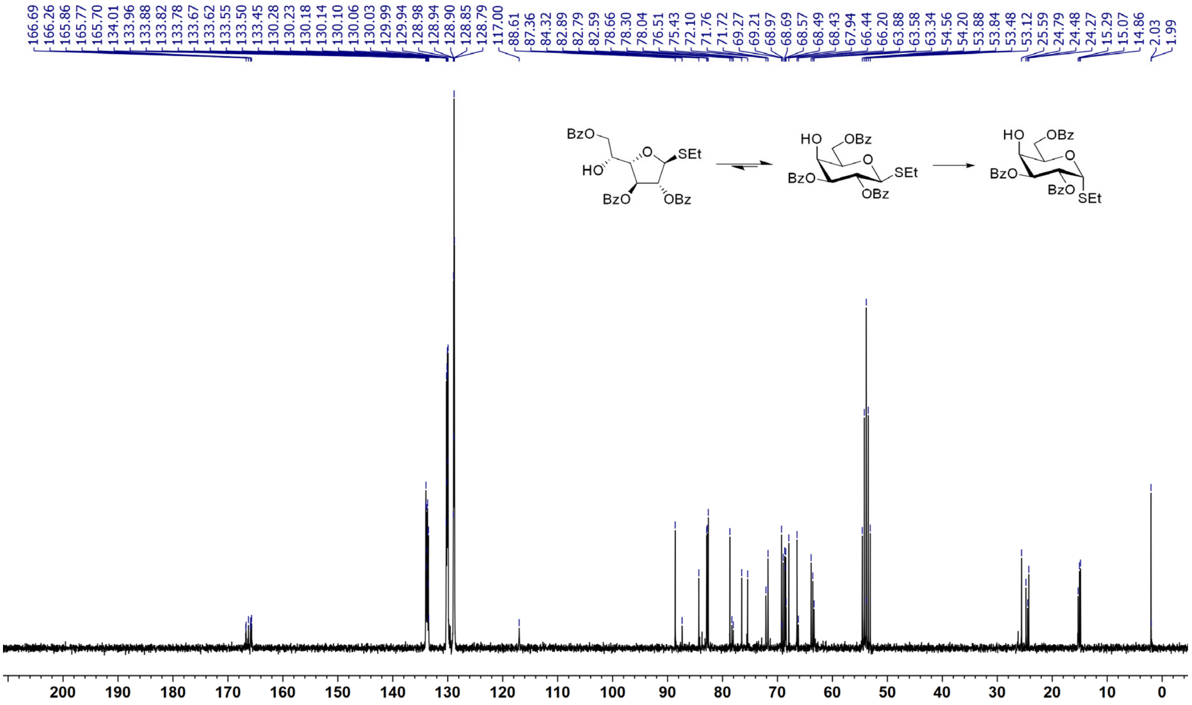

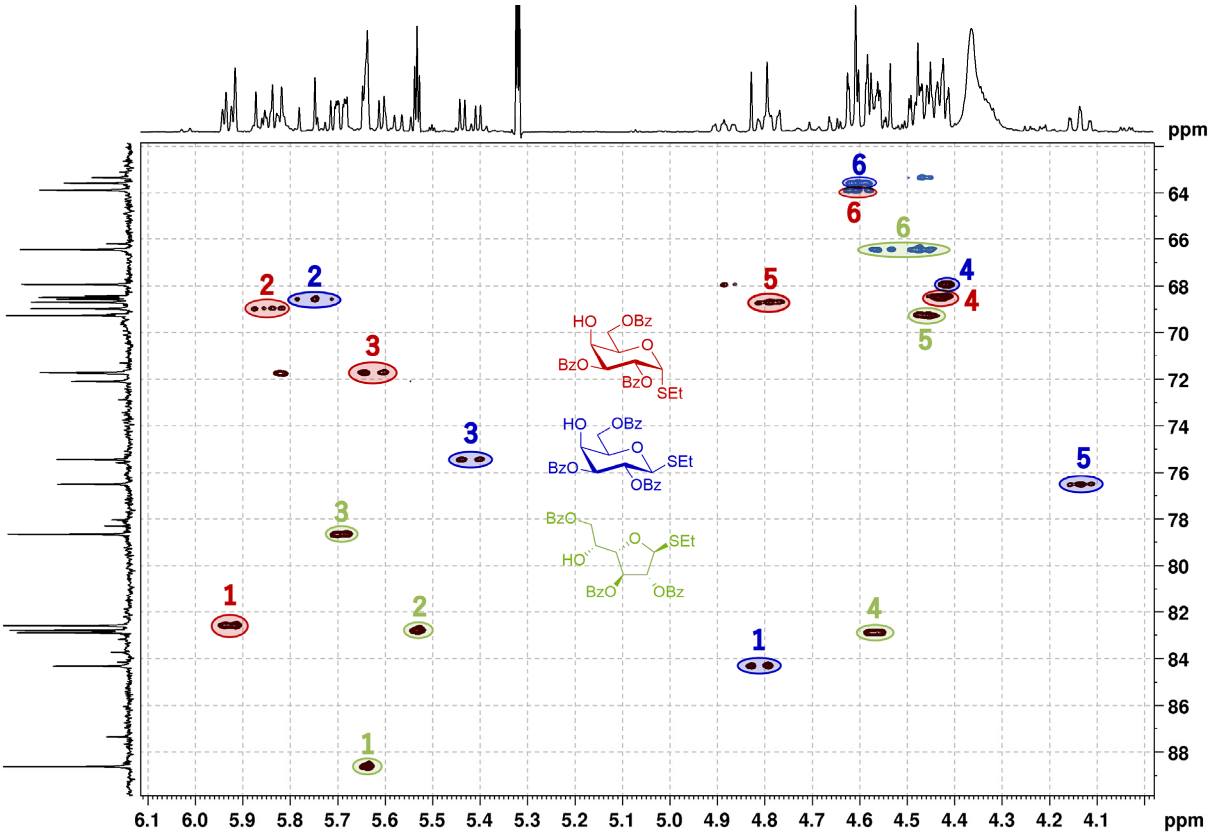

The structure of the third product was concluded on the basis of complete assigments of $^{1}H/^{13}C$ chemical shifts. Thus, downfield shift of H-1, $J_{1,2}$ = 5.7 Hz and upfield shift of C-1 relative to β-pyranoside already suggest α-pyranoside form of galactose. Same shifts and shapes of position 2 and 4 of new product and β-pyranoside, while significant downfield shift of H-3 and H-5 due to lack of H-1 shielding confirms isomeric structure of formed pyranoside (see HSQC and behold). The ^{13}C chemical shift of C-4 unambiguously indicates that it’s not a ring contraction product.

Cartesian coordinates (Å) and CCSDT energies (Eh) of the studied structures.

Structure **2p**, starting conformer, *gg* E= -2717.776143001039

O	1.79100	0.95110	0.03460
C	2.74120	0.74480	0.98460
O	2.47580	0.52050	2.14220
C	4.11040	0.78260	0.42300
C	4.34580	0.98920	-0.93860
C	5.64210	0.93640	-1.43120
C	6.70490	0.67230	-0.57260
C	6.47300	0.46570	0.78500
C	5.18060	0.52230	1.28270
H	3.51770	1.17090	-1.60720
H	5.82230	1.09110	-2.48710
H	7.71390	0.62180	-0.96200
H	7.29890	0.25290	1.45110
H	4.98430	0.34860	2.33140
O	1.23330	-1.74690	-0.04960
O	-2.04020	-0.27780	-0.75910
O	-3.12970	2.25150	-1.55050
C	-1.12880	-1.32880	-0.35250
C	0.28250	-0.79430	-0.53240
C	0.46520	0.47360	0.29760
C	-0.55750	1.54270	-0.07730
C	-1.94220	0.90470	0.05940
C	-3.14110	1.78500	-0.19790
H	0.45140	-0.60050	-1.58900
H	0.37420	0.24010	1.35770
H	-2.03740	0.59090	1.10560
C	2.30300	-2.02590	-0.83780
C	-4.19770	2.95360	-1.95930
O	-0.34910	2.02220	-1.39260
H	-4.05280	1.21690	-0.01410
H	-3.11590	2.63580	0.48510
O	-1.32140	-2.41350	-1.16460
H	-1.34460	-1.55940	0.69930
C	-2.49530	-3.18200	-0.86710
H	-0.49200	2.35980	0.65030
H	0.57540	2.29420	-1.46630
H	-2.49470	-3.47940	0.18460
H	-2.45320	-4.06340	-1.50070
H	-3.39900	-2.61250	-1.08970
O	2.34190	-1.76460	-2.01700

C	3.41670	-2.63270	-0.07180
C	3.32460	-2.88340	1.29940
C	4.42290	-3.38120	1.98640
C	5.61610	-3.62480	1.31280
C	5.71060	-3.37610	-0.05400
C	4.61440	-2.88400	-0.74540
H	2.40440	-2.67450	1.82430
H	4.35120	-3.57070	3.04960
H	6.47420	-4.00380	1.85360
H	6.64040	-3.55890	-0.57680
H	4.67790	-2.67260	-1.80360
C	-4.03880	3.45450	-3.34630
O	-5.16730	3.15100	-1.25680
C	-5.13090	4.07020	-3.96240
C	-5.01330	4.56120	-5.25410
C	-3.80260	4.44660	-5.93400
C	-2.71220	3.83720	-5.32110
C	-2.82690	3.33880	-4.03070
H	-6.06350	4.15520	-3.42130
H	-5.86210	5.03300	-5.73200
H	-3.71100	4.83070	-6.94220
H	-1.77360	3.74290	-5.85150
H	-1.98490	2.86020	-3.55230
H	-2.41060	-0.28230	-2.29200
C	-2.16370	-2.20680	-4.92870
S	-1.84850	-0.44480	-4.40420
O	-2.32070	0.34820	-5.49550
O	-2.83320	-0.31280	-3.22090
O	-0.48420	-0.37470	-3.97430
F	-1.75150	-3.06630	-4.00210
F	-3.46580	-2.39240	-5.14800
F	-1.49430	-2.43310	-6.06040

Structure **2p**, TS of the benzoate rotation, *gg* E= -2717.760606166427

O	1.94610	0.84720	-0.00270
C	2.76660	0.92640	1.07040
O	2.41630	0.65350	2.19570
C	4.13230	1.36400	0.69600
C	4.49030	1.62290	-0.62970
C	5.78950	2.00660	-0.93270
C	6.73520	2.13130	0.08100
C	6.38170	1.87160	1.40280
C	5.08470	1.48930	1.71050
H	3.75710	1.51950	-1.41620

H	6.06540	2.20630	-1.95990
H	7.74810	2.42900	-0.15890
H	7.11750	1.96660	2.19080
H	4.79790	1.28110	2.73190
O	1.54860	-1.81370	-0.12510
O	-1.78550	-0.40320	-1.04270
O	-2.96910	2.16320	-1.50480
C	-0.87030	-1.50380	-0.81310
C	0.56210	-0.95400	-0.73200
C	0.64060	0.27760	0.17380
C	-0.40490	1.34390	-0.12270
C	-1.76020	0.64950	-0.07030
C	-2.99210	1.50840	-0.23240
H	0.88920	-0.71560	-1.74510
H	0.53340	-0.04540	1.20830
H	-1.85010	0.20030	0.92640
C	1.75600	-3.13060	-0.32290
C	-4.10000	2.76370	-1.90810
O	-0.18920	1.94730	-1.38520
H	-3.88280	0.88540	-0.15630
H	-3.01790	2.25480	0.56350
O	-1.01240	-2.32200	-1.90270
H	-1.15610	-1.99170	0.12630
C	-2.01450	-3.33370	-1.76500
H	-0.36770	2.09070	0.67890
H	0.72480	2.26040	-1.40700
H	-1.76030	-3.99980	-0.93870
H	-2.01790	-3.89170	-2.69720
H	-2.99950	-2.89070	-1.60230
O	0.96780	-3.89360	-0.82480
C	3.08860	-3.53620	0.20550
C	4.06720	-2.60160	0.55250
C	5.30230	-3.02810	1.02130
C	5.56630	-4.38830	1.15180
C	4.59400	-5.32370	0.80620
C	3.36110	-4.90030	0.33100
H	3.86750	-1.54730	0.44390
H	6.05740	-2.29820	1.28350
H	6.52880	-4.71990	1.52040
H	4.79850	-6.38180	0.90690
H	2.59990	-5.61780	0.05660
C	-3.93140	3.43350	-3.22030
O	-5.12690	2.74550	-1.26270
C	-5.06850	3.89500	-3.88580
C	-4.94620	4.51150	-5.12230

C	-3.68730	4.67960	-5.69430
C	-2.55150	4.22890	-5.02830
C	-2.66990	3.60290	-3.79530
H	-6.03980	3.75680	-3.43060
H	-5.82950	4.85960	-5.64200
H	-3.59280	5.16060	-6.65970
H	-1.57360	4.35830	-5.47380
H	-1.79360	3.23850	-3.27970
H	-2.50330	-0.36190	-2.44110
C	-2.42200	-1.66210	-5.40220
S	-2.61510	0.01150	-4.60090
O	-3.65530	0.65590	-5.33910
O	-3.16970	-0.38100	-3.21300
O	-1.29990	0.57070	-4.51570
F	-1.43050	-2.34600	-4.83860
F	-3.55260	-2.36030	-5.29570
F	-2.14290	-1.47920	-6.69430

Structure **2p**, resulting conformar of the benzoate rotation, *gg* E= -2717.773763148762

O	1.81610	0.88070	0.16830
C	2.81170	0.51610	1.00910
O	2.64420	-0.22880	1.94740
C	4.11200	1.11730	0.63300
C	4.25250	1.94430	-0.48460
C	5.49900	2.46200	-0.80930
C	6.60780	2.15600	-0.02530
C	6.47110	1.32970	1.08740
C	5.22760	0.81160	1.41640
H	3.39290	2.17490	-1.09680
H	5.60650	3.10200	-1.67530
H	7.57900	2.55960	-0.28260
H	7.33380	1.08910	1.69490
H	5.10790	0.16440	2.27400
O	1.58540	-1.77750	-0.67480
O	-1.84110	-0.43950	-1.09670
O	-3.05110	2.10350	-1.46930
C	-0.90020	-1.50770	-0.82560
C	0.48620	-0.86390	-0.79950
C	0.54810	0.21600	0.27820
C	-0.54500	1.26820	0.06250
C	-1.88670	0.54350	-0.04720
C	-3.12120	1.39610	-0.22710
H	0.64440	-0.40620	-1.77470
H	0.45310	-0.22190	1.26960

H	-2.03400	0.01440	0.90240
C	1.66660	-2.63690	0.36290
C	-4.19430	2.59930	-1.96930
O	-0.29280	2.03380	-1.10150
H	-4.00470	0.75960	-0.21810
H	-3.19330	2.10930	0.59610
O	-0.95710	-2.41700	-1.84750
H	-1.17730	-1.94660	0.13450
C	-2.05620	-3.33510	-1.77210
H	-0.58260	1.91080	0.94950
H	0.59470	2.40730	-1.02020
H	-2.03440	-3.87040	-0.82000
H	-1.92670	-4.03440	-2.59370
H	-3.00890	-2.81450	-1.88210
O	0.74770	-2.86520	1.11700
C	3.01010	-3.25640	0.46010
C	4.10100	-2.76620	-0.26230
C	5.35330	-3.34280	-0.10110
C	5.52170	-4.41310	0.77270
C	4.43540	-4.90590	1.49150
C	3.18420	-4.32640	1.34040
H	3.97060	-1.92930	-0.93260
H	6.19870	-2.95490	-0.65430
H	6.49940	-4.86160	0.89590
H	4.56610	-5.73780	2.17150
H	2.33480	-4.69370	1.90000
C	-3.96690	3.32710	-3.24160
O	-5.27330	2.45380	-1.43500
C	-5.07430	3.69650	-4.00710
C	-4.89490	4.36570	-5.20880
C	-3.60990	4.67920	-5.64530
C	-2.50490	4.32060	-4.87920
C	-2.67910	3.64180	-3.68120
H	-6.06630	3.44670	-3.65590
H	-5.75390	4.64240	-5.80620
H	-3.47080	5.20160	-6.58340
H	-1.50650	4.56270	-5.21970
H	-1.82480	3.34900	-3.08870
H	-2.39670	-0.31470	-2.55090
C	-1.86910	-1.51460	-5.49380
S	-2.24730	0.12780	-4.69440
O	-3.20890	0.74160	-5.55500
O	-2.96220	-0.32290	-3.40090
O	-0.98500	0.74550	-4.41990
F	-0.96230	-2.18830	-4.79290

F	-2.97920	-2.24640	-5.59180
F	-1.39000	-1.28410	-6.71800

Structure **2p**, TS of the C1-O5 bond breaking, *gg*   E= -2717.750477839936

O	1.63030	0.81140	0.16200
C	2.57690	0.38570	1.03200
O	2.35030	-0.42570	1.90110
C	3.89660	1.00190	0.77560
C	4.10240	1.90780	-0.26820
C	5.36940	2.42940	-0.48990
C	6.43330	2.04860	0.32330
C	6.23100	1.14380	1.36260
C	4.96690	0.62160	1.58920
H	3.27800	2.19570	-0.90400
H	5.52830	3.13040	-1.29890
H	7.42090	2.45510	0.14550
H	7.05910	0.84500	1.99200
H	4.79720	-0.08830	2.38660
O	1.68500	-1.61130	-0.85510
O	-2.30400	-0.35340	-0.99920
O	-3.27370	2.24860	-1.51300
C	-0.69150	-1.99450	-0.64620
C	0.39130	-0.96860	-0.86260
C	0.35410	0.15860	0.18680
C	-0.67330	1.25190	-0.13510
C	-2.09950	0.70940	-0.07510
C	-3.19240	1.76000	-0.16690
H	0.29640	-0.56920	-1.87050
H	0.17420	-0.24350	1.18310
H	-2.21320	0.26350	0.91760
C	1.78890	-2.66020	-0.03480
C	-4.09250	3.28900	-1.73070
O	-0.42980	1.82310	-1.40670
H	-4.15000	1.31220	0.10170
H	-2.98460	2.58180	0.51880
O	-0.93820	-2.76630	-1.61790
H	-1.28540	-2.03710	0.25540
C	-2.01500	-3.74520	-1.51540
H	-0.57320	2.00550	0.65440
H	0.46300	2.19210	-1.41270
H	-2.39280	-3.77320	-0.49630
H	-1.58460	-4.69820	-1.80680
H	-2.78070	-3.43130	-2.21870
O	0.78430	-3.12600	0.49000

C	3.15450	-3.16110	0.15030
C	4.24960	-2.49740	-0.41180
C	5.53120	-2.97350	-0.18250
C	5.72340	-4.10830	0.60090
C	4.63280	-4.77200	1.15850
C	3.34870	-4.30040	0.93670
H	4.09760	-1.61350	-1.01350
H	6.38030	-2.45740	-0.61030
H	6.72590	-4.47600	0.77930
H	4.78620	-5.65330	1.76720
H	2.49380	-4.80190	1.36860
C	-4.11400	3.70720	-3.15470
O	-4.73300	3.81920	-0.84600
C	-4.88040	4.82010	-3.50960
C	-4.92420	5.24020	-4.83100
C	-4.20540	4.55050	-5.80510
C	-3.44280	3.44020	-5.45460
C	-3.39460	3.01710	-4.13310
H	-5.43420	5.34620	-2.74400
H	-5.51750	6.10370	-5.10330
H	-4.24120	4.87900	-6.83640
H	-2.88380	2.89840	-6.20610
H	-2.80960	2.15090	-3.86550
H	-2.22120	-0.05840	-1.93540
C	-1.47220	-1.80970	-5.56450
S	-1.06960	-0.25310	-4.62900
O	-1.06300	0.77110	-5.65370
O	-2.18290	-0.16150	-3.68460
O	0.21840	-0.54560	-4.02290
F	-1.53060	-2.86780	-4.73860
F	-2.65750	-1.70450	-6.18350
F	-0.53830	-2.06280	-6.49350

Structure **2p**, starting conformer, *gt* E= -2717.779985258681

O	-1.23680	0.20220	-4.20570
C	-1.30140	-0.29590	-5.46510
O	-0.44590	-1.00490	-5.94230
C	-2.53910	0.12410	-6.16180
C	-3.58190	0.76580	-5.48820
C	-4.73840	1.11080	-6.17400
C	-4.85800	0.82240	-7.53040
C	-3.82020	0.18280	-8.20390
C	-2.66520	-0.16920	-7.52180
H	-3.49070	0.98000	-4.43360

H	-5.54780	1.60210	-5.64980
H	-5.76110	1.09360	-8.06260
H	-3.91410	-0.04240	-9.25830
H	-1.85430	-0.67130	-8.03130
O	-1.54120	-2.27680	-2.94320
O	0.64910	-0.47680	-0.63930
O	3.00410	0.72550	0.12180
C	0.01680	-1.64710	-1.21920
C	-0.96870	-1.15550	-2.26920
C	-0.24320	-0.31680	-3.31320
C	0.51670	0.84420	-2.67880
C	1.42540	0.28260	-1.58830
C	2.16560	1.36920	-0.84820
H	-1.75010	-0.57890	-1.77900
H	0.45160	-0.94190	-3.87310
H	2.14710	-0.39870	-2.04920
C	-2.89170	-2.36370	-3.01100
C	3.83110	1.51800	0.82510
O	-0.35780	1.78960	-2.08460
H	2.77030	1.94060	-1.55220
H	1.48030	2.04700	-0.34300
O	-0.65390	-2.31600	-0.23160
H	0.81080	-2.25830	-1.66740
C	0.17270	-3.15940	0.58650
H	1.14020	1.31630	-3.44550
H	-0.96340	2.11560	-2.76310
H	0.66860	-3.91010	-0.03350
H	-0.49460	-3.64490	1.29370
H	0.91070	-2.56840	1.12790
O	-3.63640	-1.63090	-2.40230
C	-3.33110	-3.44200	-3.92730
C	-2.42880	-4.13890	-4.73510
C	-2.89200	-5.11870	-5.60260
C	-4.25160	-5.40980	-5.66530
C	-5.15290	-4.71710	-4.86070
C	-4.69540	-3.73380	-3.99620
H	-1.37520	-3.90570	-4.69070
H	-2.19260	-5.65380	-6.23170
H	-4.60950	-6.17500	-6.34260
H	-6.21020	-4.94290	-4.90990
H	-5.38480	-3.18480	-3.36970
C	4.55670	0.77690	1.88600
O	3.95130	2.70380	0.60370
C	5.56080	1.44260	2.59240
C	6.25230	0.78820	3.60140

C	5.94060	-0.53230	3.91500
C	4.93670	-1.19740	3.21690
C	4.24590	-0.54810	2.20270
H	5.79060	2.46920	2.34220
H	7.03240	1.30600	4.14450
H	6.47850	-1.04170	4.70460
H	4.69030	-2.22180	3.46470
H	3.46140	-1.06000	1.66490
H	-0.25370	0.21870	0.38260
C	0.15890	2.04490	2.98170
S	-0.56910	0.38930	2.53640
O	-1.77870	0.28480	3.29110
O	-0.92380	0.62210	1.05290
O	0.48520	-0.56770	2.69270
F	1.25220	2.28400	2.25600
F	-0.72750	3.01440	2.76330
F	0.48990	2.03550	4.27270

Structure **2p**, TS of the benzoate rotation, *gt* E= -2717.764129293760

O	-1.37160	0.21980	-4.20070
C	-1.28210	-0.16040	-5.49620
O	-0.38090	-0.84310	-5.92680
C	-2.41870	0.34170	-6.30420
C	-3.47170	1.06230	-5.73430
C	-4.52730	1.48880	-6.52860
C	-4.53810	1.19930	-7.89010
C	-3.49030	0.48080	-8.46050
C	-2.43380	0.05220	-7.67090
H	-3.46480	1.28110	-4.67660
H	-5.34310	2.04490	-6.08540
H	-5.36360	1.53220	-8.50650
H	-3.49930	0.25480	-9.51890
H	-1.61600	-0.51010	-8.10000
O	-1.77460	-2.18300	-3.00290
O	0.38560	-0.32940	-0.58510
O	2.92490	0.50760	0.07320
C	-0.42110	-1.47100	-0.98310
C	-1.26010	-1.09000	-2.21330
C	-0.43880	-0.33190	-3.25980
C	0.43260	0.78570	-2.70910
C	1.26980	0.19100	-1.58810
C	2.19330	1.19880	-0.94910
H	-2.10260	-0.48770	-1.87040

H	0.20320	-1.04500	-3.77500
H	1.86360	-0.63730	-1.98690
C	-2.31420	-3.34260	-2.58090
C	3.88420	1.20150	0.70830
O	-0.32740	1.86060	-2.18180
H	2.87540	1.59320	-1.70230
H	1.64250	2.02620	-0.50590
O	-1.23450	-1.72670	0.08880
H	0.26280	-2.29610	-1.21060
C	-0.68160	-2.62800	1.05530
H	1.09450	1.13060	-3.51040
H	-0.88680	2.21140	-2.88680
H	-0.54380	-3.61240	0.60470
H	-1.40600	-2.69110	1.86340
H	0.26320	-2.24840	1.44540
O	-2.27410	-3.75960	-1.44930
C	-2.94290	-4.07270	-3.71760
C	-3.14030	-3.48070	-4.96770
C	-3.74200	-4.20040	-5.99100
C	-4.14440	-5.51530	-5.77600
C	-3.94980	-6.10920	-4.53160
C	-3.35470	-5.39030	-3.50480
H	-2.83250	-2.46070	-5.13560
H	-3.89570	-3.73450	-6.95580
H	-4.61010	-6.07640	-6.57640
H	-4.26200	-7.13180	-4.36310
H	-3.20060	-5.84060	-2.53380
C	4.48900	0.43500	1.82570
O	4.20030	2.32800	0.39080
C	5.56000	1.00500	2.51740
C	6.14380	0.32670	3.57750
C	5.65890	-0.92290	3.95510
C	4.58960	-1.49300	3.27020
C	4.00420	-0.81870	2.20720
H	5.92450	1.97750	2.21630
H	6.97440	0.77100	4.11060
H	6.11310	-1.45110	4.78410
H	4.20950	-2.46200	3.56690
H	3.16820	-1.25500	1.68090
H	-0.30640	0.60530	0.40910
C	0.48740	2.69580	2.79270
S	-0.41980	1.08900	2.53990
O	-1.59160	1.18200	3.35460
O	-0.82010	1.22150	1.05620
O	0.54150	0.04750	2.74700

F	1.56740	2.75070	2.01310
F	-0.30510	3.72840	2.50970
F	0.86930	2.77410	4.06790

Structure **2p**, resulting conformar of the benzoate rotation, *gt* E= -2717.776104464550

O	0.92070	0.04700	2.25440
C	1.58110	-0.87930	2.98670
O	1.38230	-2.06810	2.88340
C	2.56860	-0.26570	3.90450
C	2.76900	1.11610	3.96320
C	3.72000	1.64060	4.82780
C	4.47530	0.79250	5.63270
C	4.28000	-0.58510	5.57370
C	3.32980	-1.11370	4.71270
H	2.18720	1.77290	3.33330
H	3.87400	2.71100	4.87220
H	5.21770	1.20480	6.30440
H	4.86940	-1.24460	6.19730
H	3.17030	-2.18140	4.65350
O	2.03950	-1.05940	-0.08010
O	-1.15920	0.40940	-1.23090
O	-3.91490	0.73270	-1.34180
C	-0.08710	-0.56750	-1.30630
C	0.83340	-0.28420	-0.11870
C	0.06210	-0.39960	1.19390
C	-1.17020	0.50810	1.19530
C	-1.98770	0.23800	-0.06300
C	-3.17980	1.15600	-0.18320
H	1.19220	0.73650	-0.23240
H	-0.23980	-1.42880	1.37730
H	-2.33100	-0.80060	-0.04610
C	2.00280	-2.40790	-0.07560
C	-4.99720	1.45930	-1.66850
O	-0.81700	1.88150	1.19860
H	-3.80150	1.06600	0.70810
H	-2.87980	2.19530	-0.29740
O	0.60330	-0.37620	-2.47250
H	-0.55400	-1.55280	-1.25790
C	-0.07350	-0.85060	-3.64470
H	-1.78040	0.26110	2.07050
H	-0.24860	2.05000	1.96110
H	-0.33890	-1.90410	-3.52570
H	0.62460	-0.73540	-4.46930
H	-0.97170	-0.26220	-3.83790

O	1.00190	-3.05130	-0.29910
C	3.33020	-2.99200	0.23230
C	4.37960	-2.21420	0.72820
C	5.59370	-2.80930	1.04220
C	5.76900	-4.17770	0.85670
C	4.72520	-4.95500	0.36070
C	3.50750	-4.36550	0.05400
H	4.23870	-1.15400	0.87930
H	6.40240	-2.20630	1.43420
H	6.71760	-4.63900	1.10070
H	4.86130	-6.01900	0.21670
H	2.68700	-4.95910	-0.32530
C	-5.64150	0.98440	-2.91880
O	-5.38600	2.39470	-1.00270
C	-6.82690	1.59910	-3.32780
C	-7.45750	1.18910	-4.49360
C	-6.90640	0.16500	-5.25940
C	-5.72350	-0.44860	-4.85690
C	-5.09060	-0.04310	-3.68950
H	-7.24370	2.39440	-2.72520
H	-8.37700	1.66670	-4.80620
H	-7.39800	-0.15400	-6.16980
H	-5.29360	-1.24280	-5.45350
H	-4.17110	-0.51650	-3.37700
H	-0.88540	1.82170	-1.70810
C	0.88700	3.46310	-3.83730
S	0.43590	3.55460	-2.03250
O	0.18740	4.93660	-1.76410
O	-0.90660	2.79770	-2.04570
O	1.45660	2.84050	-1.32400
F	1.00270	2.19390	-4.22420
F	-0.04960	4.05960	-4.57420
F	2.05200	4.08420	-4.02420

Structure **2p**, TS of the C1-O5 bond breaking, *gt* E= -2717.750215991204

O	0.78940	-0.06810	2.05810
C	1.36810	-1.09050	2.73170
O	1.18340	-2.25170	2.44430
C	2.25440	-0.62080	3.81820
C	2.45990	0.73760	4.07330
C	3.33280	1.12650	5.07990
C	4.00450	0.16590	5.83110
C	3.80270	-1.18850	5.57710
C	2.93050	-1.58200	4.57380

H	1.94480	1.48170	3.48350
H	3.49240	2.17860	5.27640
H	4.68730	0.47290	6.61320
H	4.32800	-1.93470	6.15890
H	2.77130	-2.62940	4.35860
O	2.32660	-0.71000	0.03180
O	-1.31260	0.41410	-1.59020
O	-4.09430	0.95920	-1.38310
C	0.51110	-0.97280	-1.54360
C	1.01020	-0.23500	-0.32830
C	0.05080	-0.38060	0.87030
C	-1.11620	0.61150	0.84220
C	-2.02990	0.38840	-0.35930
C	-3.17360	1.38300	-0.36240
H	1.16010	0.81030	-0.59140
H	-0.32790	-1.39980	0.93810
H	-2.44300	-0.61970	-0.27670
C	2.54860	-2.00320	-0.21690
C	-5.19710	1.70580	-1.54700
O	-0.65710	1.95180	0.81800
H	-3.67640	1.38890	0.60520
H	-2.82300	2.38970	-0.58420
O	1.04670	-0.67790	-2.65420
H	-0.30490	-1.67900	-1.50050
C	0.58960	-1.37290	-3.85520
H	-1.70250	0.42160	1.74820
H	-0.14460	2.12010	1.61910
H	1.13350	-2.31230	-3.91720
H	0.83960	-0.71790	-4.68160
H	-0.48370	-1.53360	-3.78600
O	1.74660	-2.64290	-0.88710
C	3.78550	-2.53580	0.36350
C	4.58210	-1.75500	1.20710
C	5.72280	-2.30250	1.77340
C	6.07270	-3.62190	1.49920
C	5.28140	-4.39950	0.65650
C	4.13760	-3.86050	0.08940
H	4.30390	-0.73430	1.42420
H	6.33620	-1.70270	2.43240
H	6.96330	-4.04660	1.94470
H	5.55630	-5.42450	0.44600
H	3.51100	-4.45470	-0.56130
C	-6.06630	1.20500	-2.64280
O	-5.44350	2.67770	-0.86440
C	-7.28010	1.85550	-2.87460

C	-8.12050	1.42100	-3.88950
C	-7.75250	0.33600	-4.68090
C	-6.54250	-0.31380	-4.45480
C	-5.69980	0.11660	-3.43860
H	-7.55330	2.69790	-2.25410
H	-9.06090	1.92730	-4.06490
H	-8.40800	-0.00210	-5.47360
H	-6.25530	-1.15580	-5.07130
H	-4.75890	-0.38470	-3.26350
H	-1.04200	1.33570	-1.83230
C	1.23290	3.19970	-4.25430
S	0.73630	3.38180	-2.47150
O	0.82190	4.80680	-2.23200
O	-0.62890	2.84890	-2.46980
O	1.70650	2.56000	-1.76900
F	1.16920	1.91340	-4.64000
F	0.42580	3.91030	-5.05510
F	2.49010	3.62560	-4.44580

Structure **2p**, starting conformer, *tg* E= -2717.776832317199

O	1.48450	1.21490	2.38930
C	2.27190	0.72120	3.37540
O	1.96670	-0.23350	4.05250
C	3.53750	1.48010	3.50400
C	3.92000	2.43870	2.56170
C	5.13250	3.09990	2.70190
C	5.96410	2.81210	3.78030
C	5.58510	1.85710	4.72070
C	4.37720	1.19000	4.58210
H	3.27800	2.65290	1.72000
H	5.43040	3.83740	1.96800
H	6.90900	3.32980	3.88680
H	6.23270	1.63250	5.55820
H	4.07410	0.44180	5.30150
O	1.76930	-1.00270	0.68840
O	-1.64090	0.11320	-0.01340
O	-3.75390	1.51420	2.54540
C	-0.50620	-0.79060	-0.05080
C	0.69110	-0.07180	0.55580
C	0.36230	0.43630	1.95280
C	-0.89440	1.29990	1.96940
C	-2.01940	0.49850	1.32180
C	-3.33010	1.25230	1.20080
H	0.98040	0.74540	-0.09990

H	0.22460	-0.40510	2.63120
H	-2.18340	-0.41010	1.90870
C	2.96670	-0.68520	0.14080
C	-4.90920	2.18800	2.69250
O	-0.73480	2.50480	1.24110
H	-3.20620	2.19060	0.66370
H	-4.06900	0.63760	0.68740
O	-0.23530	-1.09290	-1.35760
H	-0.78310	-1.67840	0.53270
C	-1.15030	-2.01610	-1.96380
H	-1.16430	1.50420	3.00990
H	0.02430	2.98410	1.59720
H	-1.21130	-2.93180	-1.37020
H	-0.75010	-2.23980	-2.94890
H	-2.14160	-1.57140	-2.06170
O	3.13440	0.26250	-0.59050
C	4.03350	-1.62410	0.56320
C	3.82510	-2.57910	1.56150
C	4.86500	-3.41420	1.94740
C	6.11170	-3.30440	1.33830
C	6.32180	-2.35360	0.34260
C	5.28770	-1.51320	-0.04190
H	2.85930	-2.65730	2.03840
H	4.70370	-4.14930	2.72520
H	6.92030	-3.95770	1.64090
H	7.29140	-2.26740	-0.13050
H	5.43900	-0.76720	-0.80980
C	-5.24810	2.42860	4.11700
O	-5.58160	2.54700	1.75080
C	-6.44560	3.08540	4.40930
C	-6.80010	3.33240	5.72760
C	-5.96040	2.92630	6.76160
C	-4.76570	2.27210	6.47460
C	-4.40780	2.02170	5.15680
H	-7.08830	3.39620	3.59720
H	-7.72920	3.84080	5.95010
H	-6.23710	3.11970	7.79030
H	-4.11270	1.95720	7.27830
H	-3.48060	1.51420	4.93310
H	-1.68090	1.20570	-1.10040
C	-0.78310	1.88350	-4.12160
S	-0.75650	2.68490	-2.44010
O	-1.13100	4.04830	-2.65210
O	-1.93240	1.94490	-1.76730
O	0.50580	2.34880	-1.85420

F	-0.58700	0.57040	-4.01410
F	-1.95490	2.10120	-4.71820
F	0.19030	2.41120	-4.86440

Structure **2p**, TS of the benzoate rotation, *tg* E= -2717.761714017956

O	1.55820	1.25790	2.24350
C	2.09120	0.96510	3.45170
O	1.61640	0.14450	4.20390
C	3.31720	1.75120	3.72660
C	3.86350	2.62560	2.78340
C	5.02640	3.32330	3.07980
C	5.64780	3.15310	4.31380
C	5.10560	2.28200	5.25540
C	3.94440	1.58210	4.96340
H	3.38280	2.75300	1.82440
H	5.44950	3.99860	2.34760
H	6.55500	3.69830	4.54140
H	5.58910	2.14910	6.21450
H	3.51430	0.90040	5.68410
O	1.90130	-0.93620	0.68470
O	-1.54650	0.22310	-0.16740
O	-3.66770	1.36280	2.50550
C	-0.38260	-0.63150	-0.35670
C	0.82170	-0.02770	0.38340
C	0.44990	0.47060	1.78070
C	-0.82510	1.29600	1.85590
C	-1.92440	0.48460	1.19110
C	-3.27080	1.18180	1.13940
H	1.21310	0.78490	-0.22790
H	0.33330	-0.39290	2.43460
H	-2.03100	-0.46830	1.71850
C	2.45950	-1.86100	-0.12000
C	-4.82120	2.02200	2.71710
O	-0.72090	2.53890	1.18410
H	-3.20030	2.14820	0.64370
H	-3.99740	0.56050	0.61650
O	-0.15670	-0.62310	-1.70670
H	-0.63640	-1.62340	0.03310
C	-0.92230	-1.57740	-2.44740
H	-1.07540	1.44210	2.91100
H	0.05340	3.00840	1.51970
H	-0.68710	-2.58820	-2.10790
H	-0.63060	-1.46340	-3.48800
H	-1.99220	-1.38540	-2.34440

O	1.97060	-2.28350	-1.13930
C	3.75720	-2.33010	0.44050
C	4.38590	-1.68040	1.50590
C	5.60520	-2.14460	1.98050
C	6.20140	-3.26000	1.39930
C	5.57810	-3.91010	0.33700
C	4.36230	-3.44540	-0.14330
H	3.92750	-0.81140	1.95240
H	6.09020	-1.63490	2.80300
H	7.15120	-3.62190	1.77240
H	6.04100	-4.77730	-0.11590
H	3.87050	-3.93980	-0.96980
C	-5.12390	2.18670	4.16070
O	-5.51880	2.42720	1.81330
C	-6.30890	2.83430	4.51720
C	-6.62950	3.01200	5.85520
C	-5.76860	2.54460	6.84490
C	-4.58630	1.89910	6.49390
C	-4.26190	1.71870	5.15600
H	-6.96830	3.19320	3.73890
H	-7.54880	3.51410	6.12760
H	-6.01900	2.68350	7.88900
H	-3.91680	1.53650	7.26310
H	-3.34420	1.21830	4.88290
H	-1.59370	1.37880	-1.16490
C	-0.64940	2.52850	-4.09380
S	-0.64380	3.01190	-2.29490
O	-1.01100	4.39410	-2.26710
O	-1.82750	2.16140	-1.79250
O	0.61330	2.57780	-1.76370
F	-0.41530	1.22450	-4.22880
F	-1.82720	2.81860	-4.64680
F	0.30850	3.21000	-4.72440

Structure **2p**, resulting conformar of the benzoate rotation, *tg*   E= -2717.774446127915

O	-0.38320	-1.75600	2.38980
C	0.10620	-3.01620	2.43760
O	0.26810	-3.70050	1.45300
C	0.42750	-3.43950	3.82030
C	0.27480	-2.58140	4.91270
C	0.61610	-3.01600	6.18620
C	1.11120	-4.30310	6.37540
C	1.26630	-5.15950	5.28820
C	0.92600	-4.73000	4.01420

H	-0.10180	-1.58000	4.76350
H	0.49870	-2.34980	7.03100
H	1.37840	-4.63820	7.36970
H	1.65380	-6.15930	5.43490
H	1.04620	-5.38250	3.16050
O	1.89430	-0.96820	0.94530
O	-0.73890	1.38370	-0.19320
O	-4.25670	0.59970	-0.18490
C	0.48790	0.62180	-0.37930
C	0.67000	-0.22210	0.88300
C	-0.53700	-1.12880	1.10700
C	-1.83520	-0.32250	1.13960
C	-1.90410	0.54140	-0.11490
C	-3.10980	1.46040	-0.16530
H	0.75400	0.47040	1.71760
H	-0.59980	-1.89130	0.33330
H	-1.91410	-0.11350	-0.99120
C	2.21780	-1.86080	-0.01330
C	-5.46140	1.19840	-0.16740
O	-1.90080	0.53660	2.26520
H	-3.15240	2.11200	0.70510
H	-3.08090	2.06670	-1.07030
O	1.53460	1.49540	-0.48880
H	0.35000	0.02060	-1.27940
C	1.62770	2.15900	-1.75670
H	-2.67740	-1.02040	1.13260
H	-1.80810	0.00250	3.06440
H	1.64520	1.42450	-2.56570
H	2.56030	2.71660	-1.74270
H	0.79100	2.84470	-1.89680
O	1.63690	-1.94660	-1.07210
C	3.36130	-2.71270	0.39250
C	3.82210	-2.74640	1.71110
C	4.86710	-3.59190	2.05750
C	5.46080	-4.39950	1.09170
C	5.00460	-4.36650	-0.22370
C	3.95440	-3.52980	-0.57210
H	3.35420	-2.12530	2.46090
H	5.21650	-3.62290	3.08130
H	6.27700	-5.05680	1.36420
H	5.46560	-4.99520	-0.97450
H	3.58510	-3.50090	-1.58810
C	-6.57990	0.22300	-0.15190
O	-5.59400	2.40260	-0.16410
C	-7.88640	0.71680	-0.15610

C	-8.96190	-0.15930	-0.13810
C	-8.73920	-1.53370	-0.11520
C	-7.43880	-2.03010	-0.11070
C	-6.35960	-1.15680	-0.12930
H	-8.04520	1.78630	-0.17300
H	-9.97280	0.22730	-0.14160
H	-9.57870	-2.21740	-0.10070
H	-7.26580	-3.09840	-0.09210
H	-5.35010	-1.54160	-0.12520
H	-0.66090	2.60950	0.72650
C	1.39750	4.89660	1.72700
S	0.12870	3.73890	2.44820
O	-0.59390	4.50890	3.41200
O	-0.77260	3.50750	1.21810
O	0.84030	2.55690	2.83480
F	2.05390	4.30620	0.73050
F	0.81020	6.00130	1.26840
F	2.26400	5.22840	2.68520

Structure **2p**, TS of the C1-O5 bond breaking, *tg* E= -2717.749440678476

O	1.55820	1.25790	2.24350
C	2.09120	0.96510	3.45170
O	1.61640	0.14450	4.20390
C	3.31720	1.75120	3.72660
C	3.86350	2.62560	2.78340
C	5.02640	3.32330	3.07980
C	5.64780	3.15310	4.31380
C	5.10560	2.28200	5.25540
C	3.94440	1.58210	4.96340
H	3.38280	2.75300	1.82440
H	5.44950	3.99860	2.34760
H	6.55500	3.69830	4.54140
H	5.58910	2.14910	6.21450
H	3.51430	0.90040	5.68410
O	1.90130	-0.93620	0.68470
O	-1.54650	0.22310	-0.16740
O	-3.66770	1.36280	2.50550
C	-0.38260	-0.63150	-0.35670
C	0.82170	-0.02770	0.38340
C	0.44990	0.47060	1.78070
C	-0.82510	1.29600	1.85590
C	-1.92440	0.48460	1.19110
C	-3.27080	1.18180	1.13940
H	1.21310	0.78490	-0.22790

H	0.33330	-0.39290	2.43460
H	-2.03100	-0.46830	1.71850
C	2.45950	-1.86100	-0.12000
C	-4.82120	2.02200	2.71710
O	-0.72090	2.53890	1.18410
H	-3.20030	2.14820	0.64370
H	-3.99740	0.56050	0.61650
O	-0.15670	-0.62310	-1.70670
H	-0.63640	-1.62340	0.03310
C	-0.92230	-1.57740	-2.44740
H	-1.07540	1.44210	2.91100
H	0.05340	3.00840	1.51970
H	-0.68710	-2.58820	-2.10790
H	-0.63060	-1.46340	-3.48800
H	-1.99220	-1.38540	-2.34440
O	1.97060	-2.28350	-1.13930
C	3.75720	-2.33010	0.44050
C	4.38590	-1.68040	1.50590
C	5.60520	-2.14460	1.98050
C	6.20140	-3.26000	1.39930
C	5.57810	-3.91010	0.33700
C	4.36230	-3.44540	-0.14330
H	3.92750	-0.81140	1.95240
H	6.09020	-1.63490	2.80300
H	7.15120	-3.62190	1.77240
H	6.04100	-4.77730	-0.11590
H	3.87050	-3.93980	-0.96980
C	-5.12390	2.18670	4.16070
O	-5.51880	2.42720	1.81330
C	-6.30890	2.83430	4.51720
C	-6.62950	3.01200	5.85520
C	-5.76860	2.54460	6.84490
C	-4.58630	1.89910	6.49390
C	-4.26190	1.71870	5.15600
H	-6.96830	3.19320	3.73890
H	-7.54880	3.51410	6.12760
H	-6.01900	2.68350	7.88900
H	-3.91680	1.53650	7.26310
H	-3.34420	1.21830	4.88290
H	-1.59370	1.37880	-1.16490
C	-0.64940	2.52850	-4.09380
S	-0.64380	3.01190	-2.29490
O	-1.01100	4.39410	-2.26710
O	-1.82750	2.16140	-1.79250
O	0.61330	2.57780	-1.76370

F	-0.41530	1.22450	-4.22880
F	-1.82720	2.81860	-4.64680
F	0.30850	3.21000	-4.72440

Structure **3p**, starting conformer, *gg*   E= -2909.154246691361

O	1.73140	0.95130	0.03680
C	2.66870	0.70380	0.99070
O	2.38680	0.44520	2.13720
C	4.04460	0.74330	0.44670
C	4.29910	0.99310	-0.90420
C	5.60070	0.94030	-1.38290
C	6.64950	0.63320	-0.52130
C	6.39840	0.38340	0.82560
C	5.10090	0.44000	1.30960
H	3.48180	1.20860	-1.57590
H	5.79580	1.12890	-2.43050
H	7.66250	0.58290	-0.90020
H	7.21340	0.13730	1.49370
H	4.88960	0.23340	2.34940
O	1.15620	-1.74350	-0.12320
O	-2.09580	-0.24660	-0.82770
O	-3.15900	2.29090	-1.60150
C	-1.19540	-1.29590	-0.42530
C	0.21940	-0.77170	-0.59070
C	0.40010	0.47590	0.27050
C	-0.61390	1.55860	-0.09070
C	-2.00330	0.92400	0.01240
C	-3.19490	1.81110	-0.25420
H	0.39650	-0.55530	-1.64170
H	0.29400	0.21700	1.32340
H	-2.11770	0.59350	1.05130
C	2.23110	-2.01220	-0.90950
C	-4.21230	3.01040	-2.01830
O	-0.38650	2.07210	-1.38930
H	-4.11100	1.24370	-0.09130
H	-3.17800	2.65460	0.43800
O	-1.40160	-2.37420	-1.26560
H	-1.41340	-1.55180	0.61680
C	-2.37520	-3.28850	-0.86360
H	-0.55540	2.35560	0.65950
H	0.53990	2.34210	-1.44400
C	-2.07200	-4.21190	0.12820
C	-3.60930	-3.28700	-1.49660
O	2.28550	-1.71230	-2.07860

C	3.32640	-2.66040	-0.15210
C	3.21310	-2.95820	1.20810
C	4.29500	-3.49740	1.88980
C	5.49270	-3.73530	1.22220
C	5.60830	-3.43920	-0.13360
C	4.52850	-2.90580	-0.82010
H	2.28950	-2.75420	1.72890
H	4.20700	-3.72400	2.94440
H	6.33790	-4.14700	1.75920
H	6.54170	-3.61780	-0.65130
H	4.60830	-2.65790	-1.86920
C	-4.02090	3.53340	-3.39290
O	-5.19280	3.20610	-1.33080
C	-5.09620	4.16420	-4.02280
C	-4.94640	4.67840	-5.30220
C	-3.72040	4.57190	-5.95540
C	-2.64670	3.94740	-5.32840
C	-2.79350	3.42580	-4.05060
H	-6.04080	4.24300	-3.50210
H	-5.78220	5.16230	-5.79100
H	-3.60380	4.97450	-6.95370
H	-1.69600	3.86000	-5.83800
H	-1.96410	2.93720	-3.56020
H	-2.40380	-0.21780	-2.40190
C	-1.99250	-2.09480	-5.04200
S	-1.79020	-0.31720	-4.50710
O	-2.29450	0.45100	-5.60160
O	-2.79990	-0.23480	-3.33780
O	-0.43820	-0.17240	-4.05850
F	-1.48170	-2.92810	-4.14220
F	-3.28340	-2.37690	-5.21980
F	-1.34870	-2.25620	-6.19950
C	-4.56000	-4.23280	-1.12460
C	-4.27480	-5.16180	-0.12870
C	-3.03120	-5.14920	0.49660
H	-1.09600	-4.19450	0.59610
H	-3.81780	-2.55770	-2.26640
H	-5.52550	-4.23910	-1.61400
H	-5.01780	-5.89500	0.15760
H	-2.80240	-5.87340	1.26800

Structure **3p**, TS of the benzoate rotation, *gg* E= -2909.138710330449

O	1.87500	0.85950	0.03780
C	2.81520	0.84900	1.01180

O	2.57880	0.54800	2.15890
C	4.15210	1.22860	0.49710
C	4.37030	1.52600	-0.85080
C	5.64690	1.84990	-1.28930
C	6.70870	1.87570	-0.38970
C	6.49440	1.57800	0.95380
C	5.22050	1.25570	1.39700
H	3.54710	1.49870	-1.54960
H	5.81430	2.07940	-2.33360
H	7.70370	2.12620	-0.73560
H	7.32030	1.59640	1.65290
H	5.04070	1.01860	2.43640
O	1.38140	-1.77330	-0.06310
O	-1.93210	-0.19560	-0.74910
O	-3.12180	2.38330	-1.14060
C	-1.10120	-1.34660	-0.49450
C	0.36130	-0.88540	-0.55710
C	0.56980	0.34540	0.33650
C	-0.44710	1.46580	0.14360
C	-1.82700	0.83420	0.24470
C	-3.02970	1.74940	0.14080
H	0.59470	-0.65170	-1.59620
H	0.54440	0.02200	1.37620
H	-1.90140	0.36950	1.23550
C	1.47120	-3.11230	-0.18190
C	-4.20070	2.14140	-1.91040
O	-0.30130	2.10320	-1.10860
H	-3.93490	1.17750	0.32930
H	-2.93480	2.53950	0.88610
O	-1.39400	-2.22200	-1.52300
H	-1.35760	-1.74760	0.48970
C	-2.52120	-3.01990	-1.36220
H	-0.32310	2.18020	0.96600
H	0.63400	2.30800	-1.23810
C	-2.63810	-3.89440	-0.28770
C	-3.50220	-2.95580	-2.34240
O	0.57000	-3.84370	-0.50930
C	2.83480	-3.58020	0.19120
C	3.91350	-2.70220	0.32080
C	5.16950	-3.18790	0.65790
C	5.35530	-4.55050	0.87220
C	4.28320	-5.42980	0.74130
C	3.02810	-4.94790	0.39800
H	3.77350	-1.64700	0.14640
H	6.00170	-2.50230	0.75260

H	6.33450	-4.92780	1.13860
H	4.42680	-6.48970	0.90690
H	2.18880	-5.62140	0.29160
C	-4.13330	2.87560	-3.19850
O	-5.10890	1.40840	-1.58500
C	-5.17670	2.70870	-4.11080
C	-5.14270	3.36230	-5.33380
C	-4.06590	4.18550	-5.65280
C	-3.02380	4.35360	-4.74620
C	-3.05410	3.70160	-3.52140
H	-6.00410	2.06200	-3.85320
H	-5.95190	3.22810	-6.03990
H	-4.03790	4.69240	-6.60910
H	-2.18360	4.98840	-4.99630
H	-2.24110	3.82110	-2.82080
H	-1.95880	0.11940	-2.38980
C	-0.62290	-1.13070	-4.93090
S	-1.39570	0.51400	-4.50070
O	-2.22800	0.84020	-5.61520
O	-2.33280	0.13870	-3.32690
O	-0.31470	1.36080	-4.10330
F	0.24430	-1.50640	-3.99350
F	-1.55750	-2.06880	-5.06750
F	0.02550	-0.99120	-6.08890
C	-4.61940	-3.77860	-2.24350
C	-4.75690	-4.65100	-1.16870
C	-3.76470	-4.70460	-0.19410
H	-1.85090	-3.95540	0.45000
H	-3.38830	-2.26400	-3.16410
H	-5.38630	-3.72870	-3.00600
H	-5.62920	-5.28720	-1.09190
H	-3.85890	-5.38850	0.64000

Structure **3p**, resulting conformar of the benzoate rotation, *gg*   E= -2909.152498914363

O	1.81350	0.89950	0.16570
C	2.83780	0.49630	0.95350
O	2.69150	-0.24320	1.89940
C	4.13680	1.05400	0.51240
C	4.24980	1.87200	-0.61500
C	5.49430	2.35380	-0.99730
C	6.62800	2.02190	-0.26080
C	6.51840	1.20450	0.86140
C	5.27720	0.72140	1.24760

H	3.37050	2.12390	-1.18970
H	5.58060	2.98690	-1.87080
H	7.59720	2.39840	-0.56250
H	7.40020	0.94420	1.43230
H	5.17780	0.08260	2.11420
O	1.37630	-1.77170	-0.60370
O	-1.96230	-0.21930	-0.89620
O	-3.12060	2.36410	-1.24870
C	-1.08900	-1.33250	-0.62040
C	0.33690	-0.78810	-0.68770
C	0.52180	0.30590	0.36280
C	-0.52450	1.41640	0.20110
C	-1.90730	0.77090	0.14980
C	-3.10060	1.69340	0.01730
H	0.47270	-0.35870	-1.67870
H	0.46380	-0.11300	1.36500
H	-2.04730	0.25360	1.10650
C	1.45700	-2.61210	0.44980
C	-4.23380	2.28330	-2.00410
O	-0.29820	2.17520	-0.96980
H	-4.01480	1.11700	0.13520
H	-3.04840	2.45410	0.79710
O	-1.27230	-2.27790	-1.61250
H	-1.34500	-1.72110	0.36360
C	-2.30990	-3.18870	-1.41760
H	-0.48380	2.05320	1.09240
H	0.61840	2.48040	-0.95560
C	-2.23190	-4.12620	-0.39520
C	-3.38000	-3.16870	-2.29960
O	0.57370	-2.74490	1.26720
C	2.74550	-3.34280	0.47440
C	3.82180	-2.96470	-0.33220
C	5.02590	-3.64950	-0.24410
C	5.15930	-4.71600	0.64040
C	4.08710	-5.09640	1.44370
C	2.88490	-4.40900	1.36540
H	3.71870	-2.13140	-1.01170
H	5.86150	-3.34940	-0.86310
H	6.09930	-5.24930	0.70600
H	4.19090	-5.92550	2.13150
H	2.04720	-4.68920	1.98930
C	-4.09630	3.04120	-3.27220
O	-5.22040	1.65790	-1.68260
C	-5.14340	2.98270	-4.19410
C	-5.04820	3.66310	-5.39870

C	-3.90770	4.40780	-5.68850
C	-2.86320	4.47010	-4.77130
C	-2.95370	3.78900	-3.56530
H	-6.02110	2.39680	-3.95840
H	-5.85930	3.61130	-6.11350
H	-3.83230	4.93620	-6.63050
H	-1.97470	5.04480	-4.99850
H	-2.14010	3.82620	-2.85650
H	-2.13560	0.06020	-2.47640
C	-0.76800	-1.07800	-5.05300
S	-1.60470	0.52060	-4.57430
O	-2.44340	0.85280	-5.68220
O	-2.52450	0.07040	-3.41400
O	-0.55930	1.39400	-4.13950
F	0.12400	-1.43670	-4.13340
F	-1.66530	-2.05100	-5.20090
F	-0.13930	-0.88610	-6.21430
C	-4.39570	-4.10830	-2.15150
C	-4.33910	-5.04900	-1.12800
C	-3.25720	-5.05490	-0.25210
H	-1.37940	-4.12780	0.27120
H	-3.41160	-2.42660	-3.08460
H	-5.23430	-4.09890	-2.83610
H	-5.13230	-5.77670	-1.01490
H	-3.20380	-5.78930	0.54150

Structure **3p**, TS of the C1-O5 bond breaking, *gg* E= -2909.122602884333

O	1.75960	0.89350	0.35270
C	2.70500	0.31990	1.13640
O	2.44410	-0.52460	1.96290
C	4.06320	0.82370	0.84140
C	4.30140	1.77860	-0.15070
C	5.60040	2.18980	-0.41510
C	6.66350	1.64980	0.30340
C	6.42860	0.69620	1.29090
C	5.13270	0.28370	1.56030
H	3.47640	2.19000	-0.71370
H	5.78440	2.92880	-1.18400
H	7.67580	1.97040	0.09210
H	7.25580	0.27400	1.84650
H	4.93600	-0.46250	2.31730
O	1.53370	-1.45640	-0.80950
O	-2.26720	0.07710	-0.63960

O	-3.08360	2.72260	-1.25020
C	-0.85940	-1.63670	-0.42930
C	0.30560	-0.71410	-0.68640
C	0.43120	0.36390	0.41110
C	-0.50210	1.55960	0.17500
C	-1.96730	1.14350	0.25700
C	-2.97500	2.28000	0.10970
H	0.17610	-0.24630	-1.65820
H	0.25460	-0.06600	1.39630
H	-2.11820	0.73600	1.26040
C	1.60670	-2.56600	-0.06680
C	-4.17970	2.37150	-1.95450
O	-0.24810	2.16250	-1.07890
H	-3.94870	1.94330	0.45600
H	-2.65150	3.14060	0.69380
O	-1.26340	-2.33530	-1.41670
H	-1.36160	-1.69720	0.52370
C	-2.40320	-3.17280	-1.27740
H	-0.31340	2.26590	0.99130
H	0.66840	2.46720	-1.09730
C	-2.47290	-4.09600	-0.25120
C	-3.38450	-3.01820	-2.23750
O	0.60800	-2.98240	0.50550
C	2.93370	-3.18700	-0.02720
C	4.03700	-2.57270	-0.62830
C	5.28550	-3.16840	-0.53940
C	5.43600	-4.37330	0.14170
C	4.33670	-4.98760	0.73780
C	3.08590	-4.39640	0.65720
H	3.91790	-1.63520	-1.15090
H	6.14190	-2.69150	-0.99730
H	6.41280	-4.83500	0.20970
H	4.45770	-5.92410	1.26590
H	2.22500	-4.85900	1.11970
C	-4.15130	2.91140	-3.33590
O	-5.07990	1.69870	-1.50150
C	-5.22610	2.62180	-4.17900
C	-5.23760	3.09870	-5.48110
C	-4.17490	3.86710	-5.94940
C	-3.10160	4.15630	-5.11280
C	-3.08650	3.68080	-3.80890
H	-6.04230	2.01970	-3.80430
H	-6.07070	2.86900	-6.13290
H	-4.18130	4.23520	-6.96770
H	-2.27080	4.74530	-5.47920

H	-2.24810	3.89270	-3.16270
H	-2.18450	0.34060	-1.58650
C	-0.56410	-1.21210	-4.87790
S	-1.52700	0.35380	-4.57430
O	-2.42750	0.41170	-5.70770
O	-2.19090	0.08230	-3.29840
O	-0.49390	1.36910	-4.52360
F	0.35990	-1.41160	-3.92120
F	-1.36960	-2.28560	-4.89970
F	0.07470	-1.15130	-6.05600
C	-4.50770	-3.83260	-2.15140
C	-4.61830	-4.77120	-1.12880
C	-3.60540	-4.90270	-0.18450
H	-1.66790	-4.19570	0.46320
H	-3.27140	-2.27020	-3.00900
H	-5.29720	-3.72810	-2.88380
H	-5.49460	-5.40340	-1.06990
H	-3.68740	-5.63750	0.60530

Structure **3p**, starting conformer, *gt* E= -2909.159871918963

O	2.24760	1.59180	-0.68530
C	3.56020	1.58020	-0.34630
O	3.94940	1.47650	0.79380
C	4.43790	1.69610	-1.53390
C	3.93050	1.63870	-2.83470
C	4.79480	1.72330	-3.91770
C	6.16350	1.86750	-3.70930
C	6.67170	1.92360	-2.41380
C	5.81280	1.83550	-1.32850
H	2.86930	1.51680	-2.99430
H	4.40120	1.67360	-4.92460
H	6.83480	1.93340	-4.55620
H	7.73610	2.03400	-2.25210
H	6.19460	1.87260	-0.31750
O	1.75660	-1.06890	0.13890
O	-1.41690	0.58890	0.79380
O	-3.31250	2.19820	1.73260
C	-0.43310	-0.45430	0.89810
C	0.71150	-0.11110	-0.04090
C	1.27590	1.26070	0.31480
C	0.19710	2.34290	0.33830
C	-0.92880	1.86580	1.25160

C	-2.10490	2.83530	1.29120
H	0.35150	-0.13440	-1.06650
H	1.76320	1.21730	1.28820
H	-0.53150	1.72300	2.25730
C	2.26470	-1.67980	-0.95840
C	-3.36820	1.74650	2.99770
O	-0.34160	2.58400	-0.95020
H	-1.85170	3.67230	1.94130
H	-2.34080	3.19950	0.29650
O	-1.02340	-1.64200	0.50630
H	-0.10030	-0.49750	1.94100
C	-1.77480	-2.28400	1.49380
H	0.63000	3.25680	0.75970
H	0.38490	2.78310	-1.55500
C	-1.14650	-3.21120	2.31330
C	-3.12830	-2.00720	1.62000
O	1.80380	-1.54500	-2.06760
C	3.45000	-2.50730	-0.63160
C	4.01880	-2.51550	0.64460
C	5.14460	-3.28830	0.89440
C	5.70490	-4.05580	-0.12270
C	5.13960	-4.04940	-1.39550
C	4.01720	-3.27600	-1.65080
H	3.58650	-1.91420	1.43070
H	5.58670	-3.29060	1.88230
H	6.58280	-4.65770	0.07560
H	5.57550	-4.64550	-2.18680
H	3.57060	-3.25810	-2.63540
C	-4.59950	0.96200	3.26130
O	-2.49640	1.95880	3.81350
C	-4.80200	0.45870	4.54760
C	-5.92900	-0.29930	4.82890
C	-6.85950	-0.56020	3.82670
C	-6.65940	-0.06330	2.54190
C	-5.53320	0.69630	2.25660
H	-4.06870	0.66340	5.31550
H	-6.08130	-0.69000	5.82670
H	-7.73730	-1.15520	4.04540
H	-7.37880	-0.27210	1.76080
H	-5.37130	1.07430	1.25780
H	-2.36230	0.59340	-0.41850
C	-3.76730	-1.35850	-2.55740
S	-2.80900	0.23910	-2.54130
O	-3.47430	1.09820	-3.47060
O	-3.10850	0.73320	-1.11020

O	-1.43350	-0.11690	-2.71980
F	-3.23820	-2.22710	-1.69880
F	-5.04020	-1.13910	-2.22750
F	-3.71770	-1.87370	-3.78660
C	-3.86430	-2.66870	2.59700
C	-3.24980	-3.59840	3.42980
C	-1.89210	-3.87040	3.28530
H	-0.08960	-3.40680	2.18530
H	-3.59490	-1.28380	0.96810
H	-4.91700	-2.44760	2.70960
H	-3.82700	-4.10980	4.18940
H	-1.41040	-4.59580	3.92850

Structure **3p**, TS of the benzoate rotation, *gt* E= -2909.146673827417

O	2.28390	1.49720	-0.69900
C	3.57130	1.68800	-0.32530
O	3.94020	1.64540	0.82580
C	4.45760	1.93550	-1.48660
C	3.97920	1.92890	-2.79960
C	4.85400	2.14850	-3.85490
C	6.20520	2.37300	-3.60690
C	6.68480	2.37830	-2.29940
C	5.81460	2.16040	-1.24180
H	2.93140	1.74930	-2.99110
H	4.48240	2.14260	-4.87130
H	6.88500	2.54250	-4.43240
H	7.73570	2.55130	-2.10670
H	6.17380	2.15920	-0.22200
O	1.91650	-1.09490	0.02840
O	-1.34990	0.53270	0.77530
O	-3.08390	2.07690	2.12510
C	-0.47860	-0.61080	0.70300
C	0.76240	-0.24490	-0.12390
C	1.33110	1.11460	0.30260
C	0.30770	2.23170	0.45940
C	-0.78660	1.71030	1.37520
C	-1.91220	2.71150	1.59220
H	0.47550	-0.23410	-1.17530
H	1.84700	0.98700	1.25340
H	-0.35100	1.42680	2.33510
C	1.94870	-2.44030	0.10600
C	-3.03790	1.62380	3.39080
O	-0.28220	2.61050	-0.77230

H	-1.56280	3.50030	2.25730
H	-2.23810	3.14080	0.65010
O	-1.22060	-1.57330	0.04760
H	-0.20610	-0.90800	1.71970
C	-2.14150	-2.30450	0.76960
H	0.80430	3.08460	0.93490
H	0.42200	2.82060	-1.39920
C	-2.56680	-1.96950	2.05080
C	-2.66310	-3.41810	0.11840
O	0.98800	-3.14690	0.28850
C	3.34330	-2.94460	-0.02720
C	4.40490	-2.11780	-0.40310
C	5.68410	-2.64280	-0.52750
C	5.91240	-3.99210	-0.27420
C	4.85700	-4.81970	0.10080
C	3.57660	-4.29920	0.22160
H	4.22930	-1.07270	-0.60460
H	6.50210	-1.99830	-0.82260
H	6.91110	-4.39920	-0.37000
H	5.03320	-5.86920	0.29800
H	2.74890	-4.93220	0.51040
C	-4.23080	0.81620	3.74390
O	-2.10730	1.84460	4.13670
C	-4.32080	0.29770	5.03750
C	-5.40190	-0.49420	5.39570
C	-6.39830	-0.77340	4.46390
C	-6.31110	-0.25980	3.17340
C	-5.23090	0.53220	2.81070
H	-3.53710	0.51630	5.74960
H	-5.46700	-0.89700	6.39810
H	-7.23960	-1.39530	4.74230
H	-7.08150	-0.48320	2.44700
H	-5.15410	0.91920	1.80540
H	-2.31850	0.72360	-0.39150
C	-3.94810	-0.48530	-3.01570
S	-2.69860	0.81350	-2.54840
O	-3.07610	1.98920	-3.27030
O	-3.05770	0.98150	-1.05700
O	-1.40760	0.22140	-2.73550
F	-3.73330	-1.60490	-2.32810
F	-5.18370	-0.05150	-2.76760
F	-3.82930	-0.74260	-4.31910
C	-3.61870	-4.19790	0.75380
C	-4.05410	-3.87510	2.03780
C	-3.52310	-2.76320	2.67820

H	-2.18780	-1.09590	2.55730
H	-2.31340	-3.65620	-0.87700
H	-4.02310	-5.06300	0.24350
H	-4.80310	-4.48220	2.52920
H	-3.86200	-2.48890	3.66860

Structure **3p**, resulting conformar of the benzoate rotation, *gt* E= -2909.155295112350

O	1.03660	0.28960	2.36780
C	1.62830	-0.71940	3.04980
O	1.24900	-1.86630	2.99210
C	2.78680	-0.25290	3.84540
C	3.18600	1.08620	3.86080
C	4.29390	1.46850	4.60470
C	5.00880	0.51980	5.33040
C	4.61500	-0.81600	5.31380
C	3.50710	-1.20220	4.57440
H	2.63530	1.82020	3.29090
H	4.60190	2.50600	4.61610
H	5.87410	0.82090	5.90720
H	5.17290	-1.55390	5.87540
H	3.19260	-2.23640	4.54820
O	1.80050	-0.98840	-0.01590
O	-1.21540	0.90410	-0.95290
O	-3.81720	1.78660	-1.00580
C	-0.36360	-0.24810	-1.05230
C	0.70950	-0.06090	0.01620
C	0.03890	-0.03870	1.39140
C	-1.05430	1.03620	1.47340
C	-1.98010	0.90030	0.26920
C	-3.01080	2.00480	0.16360
H	1.18660	0.90020	-0.16460
H	-0.38940	-1.01280	1.61970
H	-2.49180	-0.06390	0.33750
C	1.58520	-2.32100	0.00770
C	-4.81520	0.88550	-0.91730
O	-0.51280	2.34310	1.47180
H	-3.62650	2.02660	1.06070
H	-2.53790	2.97080	0.01360
O	0.20550	-0.25640	-2.31300
H	-0.97660	-1.13010	-0.87530
C	-0.60720	-0.73650	-3.34080
H	-1.64000	0.85770	2.38180
H	0.12520	2.41190	2.19380

C	-1.09540	-2.03690	-3.31150
C	-0.87140	0.10780	-4.40910
O	0.49540	-2.82420	-0.14820
C	2.83820	-3.07560	0.24370
C	4.00900	-2.44330	0.66980
C	5.14820	-3.19480	0.92260
C	5.12740	-4.57520	0.74430
C	3.96240	-5.20750	0.31710
C	2.81890	-4.46130	0.07230
H	4.02070	-1.37310	0.81600
H	6.05140	-2.70410	1.26150
H	6.01800	-5.15880	0.94040
H	3.94630	-6.28070	0.17860
H	1.90530	-4.94060	-0.25170
C	-5.37430	0.53700	-2.24580
O	-5.19040	0.41300	0.13410
C	-6.60510	-0.12140	-2.30150
C	-7.13690	-0.49640	-3.52640
C	-6.43560	-0.22980	-4.70020
C	-5.20390	0.41530	-4.64700
C	-4.67360	0.80440	-3.42450
H	-7.13480	-0.33120	-1.38220
H	-8.09430	-0.99940	-3.56790
H	-6.84780	-0.52970	-5.65550
H	-4.65160	0.60850	-5.55670
H	-3.71120	1.29300	-3.38010
H	-0.72590	2.20290	-1.72590
C	2.00190	3.04780	-2.96240
S	0.55250	3.94180	-2.20060
O	0.30610	5.06820	-3.04530
O	-0.59880	2.93350	-2.42400
O	0.87640	4.10710	-0.81740
F	2.31930	1.97230	-2.24430
F	1.71850	2.67450	-4.20940
F	3.04510	3.87820	-2.98250
C	-1.64620	-0.35790	-5.46600
C	-2.15690	-1.65130	-5.44620
C	-1.87880	-2.48720	-4.36910
H	-0.85970	-2.68770	-2.47980
H	-0.48000	1.11490	-4.40220
H	-1.86070	0.29920	-6.29910
H	-2.76920	-2.00650	-6.26480
H	-2.26520	-3.49830	-4.35140

Structure **3p**, TS of the C1-O5 bond breaking, *gt*   E= -2909.124430493293

O	-3.88190	0.02180	1.39190
C	-4.19750	-0.21540	2.68790
O	-3.36550	-0.47950	3.52630
C	-5.64770	-0.08620	2.94630
C	-6.55780	0.22130	1.93130
C	-7.90580	0.35990	2.23130
C	-8.34970	0.19760	3.54080
C	-7.44420	-0.10550	4.55450
C	-6.09690	-0.24790	4.25940
H	-6.21080	0.35410	0.91690
H	-8.61010	0.59730	1.44470
H	-9.40160	0.30940	3.77170
H	-7.78940	-0.22800	5.57290
H	-5.38180	-0.47600	5.03740
O	-2.67860	2.18430	2.25010
O	-0.40470	0.75340	-0.85950
O	0.52090	-1.02730	-2.71130
C	-0.53650	1.66670	1.27910
C	-2.03490	1.54100	1.12340
C	-2.49540	0.06760	1.03710
C	-2.41040	-0.52550	-0.37610
C	-0.97170	-0.54600	-0.87530
C	-0.83420	-1.17980	-2.25510
H	-2.36320	2.10510	0.25580
H	-1.92440	-0.54760	1.73180
H	-0.38750	-1.13330	-0.16430
C	-1.94930	2.15900	3.35980
C	1.46850	-1.78190	-2.13050
O	-3.19820	0.21450	-1.29280
H	-1.11670	-2.23130	-2.22510
H	-1.43620	-0.65210	-2.98960
O	-0.02070	2.80760	0.99820
H	0.10090	0.80930	1.42930
C	1.39170	2.96880	0.99190
H	-2.76920	-1.55770	-0.30040
H	-4.12740	0.12620	-1.04570
C	2.20140	2.06990	0.32070
C	1.87540	4.09090	1.63710
O	-0.77670	1.78130	3.29400
C	-2.62340	2.59750	4.57850
C	-3.98980	2.89860	4.57280
C	-4.61110	3.27650	5.75210
C	-3.87580	3.35760	6.93200

C	-2.51460	3.06070	6.93740
C	-1.88550	2.67840	5.76380
H	-4.55780	2.82750	3.65690
H	-5.66880	3.50340	5.75340
H	-4.36540	3.65200	7.85160
H	-1.94790	3.12530	7.85670
H	-0.83090	2.44030	5.75410
C	2.83680	-1.27960	-2.40920
O	1.21840	-2.74860	-1.44040
C	3.92640	-2.09500	-2.09420
C	5.21960	-1.63410	-2.29290
C	5.43160	-0.35180	-2.79460
C	4.34800	0.46530	-3.10180
C	3.05100	0.00480	-2.91600
H	3.74860	-3.08450	-1.69520
H	6.06220	-2.26990	-2.05330
H	6.44140	0.01050	-2.94160
H	4.51100	1.46600	-3.48080
H	2.21310	0.64960	-3.13660
H	-0.78310	1.34180	-1.55990
C	-0.44960	2.51390	-5.09490
S	-0.19640	3.24590	-3.40460
O	-0.41600	4.66350	-3.60520
O	-1.24100	2.58190	-2.61710
O	1.15660	2.85150	-3.06060
F	-0.29730	1.18030	-5.06880
F	-1.68200	2.78000	-5.55440
F	0.43680	3.01470	-5.96900
C	3.24830	4.31160	1.62360
C	4.09470	3.42200	0.96750
C	3.57160	2.30920	0.31690
H	1.77500	1.23310	-0.21190
H	1.19410	4.76610	2.13650
H	3.65280	5.18050	2.12550
H	5.16160	3.60200	0.95590
H	4.22310	1.62690	-0.21300

Structure **3p**, starting conformer, *tg* E= -2909.154023071750

O	1.72370	1.18640	2.65180
C	2.50600	0.66460	3.62620
O	2.18260	-0.28760	4.29860
C	3.78900	1.39470	3.75450
C	4.17890	2.36890	2.83150

C	5.40170	3.00970	2.97820
C	6.23630	2.68600	4.04420
C	5.84990	1.71550	4.96530
C	4.63160	1.06890	4.82010
H	3.53340	2.61280	2.00060
H	5.70480	3.76010	2.25970
H	7.18880	3.18850	4.15620
H	6.49930	1.46350	5.79360
H	4.32170	0.31050	5.52580
O	1.73450	-0.99210	0.82970
O	-1.65140	0.27590	0.48150
O	-3.43450	1.93770	3.12800
C	-0.57100	-0.65930	0.35930
C	0.70890	0.00170	0.84990
C	0.53050	0.48630	2.27880
C	-0.66370	1.43000	2.39150
C	-1.89340	0.72060	1.82940
C	-3.13150	1.59440	1.76990
H	0.96910	0.82470	0.18910
H	0.38810	-0.36320	2.94630
H	-2.09810	-0.15600	2.45220
C	2.87920	-0.72990	0.15540
C	-4.50060	2.73460	3.32410
O	-0.45800	2.63110	1.66900
H	-2.95010	2.49530	1.18680
H	-3.96130	1.04060	1.33100
O	-0.44930	-0.96370	-0.98720
H	-0.80970	-1.54530	0.95470
C	-0.90420	-2.19930	-1.43020
H	-0.84650	1.63750	3.44990
H	0.31160	3.08160	2.03980
C	-0.45100	-3.37920	-0.85190
C	-1.76490	-2.21520	-2.51880
O	3.08660	0.31210	-0.42150
C	3.82950	-1.86510	0.22280
C	3.50510	-3.06560	0.86040
C	4.42650	-4.10330	0.89120
C	5.67290	-3.94850	0.29110
C	5.99970	-2.75240	-0.34280
C	5.08120	-1.71380	-0.37850
H	2.53800	-3.18350	1.32670
H	4.17270	-5.03320	1.38340
H	6.38970	-4.75950	0.31760
H	6.96920	-2.63150	-0.80840
H	5.32190	-0.78040	-0.86840

C	-4.72300	3.04120	4.75910
O	-5.18920	3.14180	2.41450
C	-5.81480	3.84190	5.10210
C	-6.05980	4.15250	6.43180
C	-5.21580	3.66600	7.42680
C	-4.12620	2.86820	7.08930
C	-3.87780	2.55440	5.75990
H	-6.46240	4.21350	4.31990
H	-6.90730	4.77270	6.69350
H	-5.40720	3.90850	8.46450
H	-3.46980	2.49050	7.86260
H	-3.03260	1.93500	5.49720
H	-2.28940	0.83510	-0.85300
C	-0.86440	2.10760	-3.20700
S	-2.48220	2.42930	-2.33450
O	-3.44880	2.66350	-3.36010
O	-2.78100	1.04070	-1.71780
O	-2.20450	3.41440	-1.33390
F	0.09430	1.84370	-2.32150
F	-0.98580	1.07760	-4.04270
F	-0.53060	3.19560	-3.90070
C	-2.18730	-3.43750	-3.03060
C	-1.75630	-4.62860	-2.45440
C	-0.88950	-4.59500	-1.36630
H	0.24590	-3.34880	-0.02500
H	-2.09420	-1.28020	-2.94990
H	-2.85950	-3.45540	-3.87900
H	-2.08960	-5.57760	-2.85390
H	-0.54000	-5.51750	-0.92030

Structure **3p**, TS of the benzoate rotation, *tg* E= -2909.141573527540

O	1.89160	1.45170	2.33180
C	2.44920	1.50480	3.55970
O	2.01040	0.90100	4.51250
C	3.63510	2.39320	3.59750
C	4.14180	3.00150	2.44630
C	5.26180	3.81760	2.53030
C	5.88000	4.03100	3.75910
C	5.37720	3.42600	4.90820
C	4.25890	2.60920	4.82830
H	3.66420	2.83070	1.49270
H	5.65400	4.28600	1.63690
H	6.75350	4.66770	3.82130

H	5.85770	3.59120	5.86380
H	3.85940	2.13200	5.71260
O	1.81800	-1.20650	0.60660
O	-1.49980	0.17140	0.45160
O	-3.20610	1.73470	3.20900
C	-0.39930	-0.72270	0.30710
C	0.92620	-0.08250	0.74930
C	0.79560	0.54750	2.13400
C	-0.48620	1.38970	2.26180
C	-1.69630	0.59240	1.81010
C	-2.99650	1.37260	1.83830
H	1.22160	0.67520	0.02320
H	0.80210	-0.21720	2.90870
H	-1.79180	-0.29610	2.44300
C	2.98780	-1.42980	1.23230
C	-4.28800	2.48890	3.47400
O	-0.41580	2.56300	1.46980
H	-2.93270	2.26630	1.22080
H	-3.81760	0.74810	1.48620
O	-0.31420	-1.03600	-1.04040
H	-0.59490	-1.60370	0.92410
C	-0.83120	-2.24370	-1.48850
H	-0.60930	1.64040	3.31920
H	0.38290	3.04630	1.71740
C	-0.56860	-3.44500	-0.83990
C	-1.55970	-2.21280	-2.67060
O	3.43740	-0.75960	2.13040
C	3.65640	-2.64130	0.68310
C	3.19850	-3.28980	-0.46680
C	3.86370	-4.41260	-0.94080
C	4.98430	-4.89540	-0.27110
C	5.44430	-4.25030	0.87410
C	4.78510	-3.12550	1.34840
H	2.33160	-2.91370	-0.98850
H	3.50740	-4.91050	-1.83340
H	5.49970	-5.77250	-0.64180
H	6.31580	-4.62460	1.39550
H	5.13360	-2.61510	2.23570
C	-4.39350	2.84260	4.91140
O	-5.07440	2.82890	2.61760
C	-5.49670	3.59030	5.32900
C	-5.63210	3.94530	6.66330
C	-4.66600	3.55710	7.58800
C	-3.56430	2.81280	7.17570
C	-3.42550	2.45440	5.84160

H	-6.23920	3.88650	4.60090
H	-6.48890	4.52400	6.98320
H	-4.77160	3.83480	8.62910
H	-2.81270	2.51230	7.89420
H	-2.57020	1.87770	5.52050
H	-2.22580	0.68480	-0.85870
C	-0.99040	2.28660	-3.08680
S	-2.66560	2.29610	-2.26710
O	-3.62620	2.40780	-3.31830
O	-2.74280	0.85210	-1.71550
O	-2.58980	3.27060	-1.22190
F	-0.03460	2.08140	-2.18050
F	-0.93090	1.32720	-4.00850
F	-0.79240	3.46780	-3.67050
C	-2.04020	-3.40240	-3.20560
C	-1.79960	-4.61230	-2.56090
C	-1.06430	-4.62740	-1.38040
H	0.02730	-3.46710	0.06170
H	-1.74530	-1.26400	-3.15380
H	-2.60990	-3.37970	-4.12590
H	-2.17800	-5.53640	-2.97800
H	-0.86050	-5.56450	-0.87820

Structure **3p**, resulting conformar of the benzoate rotation, *tg* E= -2909.151864078891

O	-0.27220	-1.76060	2.63040
C	0.14060	-3.04840	2.58180
O	0.17610	-3.69290	1.55860
C	0.55240	-3.55620	3.91070
C	0.50380	-2.76010	5.05800
C	0.92290	-3.27710	6.27630
C	1.39410	-4.58460	6.35500
C	1.44670	-5.37900	5.21230
C	1.02670	-4.86760	3.99360
H	0.14420	-1.74350	4.99420
H	0.88450	-2.65960	7.16430
H	1.72260	-4.98390	7.30620
H	1.81620	-6.39440	5.27290
H	1.06640	-5.47120	3.09750
O	1.90630	-1.01530	1.01910
O	-0.68470	1.48090	0.16230
O	-4.22010	0.97970	0.61830
C	0.45820	0.65680	-0.14150
C	0.71640	-0.21790	1.08500

C	-0.51010	-1.07130	1.39400
C	-1.74650	-0.19140	1.58210
C	-1.89110	0.72380	0.36860
C	-3.01060	1.73880	0.49530
H	0.90370	0.45080	1.92260
H	-0.68840	-1.79440	0.60080
H	-2.06290	0.10020	-0.51450
C	2.09360	-1.91030	0.02550
C	-5.35650	1.67990	0.78590
O	-1.64690	0.61170	2.74470
H	-2.87310	2.36790	1.37290
H	-3.05080	2.36480	-0.39590
O	1.54220	1.49460	-0.33130
H	0.22120	0.07420	-1.02860
C	1.81970	1.93320	-1.62320
H	-2.62740	-0.83800	1.63060
H	-1.57380	0.02860	3.51090
C	1.94520	1.04110	-2.68170
C	2.04500	3.29150	-1.79710
O	1.41330	-1.94880	-0.97540
C	3.22370	-2.82360	0.31320
C	3.79250	-2.91150	1.58640
C	4.81880	-3.81510	1.82430
C	5.28680	-4.62650	0.79470
C	4.72320	-4.53900	-0.47590
C	3.69080	-3.64420	-0.71570
H	3.42160	-2.28730	2.38610
H	5.25170	-3.88870	2.81350
H	6.08880	-5.32920	0.98260
H	5.08700	-5.17020	-1.27620
H	3.23880	-3.57240	-1.69540
C	-6.54670	0.80430	0.92480
O	-5.38080	2.89080	0.81570
C	-7.79750	1.40790	1.07240
C	-8.93600	0.62650	1.20600
C	-8.83210	-0.76200	1.19420
C	-7.58740	-1.36770	1.04830
C	-6.44560	-0.58940	0.91320
H	-7.86430	2.48720	1.08050
H	-9.90360	1.09800	1.31940
H	-9.72080	-1.37160	1.29880
H	-7.50650	-2.44700	1.03980
H	-5.47910	-1.05860	0.79960
H	-0.45070	2.99390	0.53140
C	1.40230	4.17640	2.60280

S	-0.27610	4.65870	1.94730
O	-0.23330	6.07340	1.75170
O	-0.26590	3.99420	0.55100
O	-1.23260	4.05850	2.82610
F	1.47780	2.85410	2.75720
F	2.35890	4.57270	1.76570
F	1.58350	4.76350	3.78510
C	2.39210	3.76670	-3.05720
C	2.50610	2.89070	-4.13230
C	2.28230	1.53100	-3.93940
H	1.79900	-0.01900	-2.52560
H	1.94390	3.95950	-0.95370
H	2.56710	4.82590	-3.19660
H	2.77260	3.26400	-5.11260
H	2.38140	0.84080	-4.76760

Structure **3p**, TS of the C1-O5 bond breaking, *tg* E= -2909.120895764123

O	-3.00380	-1.29480	-0.69900
C	-3.71510	-2.34750	-0.22930
O	-3.48240	-2.87140	0.83680
C	-4.80260	-2.74860	-1.14750
C	-5.03380	-2.08880	-2.35740
C	-6.09100	-2.48140	-3.16660
C	-6.92140	-3.52680	-2.77200
C	-6.69440	-4.18400	-1.56520
C	-5.63810	-3.79720	-0.75480
H	-4.39340	-1.27260	-2.65810
H	-6.26980	-1.97010	-4.10350
H	-7.74730	-3.82780	-3.40400
H	-7.34270	-4.99390	-1.25720
H	-5.45500	-4.29220	0.18870
O	-4.07850	0.11270	1.24540
O	-0.18280	1.40580	0.93580
O	1.86230	-0.23180	-1.56990
C	-1.95540	0.84920	2.16350
C	-2.71560	0.45560	0.92320
C	-2.03160	-0.70450	0.17120
C	-0.89210	-0.23020	-0.73580
C	0.25770	0.36090	0.07370
C	1.38950	0.88340	-0.79730
H	-2.80060	1.32590	0.27670
H	-1.66970	-1.45620	0.87190
H	0.64270	-0.42700	0.72470

C	-4.25820	-0.45260	2.44420
C	2.80140	0.02390	-2.49610
O	-1.34440	0.74250	-1.66260
H	1.04080	1.67250	-1.46130
H	2.19240	1.27010	-0.17060
O	-2.23900	1.99290	2.65330
H	-1.19860	0.22920	2.61540
C	-1.57620	2.47660	3.81070
H	-0.51680	-1.11510	-1.25700
H	-1.94710	0.32090	-2.28800
C	-1.28710	1.63850	4.87200
C	-1.28450	3.82720	3.80440
O	-3.34210	-0.46630	3.25550
C	-5.59560	-1.01280	2.66160
C	-6.54200	-1.04720	1.63260
C	-7.78230	-1.62260	1.86090
C	-8.08240	-2.15910	3.11010
C	-7.14160	-2.12170	4.13700
C	-5.89800	-1.55190	3.91550
H	-6.30380	-0.63680	0.66240
H	-8.51310	-1.65740	1.06400
H	-9.05150	-2.60900	3.28410
H	-7.37860	-2.53960	5.10650
H	-5.15610	-1.52160	4.70140
C	3.18600	-1.18790	-3.26380
O	3.26890	1.12800	-2.67160
C	4.17310	-1.06390	-4.24380
C	4.55910	-2.16970	-4.98750
C	3.96120	-3.40610	-4.75740
C	2.97660	-3.53420	-3.78180
C	2.58800	-2.43000	-3.03500
H	4.62870	-0.09800	-4.41310
H	5.32480	-2.06930	-5.74580
H	4.26230	-4.26880	-5.33820
H	2.51120	-4.49500	-3.60330
H	1.82370	-2.52810	-2.27780
H	-0.41620	2.23390	0.43770
C	0.42370	5.61370	-1.30370
S	-0.12300	4.89440	0.32190
O	-0.90430	5.95610	0.92500
O	-0.91710	3.73300	-0.10040
O	1.11830	4.55610	0.98980
F	1.15220	4.72690	-1.99840
F	-0.63260	5.96220	-2.05350
F	1.17480	6.70760	-1.10940

C	-0.64140	4.35930	4.91530
C	-0.32060	3.54430	5.99730
C	-0.64620	2.19190	5.97630
H	-1.56590	0.59460	4.85550
H	-1.53950	4.43730	2.94940
H	-0.38960	5.41130	4.92910
H	0.17830	3.96470	6.86060
H	-0.41040	1.56040	6.82260

Structure **4p**, starting conformer, *gg* E= -3079.633019097099

O	0.53980	-1.71900	-2.45050
C	1.77330	-2.09950	-2.86000
O	2.78550	-1.51500	-2.54890
C	1.72040	-3.30840	-3.71490
C	0.54550	-4.04900	-3.87100
C	0.54840	-5.19070	-4.66070
C	1.71720	-5.59520	-5.29940
C	2.88920	-4.85870	-5.14540
C	2.89290	-3.72050	-4.35290
H	-0.35840	-3.73840	-3.36790
H	-0.36060	-5.76650	-4.77590
H	1.71550	-6.48550	-5.91550
H	3.79790	-5.17370	-5.64160
H	3.79710	-3.14240	-4.22120
O	0.80820	-2.20070	0.33260
O	-1.39660	0.72130	0.19110
O	-2.63130	2.57590	-1.68670
C	-0.59040	-0.30190	0.82110
C	-0.19550	-1.34670	-0.22670
C	0.42500	-0.69190	-1.45600
C	-0.42820	0.44990	-2.00660
C	-0.66780	1.41300	-0.84880
C	-1.31750	2.74260	-1.14270
H	-1.06410	-1.93300	-0.51320
H	1.41670	-0.31360	-1.21250
H	0.31530	1.67630	-0.44240
C	0.57780	-3.53410	0.36990
C	-3.32510	3.70770	-1.90330
O	-1.66970	-0.01370	-2.50740
H	-1.37810	3.32140	-0.22110
H	-0.69190	3.28650	-1.85250
S	-1.51160	-1.05440	2.16600
H	0.30170	0.18340	1.22550

C	-1.35590	0.26380	3.42470
H	0.14180	0.96750	-2.78590
H	-1.49600	-0.73340	-3.12850
H	-0.29490	0.42130	3.62050
C	-2.09310	-0.15630	4.68790
H	-1.77840	1.17910	3.01230
O	-0.47500	-4.03650	0.05310
C	1.76730	-4.28790	0.83280
C	3.00320	-3.66930	1.03820
C	4.09350	-4.42290	1.45140
C	3.95600	-5.79140	1.66510
C	2.72500	-6.41030	1.46200
C	1.63430	-5.66220	1.04450
H	3.10950	-2.60820	0.86690
H	5.05130	-3.94310	1.60550
H	4.80800	-6.37610	1.98840
H	2.61780	-7.47440	1.62790
H	0.67380	-6.13060	0.87950
C	-4.67010	3.45250	-2.47360
O	-2.88050	4.80820	-1.65140
C	-5.53520	4.53590	-2.64640
C	-6.80280	4.33490	-3.17250
C	-7.21240	3.05240	-3.53080
C	-6.35140	1.97200	-3.36250
C	-5.08210	2.16880	-2.83600
H	-5.20530	5.52600	-2.36280
H	-7.47260	5.17520	-3.30260
H	-8.20270	2.89650	-3.94020
H	-6.66790	0.97420	-3.63650
H	-4.41660	1.33160	-2.70210
H	-1.67780	-1.07710	5.10130
H	-3.15460	-0.31590	4.49100
H	-2.00170	0.62690	5.44240
H	-2.87440	0.31450	-0.10860
C	-5.68850	-1.50360	0.45380
S	-4.33990	-1.17860	-0.78840
O	-5.00300	-1.04310	-2.04960
O	-3.88150	0.19420	-0.25920
O	-3.35000	-2.19760	-0.60270
F	-5.18590	-1.57220	1.68450
F	-6.60220	-0.53380	0.40890
F	-6.27070	-2.66580	0.15450

Structure **4p**, TS of the benzoate rotation, *gg*   E= -3079.620799907730

O	0.25210	-2.12510	-2.57590
C	1.19820	-2.21830	-3.53490
O	2.07700	-1.39870	-3.67660
C	1.01680	-3.42050	-4.38250
C	0.03520	-4.37650	-4.10890
C	-0.08840	-5.49410	-4.92320
C	0.76280	-5.66280	-6.01170
C	1.74170	-4.71130	-6.28700
C	1.86930	-3.59380	-5.47520
H	-0.62190	-4.24630	-3.26150
H	-0.84780	-6.23470	-4.70820
H	0.66420	-6.53560	-6.64470
H	2.40390	-4.84210	-7.13300
H	2.62650	-2.84860	-5.67690
O	0.73970	-2.47130	0.49960
O	-1.23650	0.54360	0.09850
O	-2.39130	2.44010	-1.75160
C	-0.48850	-0.51300	0.72470
C	-0.14780	-1.63970	-0.27440
C	0.39760	-1.08750	-1.59420
C	-0.39140	0.13350	-2.10170
C	-0.48790	1.14800	-0.97720
C	-1.03990	2.51850	-1.28510
H	-1.03910	-2.22870	-0.48600
H	1.44570	-0.81390	-1.49940
H	0.52880	1.33540	-0.61390
C	1.79780	-3.18900	0.08490
C	-3.02580	3.61280	-1.92690
O	-1.69800	-0.23090	-2.51220
H	-1.00350	3.12870	-0.38290
H	-0.41370	2.98690	-2.04640
S	-1.43280	-1.14790	2.12060
H	0.44470	-0.08140	1.09630
C	-1.20270	0.23550	3.29390
H	0.17170	0.57010	-2.93250
H	-1.62760	-1.00400	-3.08790
H	-0.13360	0.35130	3.47500
C	-1.94940	-0.06970	4.58440
H	-1.58220	1.14470	2.82940
O	2.22240	-3.22500	-1.04560
C	2.39980	-3.93800	1.22270
C	1.88020	-3.86980	2.51820
C	2.48230	-4.58660	3.54380
C	3.60110	-5.37320	3.28430

C	4.12020	-5.44360	1.99410
C	3.52210	-4.72870	0.96640
H	1.01140	-3.25880	2.71730
H	2.07820	-4.53210	4.54650
H	4.06800	-5.93090	4.08630
H	4.98980	-6.05510	1.79090
H	3.91540	-4.77470	-0.03980
C	-4.41610	3.43990	-2.41420
O	-2.50100	4.68340	-1.70260
C	-5.22910	4.57090	-2.52250
C	-6.53680	4.44580	-2.96810
C	-7.03840	3.19210	-3.31110
C	-6.22920	2.06450	-3.20810
C	-4.92040	2.18520	-2.76150
H	-4.82810	5.53810	-2.25210
H	-7.16610	5.32280	-3.04760
H	-8.05970	3.09540	-3.65780
H	-6.61640	1.08890	-3.47130
H	-4.29500	1.31140	-2.67850
H	-1.57740	-0.98330	5.05130
H	-3.01910	-0.18730	4.40310
H	-1.81290	0.75240	5.28900
H	-2.73570	0.21940	-0.15910
C	-5.68760	-1.41710	0.41020
S	-4.28340	-1.20670	-0.79340
O	-4.89960	-1.04970	-2.07590
O	-3.75400	0.13570	-0.25490
O	-3.37110	-2.28940	-0.56630
F	-5.22610	-1.53820	1.65320
F	-6.51000	-0.36990	0.34930
F	-6.36280	-2.52080	0.08600

Structure **4p**, resulting conformar of the benzoate rotation, *gg* E= -3079.631049617479

O	1.73490	0.55350	-0.00920
C	2.71990	0.26110	0.87110
O	2.50630	-0.11110	2.00230
C	4.06690	0.43700	0.28080
C	4.24810	0.77700	-1.06260
C	5.53140	0.90190	-1.57700
C	6.63610	0.68860	-0.75730
C	6.45820	0.34800	0.58140
C	5.17800	0.22140	1.09960
H	3.39020	0.93330	-1.70010

H	5.67020	1.16270	-2.61820
H	7.63560	0.78530	-1.16200
H	7.31710	0.17970	1.21790
H	5.02510	-0.04760	2.13560
O	0.93660	-2.15080	0.10490
O	-2.16060	-0.31120	-0.81810
O	-3.06930	2.32030	-1.62810
C	-1.43860	-1.44160	-0.26520
C	0.05840	-1.11270	-0.35410
C	0.38220	0.21380	0.33450
C	-0.51990	1.34260	-0.17330
C	-1.96180	0.87510	-0.01510
C	-3.07140	1.86530	-0.27000
H	0.31610	-1.01730	-1.40550
H	0.28930	0.12770	1.41450
H	-2.09090	0.58720	1.03400
C	0.86000	-2.61810	1.36870
C	-4.11200	3.08930	-1.99000
O	-0.26660	1.64030	-1.53510
H	-4.02720	1.39060	-0.05020
H	-2.94420	2.71560	0.40270
S	-1.83640	-2.92930	-1.18530
H	-1.76310	-1.56260	0.76530
C	-3.49340	-3.27290	-0.48910
H	-0.36130	2.22400	0.45730
H	0.67740	1.82210	-1.63380
H	-3.38000	-3.42480	0.58460
C	-4.08180	-4.50440	-1.16160
H	-4.12040	-2.39840	-0.65800
O	-0.03990	-2.34340	2.13060
C	2.00490	-3.49990	1.69760
C	3.13140	-3.58100	0.87500
C	4.19770	-4.39110	1.24030
C	4.14260	-5.12740	2.42030
C	3.02010	-5.04980	3.24100
C	1.95590	-4.23470	2.88400
H	3.17530	-3.00240	-0.03610
H	5.07280	-4.44630	0.60590
H	4.97490	-5.76010	2.70180
H	2.97730	-5.62220	4.15850
H	1.08140	-4.16000	3.51580
C	-4.03920	3.52260	-3.40660
O	-5.00700	3.38420	-1.22560
C	-5.11760	4.23350	-3.93940
C	-5.08890	4.65090	-5.26190

C	-3.98300	4.36290	-6.05870
C	-2.90600	3.65810	-5.52940
C	-2.93100	3.23820	-4.20640
H	-5.97080	4.44880	-3.31090
H	-5.92710	5.19840	-5.67320
H	-3.96210	4.68770	-7.09150
H	-2.04690	3.43020	-6.14670
H	-2.09850	2.68850	-3.79820
H	-3.44290	-5.37760	-1.01810
H	-4.21170	-4.34440	-2.23320
H	-5.05980	-4.72450	-0.73020
H	-2.09900	-0.17220	-2.36420
C	-1.73070	-1.32760	-5.56280
S	-0.96930	-0.23250	-4.26380
O	-0.62630	0.98230	-4.93820
O	-2.21490	-0.04460	-3.37840
O	0.05440	-1.00630	-3.62290
F	-2.17670	-2.45950	-5.02200
F	-2.74200	-0.70080	-6.16280
F	-0.79650	-1.61650	-6.47040

Structure **4p**, TS of the C1-O5 bond breaking, *gg* E= -3079.604595193157

O	1.54000	0.52760	0.14420
C	2.41840	0.13960	1.09570
O	2.08430	-0.47400	2.08540
C	3.80950	0.52390	0.77230
C	4.12910	1.26510	-0.36840
C	5.45380	1.57900	-0.63890
C	6.46200	1.15220	0.22100
C	6.14630	0.41010	1.35640
C	4.82410	0.09730	1.63310
H	3.34610	1.59110	-1.03750
H	5.70060	2.15460	-1.52150
H	7.49450	1.39630	0.00520
H	6.93080	0.07540	2.02240
H	4.56450	-0.48340	2.50720
O	1.20250	-1.99470	-0.37140
O	-2.69250	0.01680	-0.67540
O	-3.03580	2.63800	-1.70650
C	-1.14130	-2.06420	0.03420
C	-0.01560	-1.19890	-0.49060
C	0.16820	0.12240	0.28750

C	-0.64440	1.29860	-0.27320
C	-2.14560	1.14900	-0.02720
C	-2.95230	2.41140	-0.29130
H	-0.12350	-1.01640	-1.55680
H	-0.05310	-0.01880	1.34420
H	-2.25650	0.96440	1.04580
C	1.12740	-2.85730	0.62000
C	-3.64900	3.75980	-2.10740
O	-0.39700	1.47270	-1.65660
H	-3.96120	2.28370	0.10250
H	-2.49200	3.27180	0.19590
S	-1.86730	-3.16770	-1.02290
H	-1.74810	-1.68490	0.84220
C	-3.29400	-3.74330	-0.03010
H	-0.30970	2.17630	0.29170
H	0.54220	1.65930	-1.78300
H	-2.90700	-4.34460	0.79080
C	-4.22640	-4.54490	-0.92550
H	-3.78160	-2.85450	0.36830
O	0.02430	-3.01240	1.17970
C	2.34010	-3.57520	0.97800
C	3.56140	-3.24200	0.38030
C	4.71120	-3.91390	0.76000
C	4.64610	-4.91440	1.72700
C	3.42980	-5.24810	2.31930
C	2.27450	-4.57980	1.94970
H	3.60620	-2.46070	-0.36430
H	5.65880	-3.65620	0.30640
H	5.54770	-5.43610	2.02150
H	3.38600	-6.02680	3.06890
H	1.32470	-4.82590	2.40360
C	-3.68190	3.89120	-3.58640
O	-4.11540	4.57110	-1.33310
C	-4.26140	5.03500	-4.14090
C	-4.30620	5.19310	-5.51860
C	-3.77400	4.20970	-6.34980
C	-3.19790	3.06830	-5.80000
C	-3.15010	2.90610	-4.42180
H	-4.67110	5.79080	-3.48490
H	-4.75480	6.08090	-5.94560
H	-3.80950	4.33430	-7.42490
H	-2.78330	2.30010	-6.43960
H	-2.70890	2.01660	-3.99990
H	-3.72350	-5.41760	-1.34500
H	-4.60710	-3.93550	-1.74610

H	-5.07460	-4.89390	-0.33500
H	-2.58970	0.07630	-1.64790
C	-2.38220	-2.16410	-5.24950
S	-1.56560	-0.80280	-4.28000
O	-1.17240	0.15320	-5.29670
O	-2.64390	-0.35620	-3.40240
O	-0.47000	-1.48360	-3.60780
F	-2.82480	-3.13680	-4.43640
F	-3.43200	-1.69780	-5.94360
F	-1.52100	-2.71360	-6.12020

Structure 4**p**, starting conformer, tg E= -3079.634331888592

H	-0.14790	0.24690	0.41700
C	0.31850	2.00070	3.16690
S	-0.24510	0.32280	2.58730
O	-1.37800	-0.00780	3.39450
O	-0.70980	0.68420	1.15920
O	0.91950	-0.51200	2.57500
F	1.32600	2.43680	2.41190
F	-0.68460	2.87490	3.10400
F	0.73270	1.89990	4.43040
O	-1.24000	0.18420	-4.28170
C	-1.30310	-0.42510	-5.49500
O	-0.38140	-1.04900	-5.96690
C	-2.63100	-0.26960	-6.13070
C	-3.67100	0.42710	-5.51030
C	-4.92190	0.47850	-6.10930
C	-5.14220	-0.16780	-7.32190
C	-4.10720	-0.86320	-7.94220
C	-2.85470	-0.91270	-7.35070
H	-3.50380	0.90990	-4.55900
H	-5.72790	1.01450	-5.62540
H	-6.12200	-0.13450	-7.78140
H	-4.28080	-1.37220	-8.88130
H	-2.04660	-1.46180	-7.81320
O	-1.50870	-2.24940	-3.03400
O	0.65350	-0.44050	-0.68900
O	2.91210	2.15560	-1.75050
C	0.03940	-1.61420	-1.27440
C	-0.95840	-1.13790	-2.32610
C	-0.24250	-0.27130	-3.35880
C	0.45110	0.91480	-2.70460
C	1.38920	0.36530	-1.63400

C	2.09110	1.43570	-0.82140
H	-1.75590	-0.57510	-1.84530
H	0.49460	-0.86880	-3.89440
H	2.13500	-0.27230	-2.11720
C	-2.85790	-2.29620	-3.18580
C	3.57990	3.21870	-1.26580
O	-0.46660	1.79930	-2.08320
H	1.37840	2.11410	-0.35690
H	2.70880	0.97780	-0.04950
S	-0.77550	-2.57360	0.00530
H	0.83290	-2.21120	-1.73160
C	0.68070	-3.33980	0.80450
H	1.04950	1.43670	-3.45690
H	-1.04970	2.16520	-2.76030
H	1.37320	-2.54360	1.06920
C	0.22920	-4.09580	2.04510
H	1.15300	-4.00550	0.08180
O	-3.61970	-1.63500	-2.52010
C	-3.26240	-3.20620	-4.28140
C	-2.32920	-3.94050	-5.01730
C	-2.75160	-4.72600	-6.08050
C	-4.10080	-4.77700	-6.41810
C	-5.03280	-4.04540	-5.68650
C	-4.61650	-3.26430	-4.61960
H	-1.28060	-3.88450	-4.76590
H	-2.02720	-5.29200	-6.65160
H	-4.42580	-5.38310	-7.25430
H	-6.08110	-4.07950	-5.95330
H	-5.32740	-2.68070	-4.05170
C	4.36360	3.92160	-2.31170
O	3.53030	3.54690	-0.10090
C	5.12650	5.02840	-1.93250
C	5.86980	5.71840	-2.87890
C	5.85540	5.30780	-4.20950
C	5.09600	4.20560	-4.59170
C	4.35110	3.51170	-3.64760
H	5.12810	5.33760	-0.89630
H	6.46000	6.57540	-2.58130
H	6.43530	5.84690	-4.94800
H	5.08400	3.88710	-5.62600
H	3.76080	2.65670	-3.94350
H	1.09190	-4.56750	2.51870
H	-0.22970	-3.42050	2.76850
H	-0.49090	-4.87750	1.79610

Structure **4p**, TS of the benzoate rotation, *tg* E= -3079.612822189222

H	-0.33030	0.55990	0.49740
C	0.64740	2.34810	3.13590
S	-0.27560	0.80310	2.65610
O	-1.42860	0.76180	3.50210
O	-0.68530	1.19800	1.22030
O	0.68270	-0.26220	2.66950
F	1.72300	2.50280	2.36550
F	-0.13740	3.41770	3.01030
F	1.03440	2.23740	4.40750
O	-1.73350	0.36140	-4.09110
C	-1.67840	0.14740	-5.42490
O	-0.76490	-0.43180	-5.96840
C	-2.86380	0.69440	-6.12650
C	-3.88950	1.35540	-5.44530
C	-4.98370	1.84290	-6.14680
C	-5.06130	1.67260	-7.52620
C	-4.04120	1.01350	-8.20760
C	-2.94520	0.52640	-7.51110
H	-3.82940	1.48480	-4.37450
H	-5.77700	2.35470	-5.61770
H	-5.91670	2.05290	-8.07020
H	-4.10180	0.88010	-9.27990
H	-2.14680	0.01210	-8.02800
O	-1.57230	-2.29010	-3.18970
O	0.26490	-0.34010	-0.60920
O	2.78530	1.85960	-1.93310
C	-0.51610	-1.48620	-1.03860
C	-1.35970	-1.17120	-2.29890
C	-0.69310	-0.17880	-3.26120
C	0.06740	0.95870	-2.60840
C	1.03350	0.32390	-1.62310
C	1.93920	1.31220	-0.91230
H	-2.33470	-0.78650	-1.99800
H	0.00370	-0.73450	-3.88590
H	1.64990	-0.41350	-2.14530
C	-1.94750	-3.54780	-2.89100
C	3.63520	2.83220	-1.55680
O	-0.77300	1.85130	-1.89620
H	1.37100	2.11030	-0.43890
H	2.53910	0.80190	-0.15930
S	-1.56400	-1.90970	0.36940
H	0.17720	-2.30130	-1.25070

C	-0.46020	-3.05010	1.26910
H	0.63220	1.48560	-3.38300
H	-1.38070	2.26410	-2.52240
H	0.49120	-2.54710	1.43170
C	-1.10390	-3.42690	2.59530
H	-0.30650	-3.92690	0.63960
O	-2.02080	-4.00670	-1.77750
C	-2.23670	-4.32160	-4.13120
C	-2.10490	-3.77000	-5.40830
C	-2.39070	-4.53970	-6.52820
C	-2.81050	-5.85860	-6.38240
C	-2.94240	-6.41140	-5.11110
C	-2.65540	-5.64680	-3.98970
H	-1.77440	-2.74920	-5.52560
H	-2.28560	-4.10900	-7.51570
H	-3.03390	-6.45550	-7.25770
H	-3.26850	-7.43700	-4.99550
H	-2.75390	-6.06470	-2.99730
C	4.43360	3.35840	-2.69190
O	3.72110	3.22000	-0.41240
C	5.38480	4.34670	-2.42840
C	6.14920	4.87000	-3.46100
C	5.96770	4.41060	-4.76300
C	5.02040	3.42620	-5.03010
C	4.25420	2.89880	-3.99940
H	5.51530	4.69560	-1.41330
H	6.88570	5.63530	-3.25270
H	6.56400	4.81970	-5.56880
H	4.87870	3.06960	-6.04220
H	3.51830	2.13540	-4.20610
H	-0.45710	-4.12420	3.13060
H	-1.25070	-2.54730	3.22340
H	-2.07130	-3.90970	2.44430

Structure **4p**, resulting conformar of the benzoate rotation, *tg*   E= -3079.632597645045

H	-0.89330	1.72450	-1.67930
C	-0.71120	4.90230	-1.11500
S	-1.97010	3.61020	-1.57720
O	-2.80190	4.21620	-2.56950
O	-1.05770	2.56280	-2.25220
O	-2.51780	3.13030	-0.34360
F	0.17170	4.40070	-0.25160
F	-0.06630	5.33250	-2.19830

F	-1.34230	5.92910	-0.54440
O	1.30570	-1.97950	-3.50470
C	1.39200	-3.27830	-3.87270
O	1.07040	-4.19370	-3.15010
C	1.90780	-3.43500	-5.25260
C	2.23830	-2.33640	-6.05050
C	2.69640	-2.53520	-7.34560
C	2.82340	-3.82610	-7.85080
C	2.49200	-4.92270	-7.05880
C	2.03570	-4.72890	-5.76350
H	2.13250	-1.33490	-5.65960
H	2.95200	-1.68340	-7.96240
H	3.17870	-3.97770	-8.86220
H	2.58810	-5.92610	-7.45260
H	1.77020	-5.57050	-5.13890
O	-1.48170	-2.27920	-3.31410
O	-0.71080	0.42270	-0.88790
O	2.46920	0.77580	0.77300
C	-1.49840	-0.69770	-1.36340
C	-0.79170	-1.23560	-2.61470
C	0.63720	-1.65620	-2.27580
C	1.40780	-0.50800	-1.62300
C	0.60010	0.01710	-0.44220
C	1.21500	1.22310	0.24060
H	-0.74770	-0.42930	-3.34590
H	0.63770	-2.52300	-1.61850
H	0.47640	-0.78840	0.28720
C	-1.82580	-3.42570	-2.68750
C	3.24430	1.71070	1.35260
O	1.61560	0.56770	-2.52370
H	1.38160	2.03610	-0.46250
H	0.56730	1.56950	1.04540
S	-3.16740	-0.16220	-1.74520
H	-1.52760	-1.43680	-0.56660
C	-3.80920	0.06000	-0.04720
H	2.36120	-0.89500	-1.25270
H	2.15060	0.24920	-3.26170
H	-3.14210	0.74350	0.47370
C	-5.21950	0.62620	-0.11190
H	-3.79180	-0.91080	0.44860
O	-1.75620	-3.57940	-1.48930
C	-2.29180	-4.45470	-3.64630
C	-2.10180	-4.31760	-5.02370
C	-2.51590	-5.32600	-5.88280
C	-3.12710	-6.46870	-5.37460

C	-3.32000	-6.60670	-4.00230
C	-2.89900	-5.60550	-3.13940
H	-1.61970	-3.43390	-5.41540
H	-2.35960	-5.22190	-6.94870
H	-3.45110	-7.25280	-6.04730
H	-3.79530	-7.49510	-3.60710
H	-3.03590	-5.70350	-2.07120
C	4.53410	1.15850	1.83650
O	2.90520	2.86900	1.45710
C	5.41420	2.01820	2.49770
C	6.63240	1.54450	2.96250
C	6.97980	0.21000	2.76860
C	6.10610	-0.65000	2.10930
C	4.88520	-0.18030	1.64400
H	5.13320	3.05250	2.64100
H	7.31120	2.21360	3.47530
H	7.93100	-0.15920	3.13090
H	6.37650	-1.68690	1.95760
H	4.20670	-0.84720	1.13210
H	-5.60960	0.74900	0.90000
H	-5.22750	1.60170	-0.60000
H	-5.89210	-0.03910	-0.65650

Structure **4p**, TS of the C1-O5 bond breaking, *tg* E= -3079.601135638815

H	-0.76500	1.46010	-0.62230
C	-2.15100	5.21020	-0.57710
S	-2.59810	3.46180	-1.02720
O	-3.58500	3.62300	-2.07790
O	-1.31600	2.91350	-1.47610
O	-3.08820	2.90450	0.21990
F	-1.21920	5.23460	0.38910
F	-1.66430	5.87220	-1.63830
F	-3.22710	5.87720	-0.13110
O	1.23280	-2.00720	-2.99940
C	1.16870	-3.33950	-3.21810
O	0.49350	-4.08310	-2.54030
C	1.98240	-3.76430	-4.37780
C	2.76730	-2.86440	-5.10370
C	3.49240	-3.30960	-6.20040
C	3.43390	-4.64790	-6.57940
C	2.64980	-5.54580	-5.85940
C	1.92660	-5.10680	-4.76090
H	2.80720	-1.82530	-4.81100

H	4.10160	-2.61280	-6.76120
H	3.99840	-4.99100	-7.43730
H	2.60200	-6.58540	-6.15650
H	1.30820	-5.79080	-4.19670
O	-1.25740	-1.91290	-3.69460
O	-0.67380	0.61850	-0.12360
O	2.85540	0.80530	0.71080
C	-2.11240	-1.07780	-1.63300
C	-0.93420	-1.02480	-2.58590
C	0.40950	-1.46080	-1.95520
C	1.22500	-0.29930	-1.37290
C	0.67480	0.20250	-0.04020
C	1.52240	1.32250	0.54630
H	-0.83310	-0.04110	-3.03490
H	0.24410	-2.22430	-1.19670
H	0.69070	-0.64570	0.64920
C	-2.07100	-2.88190	-3.32630
C	3.79770	1.66150	1.13340
O	1.30710	0.78640	-2.28400
H	1.54380	2.18660	-0.11640
H	1.12070	1.62190	1.51380
S	-3.41560	-0.04270	-1.92990
H	-1.93320	-1.37210	-0.61090
C	-4.39220	-0.20840	-0.38860
H	2.22040	-0.70760	-1.17900
H	1.91060	0.55050	-2.99930
H	-3.75080	0.13380	0.42130
C	-5.64990	0.63730	-0.50670
H	-4.62360	-1.26440	-0.26120
O	-2.58370	-2.81740	-2.19280
C	-2.31670	-3.94850	-4.28300
C	-1.59210	-4.00650	-5.47940
C	-1.81570	-5.05060	-6.36120
C	-2.75770	-6.03020	-6.05600
C	-3.48100	-5.97070	-4.86660
C	-3.26220	-4.93330	-3.97560
H	-0.85820	-3.24740	-5.70750
H	-1.25410	-5.10440	-7.28410
H	-2.92840	-6.84510	-6.74790
H	-4.21170	-6.73430	-4.63610
H	-3.81350	-4.87690	-3.04740
C	5.14010	1.03010	1.22240
O	3.56350	2.81940	1.40580
C	6.21010	1.80920	1.66780
C	7.48090	1.25990	1.76070

C	7.69120	-0.07100	1.40940
C	6.62760	-0.85130	0.96480
C	5.35450	-0.30520	0.87060
H	6.03370	2.84160	1.93650
H	8.30720	1.86750	2.10640
H	8.68290	-0.49940	1.48180
H	6.79080	-1.88580	0.69110
H	4.52850	-0.90990	0.52520
H	-6.22180	0.54860	0.41810
H	-5.40240	1.68760	-0.65550
H	-6.28000	0.30050	-1.33130

Structure **1p**, starting conformer, *gt* E= -3216.538078063406

O	-2.77720	4.37570	-2.23400
O	-1.47120	2.91080	-0.87830
O	-0.99370	-0.65020	0.13910
O	-2.25180	0.96210	1.14570
O	1.58280	0.30500	-0.03040
O	2.68970	-1.84540	0.44920
O	0.91530	-0.98930	1.92900
O	-2.34710	2.10960	-3.06520
O	3.04970	0.14580	1.85450
O	-3.88520	2.45770	-1.20700
C	-1.71570	0.59490	-0.06350
C	-0.76840	1.71300	-0.53590
C	0.16020	1.24180	-1.67190
C	0.85700	-0.05110	-1.25080
C	-0.27280	-1.06560	-1.02580
C	-3.42080	0.22210	1.51960
H	-0.16000	2.00750	0.31450
H	-0.45170	0.98810	-2.53400
H	1.57700	-0.38660	-1.99400
H	-0.92980	-0.98790	-1.89770
H	-2.48950	0.38430	-0.80740
O	0.99300	2.27350	-2.19350
H	-3.18100	-0.83080	1.67740
H	-4.18690	0.31700	0.74700
S	-2.72770	2.95450	-1.93930
S	2.17530	-0.65240	1.05570
C	0.08600	-2.53230	-0.91300
O	-1.13290	-3.25370	-1.14020
S	-1.07890	-4.86810	-0.87340
O	-2.44500	-5.25020	-1.18720

O	-0.07620	-5.38480	-1.79770
O	-0.71210	-5.02320	0.52970
H	0.82090	-2.78300	-1.67980
H	0.48700	-2.79510	0.06160
H	0.06970	-0.84170	1.40320
H	-3.77740	0.65930	2.44910
S	2.23620	3.03520	-1.42480
O	2.37900	4.20390	-2.27880
O	1.78560	3.33230	-0.07290
O	3.36320	2.11630	-1.49420

Structure **1p**, TS of the C1-O5 bond breaking, *gt* E= -3216.512863756629

O	-2.84180	4.72600	-1.90160
O	-1.27950	3.19240	-0.91750
O	-1.14810	-1.08320	0.23100
O	-2.41050	1.30790	1.16040
O	1.22300	0.25680	0.20890
O	2.91880	-1.54110	0.21130
O	1.08420	-1.56380	1.82740
O	-2.23150	2.72230	-3.17310
O	2.81620	0.14770	1.98900
O	-3.60580	2.50840	-1.17040
C	-2.04310	1.09570	-0.02220
C	-0.83980	1.89690	-0.49940
C	-0.01330	1.17460	-1.58800
C	0.66080	-0.10250	-1.06010
C	-0.37410	-1.24400	-0.95620
C	-3.60170	0.63550	1.67780
H	-0.21110	2.09020	0.36370
H	-0.67840	0.88250	-2.39850
H	1.45690	-0.37630	-1.75210
H	-1.07570	-1.13110	-1.78550
H	-2.60900	0.43460	-0.65980
O	0.87410	2.08390	-2.23560
H	-3.28570	0.12900	2.58460
H	-3.97730	-0.06220	0.93470
S	-2.60300	3.29990	-1.89520
S	2.09670	-0.76460	1.12390
C	0.21680	-2.63790	-1.08280
O	-0.88380	-3.54010	-1.28920
S	-0.60040	-5.11500	-0.97250
O	-1.87210	-5.71320	-1.34740
O	0.52400	-5.49590	-1.82130

O	-0.29850	-5.18790	0.45300
H	0.88330	-2.67330	-1.94670
H	0.76790	-2.92140	-0.19050
H	-0.53710	-1.20240	0.99040
H	-4.31960	1.42100	1.89510
S	2.22660	2.74890	-1.56530
O	2.49740	3.79380	-2.53990
O	1.84590	3.23910	-0.24890
O	3.22300	1.68830	-1.54670

Structure **1p**, starting conformer, *tg* E= -3216.536726603714

O	-2.98240	4.38820	-1.01060
O	-1.04020	3.00740	-0.87900
O	0.18370	-0.37640	0.12510
O	0.13330	1.54680	1.35360
O	1.62110	0.15760	-2.15310
O	2.80350	-2.00460	-2.33050
O	2.79150	-0.74740	-0.21320
O	-3.26090	1.96940	-1.30660
O	4.01180	0.12820	-2.11450
O	-2.76190	2.87980	0.89700
C	-0.46580	0.91600	0.29290
C	-0.28700	1.79360	-0.95970
C	-0.57700	1.02030	-2.26080
C	0.22630	-0.27790	-2.26380
C	-0.27180	-1.06150	-1.04230
C	-0.30170	1.08240	2.63780
H	0.74460	2.13300	-0.97700
H	-1.62430	0.72750	-2.26140
H	0.10990	-0.83440	-3.19000
H	-1.36450	-1.02340	-1.08370
H	-1.52120	0.70450	0.48550
O	-0.49430	1.82740	-3.43300
H	-0.01140	0.04200	2.79430
H	-1.38560	1.18450	2.72400
S	-2.65020	3.05320	-0.54530
S	2.88410	-0.68340	-1.78160
C	0.09310	-2.53140	-0.90910
O	-0.33940	-3.15580	-2.11910
S	-0.34030	-4.79500	-2.13210
O	-0.75400	-5.06180	-3.49870
O	1.02720	-5.19040	-1.81910
O	-1.30760	-5.19130	-1.11580

H	1.15630	-2.69940	-0.76400
H	-0.45290	-2.93390	-0.05410
H	1.86120	-0.53160	0.09560
H	0.18820	1.71350	3.37550
S	0.85980	2.51330	-4.07380
O	0.24830	3.49870	-4.95200
O	1.61320	3.08350	-2.96620
O	1.54890	1.44400	-4.78170

Structure **1p**, TS of the C1-O5 bond breaking, *tg*   E= -3216.511734219054

O	-2.84480	4.64290	-0.80460
O	-0.99290	3.14270	-1.06050
O	0.35300	-0.71710	0.38310
O	-0.04400	1.85470	1.43260
O	1.66650	0.21130	-1.80200
O	2.87780	-1.74280	-2.71320
O	2.96130	-1.23120	-0.32320
O	-3.26790	2.45780	-1.82530
O	4.05110	0.26030	-1.91970
O	-2.75060	2.59810	0.55310
C	-0.64240	1.32500	0.46080
C	-0.30660	1.89760	-0.90910
C	-0.56850	0.92080	-2.07730
C	0.34570	-0.30970	-2.00660
C	-0.11740	-1.23170	-0.86140
C	-0.37300	1.44110	2.79560
H	0.74390	2.17120	-0.89990
H	-1.59560	0.56470	-2.02040
H	0.29490	-0.83970	-2.95750
H	-1.20680	-1.18350	-0.81530
H	-1.45120	0.63130	0.63340
O	-0.55250	1.60180	-3.32850
H	0.49460	0.90340	3.16790
H	-1.26100	0.81510	2.78200
S	-2.61880	3.21410	-0.77080
S	2.99890	-0.71010	-1.69850
C	0.22520	-2.70800	-1.01630
O	-0.55970	-3.17590	-2.12340
S	-0.46590	-4.77210	-2.46330
O	-1.38520	-4.87520	-3.58430
O	0.93010	-5.02730	-2.79760
O	-0.90690	-5.45710	-1.25310
H	1.28020	-2.87640	-1.21200

H	-0.06920	-3.22730	-0.10250
H	1.33710	-0.75000	0.35770
H	-0.53470	2.35880	3.35210
S	0.76670	2.26810	-4.06060
O	0.10340	3.11360	-5.04120
O	1.49520	2.99880	-3.03400
O	1.50150	1.15720	-4.64620
